# Genomic diversity and the domestication history of cotton (*Gossypium hirsutum*)

**DOI:** 10.1073/pnas.2607107123

**Published:** 2026-05-18

**Authors:** Weixuan Ning, Mark A. Arick, Joshua A. Udall, Chuan-Yu Hsu, Luis Abdala-Roberts, Uriel Solís-Rodríguez, Yeyson Briones-May, Zenaida V. Magbanua, Olga Pechanova, Carlos Bustos-Segura, Mary V. Clancy, Sandra Díaz-Cruz, Alejandra Garnica-Cabrera, Marine Mamin, John Z. Yu, Ted C. J. Turlings, Guanjing Hu, Daniel G. Peterson, Corrinne E. Grover, Jonathan F. Wendel

**Affiliations:** ^a^Department of Ecology, Evolution, and Organismal Biology, Iowa State University, Ames, IA 50011; ^b^Institute for Genomics, Biocomputing and Biotechnology, Mississippi State University, Mississippi State, MS 39762; ^c^Crop Germplasm Research Unit, United States Department of Agriculture/Agricultural Research Service, College Station, TX 77845; ^d^Universidad Nacional Autónoma de México, Escuela Nacional de Estudios Superiores Unidad Mérida, Departamento de Sistemas y Procesos Naturales, Ucú, Yucatán 97357, México; ^e^Departamento de Ecología Tropical, Campus de Ciencias Biológicas y Agropecuarias, Universidad Autónoma de Yucatán, Itzimná 97000, México; ^f^Department of Biochemistry, Nutrition, and Health Promotion, Mississippi State University, Mississippi State, MS 39762; ^g^Institute of Biology, Fundamental and Applied Research in Chemical Ecology, University of Neuchâtel, Neuchâtel 2000, Switzerland; ^h^State Key Laboratory of Cotton Bio-breeding and Integrated Utilization, School of Life Sciences and College of Agriculture, Henan University, Kaifeng 475004, China; ^i^Department of Entomology, The Pennsylvania State University, University Park, PA 16802; ^j^Shenzhen Branch, Guangdong Laboratory of Lingnan Modern Agriculture, Key Laboratory of Synthetic Biology, Ministry of Agriculture and Rural Affairs, Agricultural Genomics Institute at Shenzhen, Chinese Academy of Agricultural Sciences, Shenzhen 518120, China; ^k^State Key Laboratory of Cotton Bio-breeding and Integrated Utilization, Institute of Cotton Research, Chinese Academy of Agricultural Sciences, Anyang 455000, China

**Keywords:** domestication, introgression, polyploidy, population genomics, genetic bottlenecks

## Abstract

*Gossypium hirsutum* is the leading fiber crop globally, but its origin as a domesticated plant and patterns of diversity in the wild remain to be elucidated. Here, we use extensive sampling of wild populations and comparative genome sequence data to illuminate the scope and patterning of wild cotton diversity across its native range. Analyses confirm the hypothesis that the Yucatán Peninsula (México) is the center of domestication, from which the original perennial forms and later modern annualized cultivars were derived. Population structure and phylogenomic analyses indicate that northwestern Yucatán harbors greater genetic diversity relative to smaller, geographically dispersed populations in northeastern Yucatán and the Caribbean basin. Genetic load and transposable element burden also are the lowest in northwestern Yucatán relative to other regions, consistent with its greater diversity and reflecting the effects of historical genetic bottlenecks in other populations. Populations from Florida and elsewhere in the Caribbean basin maintain unique pockets of diversity. Analyses of selection suggest that cotton domestication entailed long-term accumulation of mutations with relatively minor phenotypic effects, as opposed to a more punctuated process involving major domestication genes. Our study quantifies the scope and scale of genomic diversity in wild cotton, the origin of the cultivated gene pool, and the likely ecological and anthropogenic processes that shaped extant diversity and modern geographic patterning.

## Significance

Cultivated cotton is the most important source of natural textile fiber globally, yet the geographic origin of the modern gene pool and the amount of diversity within and among natural populations have been unclear. Here, we use extensive sampling of wild populations and comparative genome sequence to quantify the amount and patterning of wild cotton diversity across its native range. We show that the Yucatán Peninsula (México) was the center of original domestication, from which later modern annualized cultivars were derived. Our study quantifies diversity in wild cotton populations and reveals the origin of the cultivated gene pool, the genetic bottlenecks accompanying domestication, and the likely ecological and anthropogenic processes that shaped modern diversity and geographic patterning.

One of the cornerstones of both crop productivity and evolutionary biology is domestication genomics, the science of elucidating the often cryptic geographical origins and ecological contexts of our agronomically important plants (1–5). With roots tracing back to at least Charles Darwin’s 1868 seminal volume *The Variation of Animals and Plants under Domestication* (6), this global effort has been motivated by the need to understand the scope and patterning of genetic diversity in wild progenitor populations and the severity of the numerous, often sequential genetic bottlenecks that characterize initial domestication and subsequent crop improvement. In addition, identifying crop origins informs our understanding of wild gene pools that were “left behind” during human selection. These wild populations serve as important reservoirs of genetic variation, potentially harboring useful adaptations for crop improvement and natural stresses that were inadvertently removed during domestication (7–9).

Cotton (*Gossypium* L.) is one of the textbook examples of domestication and polyploid evolution in plants. About 1 to 2 Mya, a monophyletic allopolyploid lineage (2n= 26; 4*x*) originated from the combination of two ancestral diploids, i.e., an African-Asian A-genome (2n = 13; 2*x*) as the cytoplasm donor combined with the Central American D-genome (2n = 13; 2*x*) (10–12). This initial polyploid lineage gave rise to seven contemporary species, including the wild progenitors of the modern crop plants *Gossypium hirsutum* (Upland cotton) and *Gossypium barbadense* (Pima cotton). Archaeological records indicate that the independent, parallel domestication histories of *G. hirsutum* and *G. barbadense* started around 5,500 y ago in the northern Yucatán Peninsula and *c.* 8,000 y ago near Guayaquil in South America, respectively (13). These source plants were selected for fiber traits (“fibers” are dramatically elongated single-celled epidermal seed coat trichomes) as well as numerous other plant habit and architectural and physiological traits, resulting in the transformation of perennial wild plants producing short-brown fiber into annualized, high-yielding modern cultivars (12, 14–17). These cultivars provide the abundant, long, and strong white fibers that comprise the foundation of the global textile industry.

Over the millennia, the earliest domesticated forms of *G. hirsutum* spread locally and then regionally among numerous indigenous cultures across Central America and the Caribbean, even into northern South America. This dispersal was accompanied by diversification into a multiplicity of morphological forms, including perennial, multibranching shrubs to small trees, to annualized plants having variously abundant to sparse fiber, with this diversity reflecting selection as well as the intermingling of wild, feral, and cultivated gene pools across a wide swath of the subtropical Americas (13). Superimposed on this human-mediated ecogeographic spread were natural forces such as hurricanes and floods that further contributed to a continually changing landscape of diversity and distribution of local populations (16). Before Columbian colonization, *G. hirsutum* had already achieved an aggregate geographic range encompassing Central America to northern South America, including numerous Caribbean islands and Florida, whereas *G. barbadense* was primarily distributed in northern South America to the Lesser Antilles of the Caribbean (12, 15). This geographical overlap between the two species led to unintentional as well as intentional reciprocal genetic introgression resulting in domesticated gene pools (cultivars and earlier domesticated gene pools) that show admixture signals from both species (18–20). Following European colonization, further refinement of the modern, highly annualized cultivars included additional episodes of interspecific gene flow between *G. barbadense* and *G. hirsutum*.

Given its economic importance, there has long been interest in the origin and diversification of *G. hirsutum* as a crop plant (17, 21, 22). Early studies were based on plant distribution, morphological traits, and to a certain extent archaeological records (15, 23), with later investigations using more contemporary tools, including those of molecular systematics (11, 14, 24), and more recently whole genome and pangenome comparisons (19, 20, 25). From this and earlier work a proposal emerged that the northern Yucatán Peninsula was the locus of original domestication (12, 14, 19), noting, however, that this inference was based on sparse sampling of truly wild populations. For this reason, and because of the compounding challenges raised by interspecific introgression and escape of cultigens into natural settings, quantifying and characterizing purely *wild* diversity has proved challenging (16, 26).

**Fig. 1.**
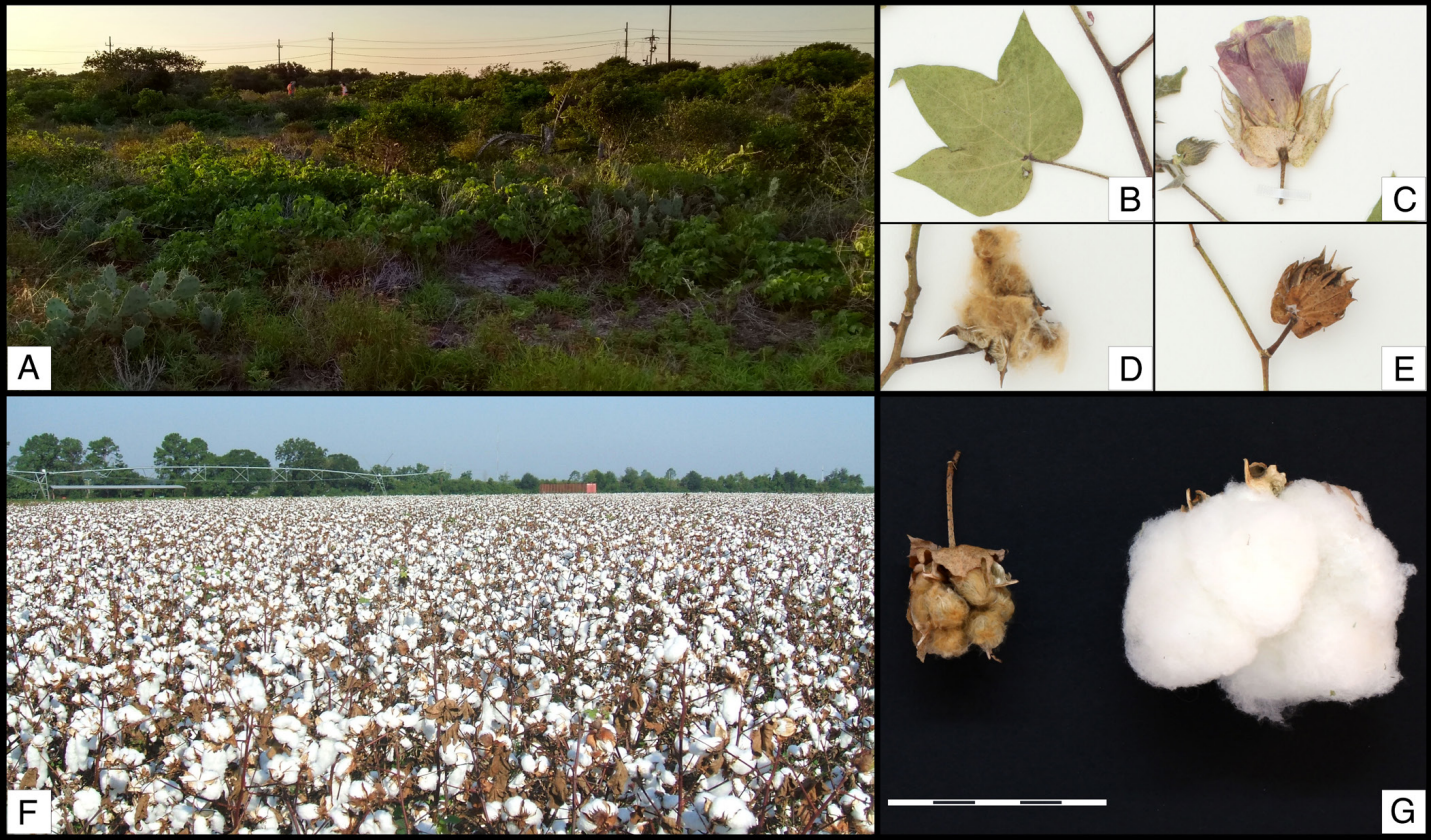
Morphology and habitat of wild *G. hirsutum* from the Yucatán Peninsula (México), in comparison to modern cultivars. (*A*) Natural coastal and inland Yucatán habitats. (*B*) Close-up view of a leaf (ISC accession 457839). (*C*) Close-up view of a flower (ISC accession 457839). Pink-to-purple petal coloration is common as flower senesces. (*D*) Open capsule showing the short, brownish fiber (ISC accession 457836). (*E*) Closed boll showing subtending bracts (ISC accession 457837). (*F*) Cultivated cotton field (Begonia, CC BY-SA 3.0, via Wikimedia Commons). (*G*) Size comparison between wild (*Left*) and domesticated (*Right*) cotton capsules. The scale bar represents 1 cm.

Three-quarters of a century ago, infraspecific variation in *G. hirsutum* was conceptualized as comprising a number of “races” (23), which represented geographical-genetic clusters of mostly primitively domesticated perennial cottons that were variably heterogeneous. In addition to these cultivated “races,” Hutchinson proposed race “Yucatanense,” to include the cotton populations distributed in the northern Yucatán Peninsula. These sprawling multibranched shrubs have smaller leaves, flowers, and capsules than cultivated forms and predominantly short brown fibers when compared to modern cultivars (Fig. 1 and *SI Appendix*, Fig. S1). In the northern part of Yucatán (México), they often form large but sporadically distributed populations. In addition, “Yucatanense” populations have been reported across the Caribbean basin, including numerous sites in Florida south of Tampa Bay into the Florida Keys, and the leeward sides of many Caribbean islands (12, 15–17, 27). These populations often inhabit arid coastal environments, often in relatively isolated coastal scrublands. Recent analysis using whole genome sequence data has shown that these populations are low in genetic diversity and likely represent relictual isolates derived from a once more-contiguous metapopulation system (16, 28).

Wild *G. hirsutum* comprises the foundational resource for the world’s most important fiber crop, yet little is understood about the amount and distribution of genetic diversity in wild cottons. Here, we systematically sampled and sequenced newly collected wild cottons, including 158 plants from the northern Yucatán Peninsula and 141 additional plants from southwestern coastal Florida. These data were incorporated into a previously generated phylogenomic framework of mostly cultivated or feral *G. hirsutum* (19) and a smaller sampling from several Caribbean islands (16, 28). Our goals were to address the following questions: 1) What are the genetic relationships within and between domesticated and wild cottons? 2) Do genomic data support northern Yucatán as the domestication center of *G. hirsutum*? 3) How much diversity exists in wild *G. hirsutum,* and how is it distributed within and among populations? 4) What are the evolutionary genomic consequences of population fragmentation? 5) Do genomic comparisons identify domestication signals associated with the transition from wild cotton to modern cultivars?

## Results

### TX2094 Reference Genome Assembly.

To maximize resequencing mapping quality, we generated a high-quality de novo reference genome assembly for the Yucatán accession TX2094 of *G. hirsutum* (NCBI: PRJNA1045685; *SI Appendix*, Fig. S2), which was used for all comparative genomic analyses reported here. In brief, PacBio sequencing data were assembled into 3,035 contigs, which were scaffolded using Hi-C into 26 pseudochromosomes (2.30 Gb total), yielding a scaffold N50 of 108.0 Mb and a maximum scaffold length of 127.8 Mb. BUSCO (Benchmarking Universal Single-Copy Orthologs) (29) analysis suggested 99.3% completeness with 97.1% being duplicated, as expected for a polyploid.

### Relationships within Wild *G. hirsutum*.

**Fig. 2.**
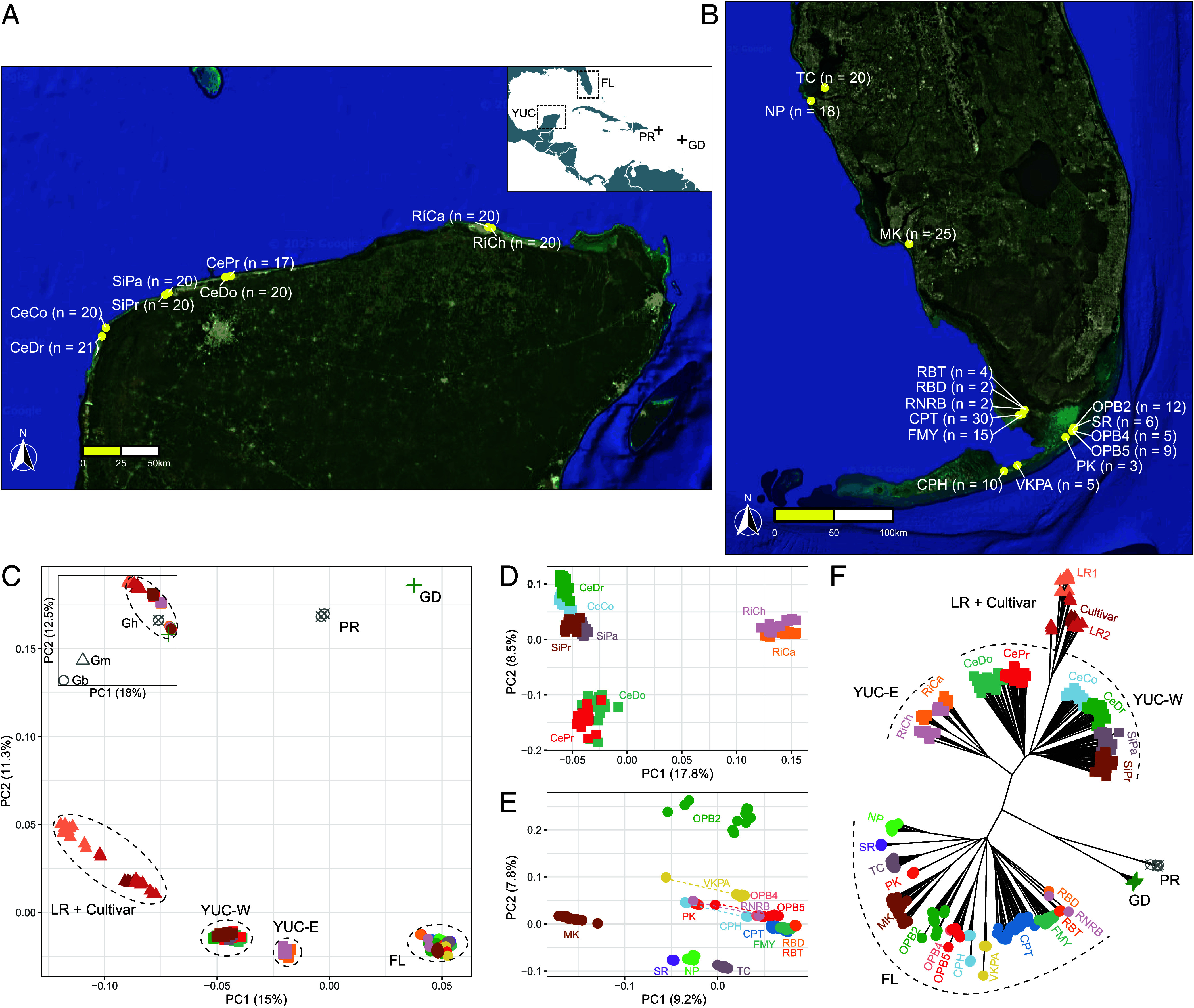
Field sampling and major genetic groups within wild *G. hirsutum*. Sample distributions are shown for (*A*) 158 individuals across four pairs of sites spanning the northern Yucatán (YUC) Peninsula, Mexico, from west (YUC-W) to east (YUC-E), and (*B*) 166 individuals across 15 sites in south- and north-western Florida (FL), USA. Distributions for Puerto Rico (PR) and Guadeloupe (GD) are *Inset* in the *Upper* *Right* corner of (*A*) and are as per (28). PCA results of five genetic groups in *G. hirsutum* (Gh) are shown in (*C*). Each genetic group is represented by a unique symbol, where colors indicate different sites or groups. PCA including the outgroups *G. barbadense* (Gb) and *G. mustelinum* (Gm) is *Inset* in the *Upper* *Left* corner of (*C*). (*D* and *E*) PCA results for Yucatán and Florida cottons, respectively, with each sample represented by a colored shape; samples from the same site share the same color. Outlier samples are connected to the major group by dashed lines (*E*). A neighbor-joining tree of *G. hirsutum* (Gh) populations is shown in (*F*).

Phenotypically wild cotton plants (*G. hirsutum*) were sampled from Florida (n = 141 across 15 sites) and Yucatán (n = 158 across four pairs of sites; Fig. 2 *A* and *B*). Many populations in Florida were small, consisting of at most several dozen plants, with a few being more extensive, particularly in Everglades National Park and in Tavernier Key. A more extensive population system is found in the coastal shrubland of northern Yucatán, with many areas containing hundreds of plants. On average, the sequencing depth for these newly collected cottons was similar, 22× in Florida and 27× in Yucatán (*SI Appendix*, Table S2).

Because feral *G. hirsutum* can evolve phenotypically wild characteristics once reestablished in nature (17), we compared sequence data for these newly collected samples to existing resequencing data from 1) previously sequenced domesticated cottons (*SI Appendix*, Table S3) (19), 2) three previously described wild *G. hirsutum* populations (n = 51) (16, 28), and 3) two congeners, wild *G. barbadense* (n = 9) (28) and *G. mustelinum* (n = 3), to serve as outgroups. Among the domesticated cottons, we included both modern cultivars (Cultivar, n = 10), as well as two phylogeographically defined clusters of accessions from the Caribbean (loosely termed Landrace1, n = 10) and Central America (Landrace2, n = 10) that each include perennials, early domesticates, and feral cottons (19). As previously noted (28), although feral cottons may regain wild characteristics, they retain identifiable genomic signatures of domestication. Here, we confirmed that all 299 newly collected samples represent wild individuals (Fig. 2*C*) prior to downstream analyses.

Across these 392 samples, 21.6 million (M) biallelic variants passed filtering for missing data and minor allele frequency (*Materials and Methods* and *SI Appendix*). From these, we retained 2.4 M linkage disequilibrium (LD) pruned biallelic SNPs for our initial analyses. Principal component analysis (PCA; *Upper* *Left* box in Fig. 2*C*) using the initial 2.4 M SNPs separated all *G. hirsutum* samples (dashed circle) from the outgroups *G. barbadense* and *G. mustelinum*. Accordingly, we removed the outgroup samples and refiltered for missing data, minor allele frequency, and linkage, yielding 1.9 M SNPs across 380 *G. hirsutum* samples. The PCA of *G. hirsutum* largely divided samples by geographic origins and domestication status, particularly along PC1, which explained 15% of the variation (Fig. 2*C*). Yucatán (YUC) samples were positioned in between the domesticated cottons [i.e., Cultivar, Landrace1 (LR1), and Landrace2 (LR2)] and the remaining wild cottons. Whereas wild cotton populations from the Caribbean (i.e., Florida, Puerto Rico, and Guadeloupe) all formed clusters for each location, the Yucatán cottons exhibited two distinct clusters separating the northeastern (i.e., YUC-E; RiCh/RiCa in Fig. 2*A*) from the northwestern (YUC-W) populations. These are part of a larger population system that varies in contiguity due to (sometimes large) swaths of mangroves and human landscape modification, both of which isolate the YUC-E populations from the YUC-W populations. Additional separation among samples was seen along PC2 (11.3% variance), which primarily separated the Caribbean island cottons from the larger mainland populations. In addition, two region-specific PCAs indicated highly geographically correlated genetic clusters in the Yucatán region; in contrast, Florida cotton exhibited no clear correlation with geography (Fig. 2 *D* and *E*).

Intersample relationships were also phylogenetically surveyed using *G. mustelinum* as an outgroup. Genetic distances among all 392 samples were computed using the initial 2.4 M SNPs, which served as input for neighbor-joining tree reconstruction (*SI Appendix*, Fig. S3). As expected, the topology recovered *G. barbadense* and *G. hirsutum* as sister groups exhibiting intraspecific monophyly. Within *G. hirsutum*, the tree topology largely recovered the same groups evident on the PCA. The initial split separated domesticated cottons from their wild-collected counterparts, forming two sister groups: one with the 30 individuals representing all domesticated gene pools and the other with all previously identified wild cotton populations along with the newly added (n = 310) individuals from Florida and Yucatán. As expected from previous work (19), modern cultivars were sister to the paraphyletic Landrace2 cottons. Among wild *G. hirsutum*, the northwestern Yucatán cottons (YUC-W) formed a clade sister to all remaining wild cottons, including the northeastern Yucatán cottons (YUC-E) from Rio Largatos sites Chorlitos (RiCh) and Cangrejos (RiCa). The YUC-E cottons were resolved as sister to all Caribbean cottons, including the large clade of Florida cottons (*SI Appendix*, Fig. S3). These results were largely recapitulated in an unrooted neighbor-joining tree constructed from the 1.9 M SNPs characterizing *G. hirsutum* alone (n = 380), the only difference being, perhaps significantly given previous indications of the origin of the initial domestication (12, 14, 19), that the domesticated cotton clade was nested within the northwestern Yucatán cotton (YUC-W) clade (Fig. 2*F*).

Genetic structure analysis (*SI Appendix*, Fig. S3) using LEA (30) for all 392 samples recovered at least 28 ancestral populations (K = 28 by cross-entropy; *SI Appendix*, Fig. S4), including one population each for *G. barbadense* and *G. mustelinum*. Among the 26 ancestral *G. hirsutum* populations, genetic structure analysis recovered two ancestral populations for cultivated cottons and 24 populations for wild cottons. The cultivated gene pool was generally distinct from all wild populations, except for a few landraces, which may indicate incomplete lineage sorting, or more probably the presence of introgressed genome fragments from *G. barbadense* (discussed below), which are known to exist within modern cotton cultivars (12, 19, 20). Notably, some cultivars and the Landrace2 samples contained a small proportion of ancestry that was primarily found in the western Yucatán populations (population number: P6, P7, P20; *SI Appendix*, Fig. S3). Among the wild cottons, populations largely recapitulated geographic sampling, albeit with some evidence of admixture among geographically proximal populations. These include samples from the northwestern Yucatán (YUC-E; Fig. 2*C*), whose dominant ancestry is frequently site-specific but with minor shared allelic composition with other YUC-W sites. Likewise, sites from southwestern Florida (i.e., RBD, RNRB, FMY, CPT, OPB2, and PK) often exhibited site-specific ancestry with additional contributions from other populations (*SI Appendix*, Fig. S3). Genetic structure among Florida populations combined with a lack of correlation with geography collectively (Fig. 2*E* and *SI Appendix*, Fig. S3) suggest that Florida harbors genetically distinct populations, composed of isolated demes that over time are capable of long-distance admixture.

### Evidence of Relationships from Whole Plastome Data.

**Fig. 3.**
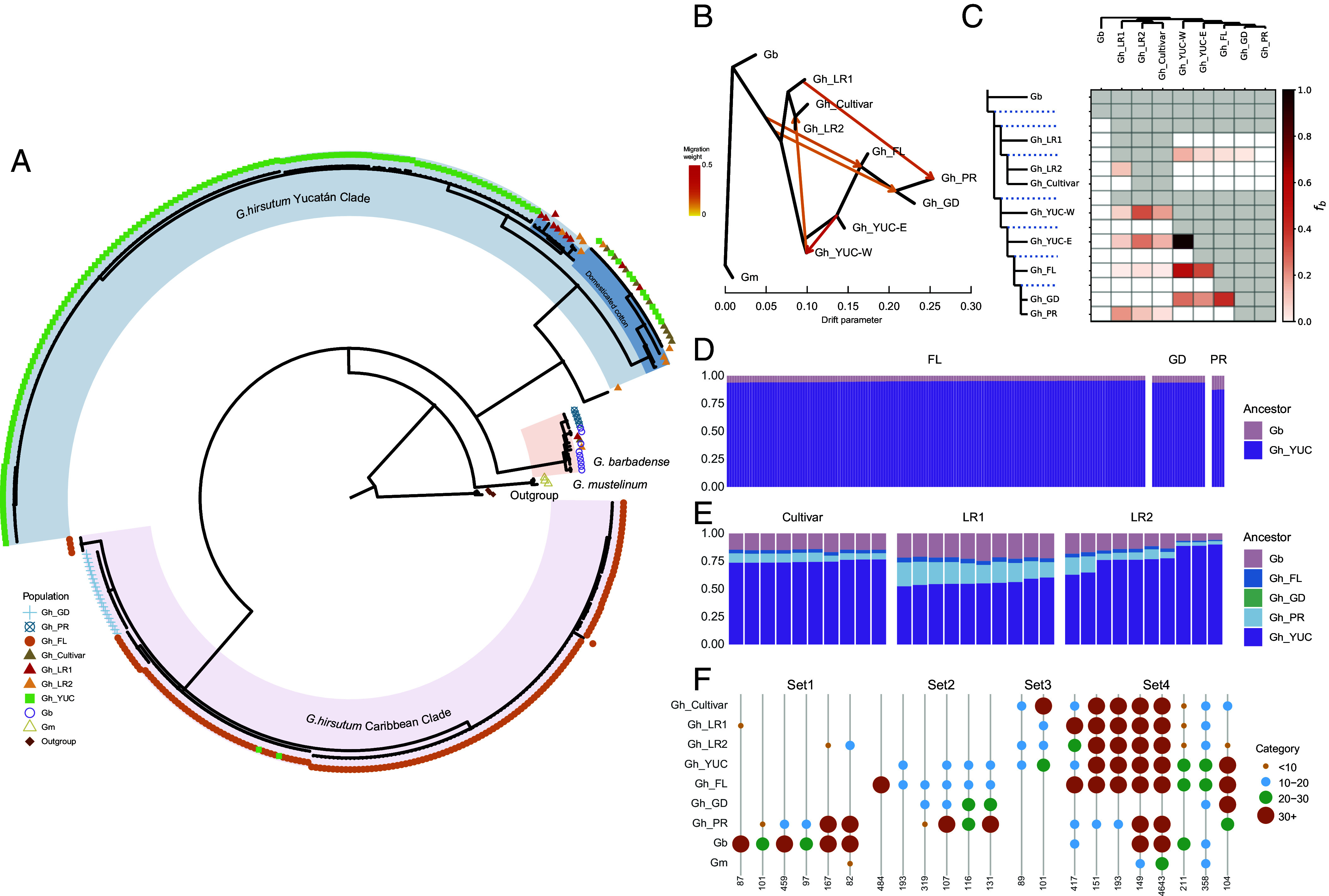
Inference of genetic relationships among *G. hirsutum* populations and *G. barbadense*. (*A*) Phylogenetic tree based on whole plastome sequences for 395 samples. Genetic group of each sample is indicated by color at the tips, and major clades are highlighted with colored shading. (*B*) TreeMix results with five estimated migration edges, represented by colored arrows. (*C*) Introgression signals detected by Dsuite are represented using the F-branch (*f*b; 0-1) statistic, with darker colors indicating stronger signals. (*D* and *E*) Local ancestry inference results for wild and domesticated cottons, respectively. Reference ancestries are shown on the right side of the plots, and each candidate group is represented by stacked bars colored according to the proportion of each ancestry. (*F*) Overlapping patterns of kmers detected in each group using KmerCity. The frequency (number of occurrences) of each kmer is represented by colored dots of different sizes, and the number of kmers sharing the same overlapping pattern is indicated below each plot.

Whole plastomes were reconstructed for the 392 sequenced *G. hirsutum* samples and aligned with three previously published (diploid) *Gossypium herbaceum* plastomes (31) as outgroups. The final alignment contained 159,556 sites, including 377 maximum parsimony informative sites, which were used in a maximum likelihood inference. After collapsing nodes with bootstrap values less than 80, the resulting topology suggested an unresolved polytomy relationship between two *G. hirsutum* clades (i.e., the Caribbean Clade and Yucatán Clade) and *G. barbadense* (Fig. 3*A*). The Caribbean Clade was generally geographically consistent with all wild cotton samples from Florida and Guadeloupe. In contrast, nearly all Yucatán cottons formed a distinct plastome type (Yucatán Clade), with the exception of two Yucatán samples (YUC_CeDO_M3 and YUC_CePr_M6; *SI Appendix*, Fig. S5) nested within the Florida cottons. Importantly, most of the domesticated cottons were nested within the Yucatán cottons, except three that were nested within *G. barbadense*, likely resulting from introgression during domestication and improvement. As expected from previous results (28), the plastomes from five wild Puerto Rico samples were also all nested within *G. barbadense*. Taken together, the plastome data support the hypothesis for a single Yucatán origin of domestication, and interspecific introgression with *G. barbadense*.

### Interpopulation Gene Flow Using Nucleotide Data.

In addition to the evidence of intergroup gene flow from whole plastome data, we evaluated interpopulation relationships using genome data to detect signals of gene flow. TreeMix (32), which models population relationships based on allele frequency while incorporating inferences of historical gene flow, supported a model with five migration edges (m = 5) as the best fit (*SI Appendix*, Fig. S6 and Fig. 3*B*). Unsurprisingly, TreeMix inferred gene flow from the branch leading to all *G. hirsutum* into the node ancestral to all Caribbean cottons (i.e., Florida, Puerto Rico, and Guadeloupe), supporting early divergence of the Caribbean cottons from a broader ancestral gene pool. Additionally, gene flow was inferred from Landrace1 into the Puerto Rican cottons, which was demonstrated previously (28). Within the Yucatán populations, the northwestern populations (YUC-W) were inferred recipients of gene flow from the northeastern populations (YUC-E); however, this likely reflects an analytical artifact, as TreeMix does not account for scenarios such as a small founder population. The YUC-W cottons were also inferred contributors to the ancestral lineage of the Landrace2 and Cultivar groups, congruent with Yucatán as the domestication center. Results from Dsuite (33) recapitulated the TreeMix analyses (Fig. 3*C*), noting strong introgression signal (F-branch values; *f*b = 0.4 to 1.0) between the two Yucatán populations, from Yucatán to Florida cottons, and from Florida to the Guadeloupe population. In addition, mild introgression signals (*f*b = 0.2 to 0.3) were detected between the Landrace2 and Yucatán populations, as well as between Landrace1 and the Puerto Rican cottons. Together, these results generally support these geographically distinct populations, while providing insight into ancestry and limited gene flow among populations. Additionally, both support the close relationship between the domesticated gene pool (i.e., Landrace2) and the Yucatán populations, as hypothesized previously.

Although neither method detected signals of introgression from *G. barbadense* into *G. hirsutum*, both natural and intentional (i.e., during breeding) introgression has been previously described (18–20). Therefore, we specifically evaluated the extent of introgression from *G. barbadense* into the three Caribbean populations, as the two species became sympatric in the southern Caribbean subsequent to their independent, strongly allopatric independent domestications. Consistent with the foregoing analyses, neither *f*3 nor *f*4 analyses (*SI Appendix*, Fig. S7) using Admixtools2 (34) revealed signatures of introgression, which may result from the limited sensitivity of genome-wide statistics to detect low-level introgression that is diluted across the genomic background.

Local ancestry inference via FLARE (35) was used to estimate genomic proportion derived from the *G. hirsutum* domestication center (i.e., all Yucatán cottons; YUC-E and YUC-W) vs. wild *G. barbadense*; here, the Yucatán cottons are used to represent ancestral, pure *G. hirsutum*. Unsurprisingly, these results suggest minimal *G. barbadense* ancestry in the nonoverlapping Florida cottons; however, a similar proportion was found in the southern Caribbean Guadeloupe populations. Ancestry in both sets of populations was estimated as 93 to 95% from Yucatán cotton and only around 5% originating from *G. barbadense* (Fig. 3*D*), the latter possibly suggesting shared ancestral polymorphisms between the two species. In contrast, the Puerto Rican population exhibited a higher proportion of *G. barbadense* ancestry (12.5%). This is perhaps unsurprising, given the known introgression from domesticated cotton into the Puerto Rican populations (Fig. 3*A*). We next used all populations (including the Puerto Rican cottons) as potential genomic sources to evaluate the ancestry of the three cultivated gene pools. Both Cultivar and Landrace2 groups showed similar proportions of Yucatán ancestry (74 to 76%), with 13 to 14% originating from *G. barbadense* (Fig. 3*E*). In contrast, Landrace1 contained only about 55% Yucatán ancestry and a higher proportion (22%) of *G. barbadense*. Although all domesticated cottons shared low amounts of Florida ancestry (2 to 4%), this likely is due to shared ancestral variation rather than gene flow.

We also used a kmer approach to evaluate intergroup gene flow. KmerCity (36) tabulated overlapping patterns of 50 bp kmers extracted from genomic repetitive regions to detect putative introgression segments from *G. barbadense* into *G. hirsutum* (*SI Appendix*, Table S4). Among 158 representative individuals (*Materials and Methods*), kmer signatures of *G. barbadense* introgression into *G. hirsutum* were limited, although the data recapitulated the observed introgression present in the Puerto Rican and Landrace1 cottons. Only 87 kmers were found uniformly in *G. barbadense* and at low frequency in Landrace1 (Set1 in Fig. 3*F*); 346 kmers were present only in *G. barbadense* and at low frequency in Puerto Rican cotton; and 249 kmers were shared among *G. barbadense*, Landrace2, and Puerto Rican cottons at variable frequencies. Among the remaining kmers was a set of 1,350 (Set2, Fig. 3*F*) present only in wild *G. hirsutum*, including 484 kmers unique to the Florida populations. Notably, a set of 190 kmers (Set3, Fig. 3*F*) was found only in domesticated and Yucatán cottons at variable frequencies, underscoring the close relationship between the Yucatán populations and the domesticated gene pools. All remaining patterns (Set4, Fig. 3*F*) showed a high number of missing kmers in Guadeloupe cotton (total 5,764 kmers), possibly reflecting its severe bottleneck history.

### Genetic Diversity and Divergence within and among Wild Cotton Populations.

**Fig. 4.**
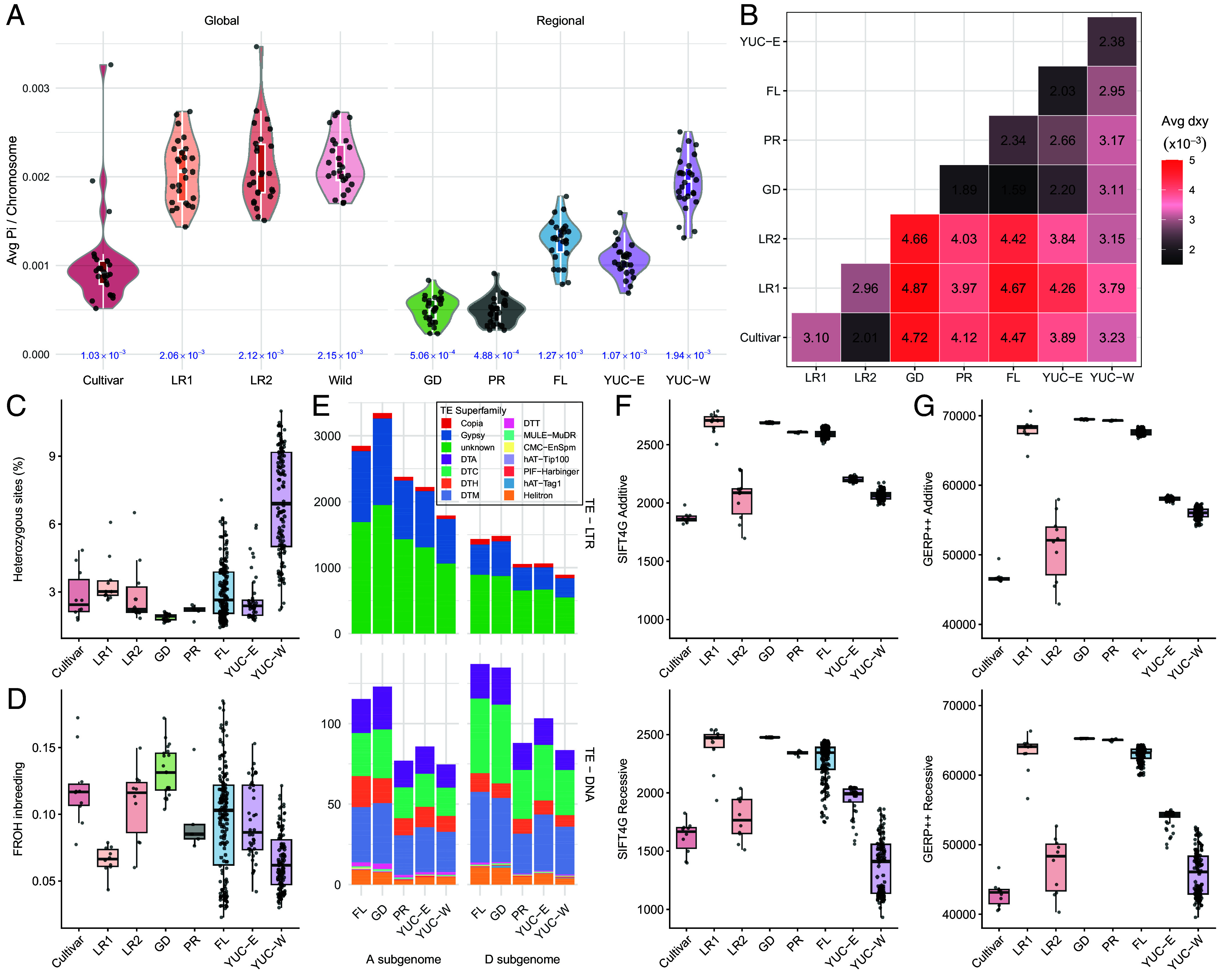
Genetic variation, transposable element insertion polymorphisms (TIPs), and genetic load in wild cotton populations. (*A*) Average nucleotide diversity (π) for each group; each point represents the mean π for an individual chromosome. (*B*) Pairwise nucleotide sequence divergence (d_XY_) among groups. (*C*) Proportion of the observed heterozygous sites (*He*), and (*D*) the proportion of the genome occurring in runs of homozygosity (F_ROH_), respectively. Each black dot represents an individual. (*E*) Average TIPs per individual across 116 representative samples, divided into two major classes: LTR and DNA. Bars are filled by TE superfamily as annotated in the TE library. (*F* and *G*) GERP++ and SIFT4G genetic load per population, with each individual represented by a black dot.

Global nucleotide diversity (π; Fig. 4*A*) comparisons showed that modern cultivars (1.03 × 10^−3^) exhibited roughly half of the variation observed in the two landrace groups (Landrace1: 2.06 × 10^−3^; Landrace2: 2.12 × 10^−3^) and all wild cottons (2.15 × 10^−3^), indicative of the strong genetic bottleneck accompanying domestication and crop improvement. These estimates suggest that, on average, only 1 nucleotide per kilobase differs between any two cultivars, underscoring the severe loss of diversity accompanying modern crop development. In contrast to the dearth of diversity in modern cultivars, wild cotton populations were generally more diverse, with the highest π being in the western Yucatán population (YUC-W; 1.94 × 10^−3^), followed by Florida (1.27 × 10^−3^) and northeastern Yucatán cottons (YUC-E; 1.07 × 10^−3^). To the extent that the “center of diversity represents the center of domestication” (12, 14), these data further point to the Yucatán as the site of initial domestication. Moreover, these data suggest that two individuals randomly sampled from the northwestern Yucatán exhibit, on average, nearly twice the nucleotide divergence than any two modern cultivars. In contrast to these more diverse populations, those from the Caribbean islands of Guadeloupe (5.06 × 10^−4^) and Puerto Rico (4.88 × 10^−4^) exhibited only about one-quarter of the diversity observed in northwestern Yucatán cotton and about half of that found in the cultivars. We interpret these data as reflecting the population level consequences of bottlenecks associated with dispersal, inbreeding, and small census sizes.

To quantify divergence among these various population groupings and gene pools, we calculated pairwise nucleotide sequence divergence (d_XY_; Fig. 4*B*). These data show slightly greater divergence between wild and domesticated cottons (range 3.15 × 10^−3^ to 4.87 × 10^−3^) relative to comparisons among wild populations (1.59 × 10^−3^ to 3.17 × 10^−3^) or domesticated groups (2.01 × 10^−3^ to 3.10 × 10^−3^). Notably, the northwestern Yucatán population exhibited lower sequence divergence from domesticated cottons and higher divergence from other wild populations. Together, these results demonstrate the reduction in genetic diversity (π) associated with domestication and support a close evolutionary relationship between the northwestern Yucatán populations and the domesticated cottons, further supporting a Yucatán-centered domestication.

Because genetic bottlenecks leave genomic signatures, we evaluated each sample for 1) intraindividual observed heterozygosity (*He*), 2) inbreeding coefficient (F_IS_), and 3) extended runs of homozygosity (ROH), the last providing a measure of identity-by-descent, where length reflects the timing and severity of population bottlenecks (37). Among domesticated cottons, all individuals exhibited similar estimated *He* values (2.9 to 3.5%) and inbreeding coefficients (F_IS_ = 0.738 to 0.784), generally suggesting similarly small effective population sizes for much of their history, as expected. Consistent with stronger recent selection in the cultivars, the proportion of the genome contained within runs of homozygosity (F_ROH_) was nearly twice as high in cultivars (0.12) and Landrace2 (0.108) as in Landrace1 (0.066). Because longer ROH are associated with more recent inbreeding among closely related individuals, these patterns suggest more recent inbreeding or stronger bottlenecks among the cultivars and Landrace2 (*SI Appendix*, Fig. S8). Among wild cottons, the northwestern Yucatán population showed the highest proportion of observed heterozygous sites per individual (He = 7%), twofold to threefold higher than in other wild populations (1.9% in Guadeloupe to 3.0% in Florida; Fig. 4*C*). Likewise, the northwestern Yucatán populations also have the lowest level of inbreeding (Fig. 4*D*), as indicated by a lower inbreeding coefficient (F_IS_ = 0.471) and a smaller proportion of the genome contained within long ROH (F_ROH_ = 0.065). Although all remaining wild cottons showed similar F_IS_ values (0.771 in Florida to 0.857 in Guadeloupe), the population from Guadeloupe (0.132) exhibited 1.4 fold higher F_ROH_ than other three populations, consistent with more recent or severe inbreeding (28). Overall, these results provide additional support for the northwestern Yucatán population system as the center of diversity (i.e., most diverse), and that it is relatively panmictic compared to other wild cotton populations. Also, the wide range in *He* and F_ROH_ observed among samples from both Florida and Yucatán indicates the presence of fine-scale genetic structure within each region, likely driven by local inbreeding (for more information, see *SI Appendix*, Figs. S9–S11).

### Efficiency of Natural Selection among Wild Cotton Populations.

To investigate the genomic consequences of population fragmentation, we compared the average number of TE insertion polymorphisms (TIPs) per sample (Fig. 4*E*). The number of TIPs detected in each group was approximately 18 times higher for long terminal repeat (LTR) TEs than for DNA TEs, consistent with expectations based on a recent TIP survey in allopolyploid cotton and the diploid ancestors (38). In all groups, subgenome A contained roughly twice the number of LTR-TIPs as subgenome D, reflecting the differences in genome size among diploid cotton ancestors (38, 39). In contrast, DNA-TIPs were slightly (1.2 times) more abundant in the D genome than the A genome. Among sites, wild cotton populations outside of had higher TIP counts for both TE classes, especially the Florida and Guadeloupe populations, which averaged *ca.*1.4 times higher TIP frequency than in northwestern Yucatán cottons. This pattern may reflect historical population bottlenecks combined with less efficient selection for removing deleterious TEs (40, 41).

We further explored genetic burden using two complementary approaches targeting the coding regions of the genome: SIFT4G (42) and GERP++ (43). Comparisons of genetic load indicate that the geographically more isolated wild cotton populations exhibit significantly higher load than the northwestern Yucatán cottons in both additive and recessive models of SIFT4G and GERP++ (Fig. 4 *F* and *G* and *SI Appendix*, Table S6). Among domesticated cotton groups, cultivars generally exhibited significantly lower genetic load than the northwestern Yucatán cottons, perhaps reflecting strong selection for yield and fitness under crop improvement. Among the early domesticates, Landrace1 exhibited load comparable to the other Caribbean cottons, whereas Landrace2 showed no significant difference relative to northwestern Yucatán cotton in the SIFT4G additive model and the GERP++ recessive model.

### Selection under Domestication.

Unlike many other crop plants (e.g., ref. 1), to date no “domestication genes” have been discovered in cotton, notwithstanding the dramatic morphological differences between wild and domesticated plants (Fig. 1). Because high levels of population substructuring and linkage disequilibrium, together with low levels of diversity and effective population size, can constrain the power of inference of selection (44), our ability to detect selection under domestication may be limited. Nonetheless, to explore potential targets of selection, we compared the 118 northwestern Yucatán wild cottons to the 109 cultivar accessions (*SI Appendix*, Fig. S12 *A* and *B*) using three complementary metrics: 1) the ratio of nucleotide diversity between wild and domesticated gene pools (π_YUC-W_/π_Cultivar_), 2) their between-group Fst, and 3) their allele-frequency differentiation (via XP-CLR) (*SI Appendix*, Fig. S13). We conservatively defined domestication regions as genomic intervals that were within the top 5% of values for at least two of the three methods. Across the genome, 6.10% of regions were identified as domestication candidates by at least two of the three statistics, while only 0.05% of the genome was supported by all three (*SI Appendix*, Fig. S12*C*). Domestication signals were unevenly distributed across chromosomes, with the largest proportions on chromosome A12 (0.93% of total genome) and D10 (0.50%). In total, 3,562 genes (including paralogs and isoforms) were found within the “domestication regions” (*SI Appendix*, Table S8), of which only 2,502 protein sequences were identified using the Swiss-UniProt database. GO enrichment analysis of these loci in *G. hirsutum* showed a significant overrepresentation of genes in meristem maintenance (GO:0010073; FDR = 4.38 × 10^−4^), meristem development (GO:0048507; FDR = 4.26 × 10^−4^), and tissue development (GO:0009888; FDR = 2.20 × 10^−2^). Within the 0.05% of the genome surviving the statistical thresholds of the three selection tests, only 21 protein sequences were identified using the Swiss-UniProt database (*SI Appendix*, Table S8), including a hydrotropism-related gene (MIZU-KUSSEI1), which has been shown is important in cotton root growth (45). Collectively, our data suggest that domestication was a gradual process involving a long-term accumulation of quantitatively minor mutations (46, 47), as opposed to a saltational process involving “major” domestication genes.

## Discussion

Identifying the origin of domesticated gene pools has been a central goal in understanding crop evolution (48, 49). Wild populations provide a critical foundation for understanding genomic evolution under domestication (3). Population genomic studies in crops such as maize (50), rice (51), sunflower (52), and wheat (53) have demonstrated how wild populations can illuminate the demographic and adaptive evolutionary processes underlying domestication. In species such as rice, sunflower, and wheat, for example, analyses of wild relatives have revealed the importance of historical gene flow between the domesticated gene pool and their wild relatives in conferring phenotypic traits and bolstering genetic diversity (51–53). In maize, characterizing variation among teosintes has identified alleles with adaptive potential for both flowering-time pathways and environmental tolerance (50). With respect to *G. hirsutum*, and notwithstanding the long interest in the species from the scientific community, relatively little is understood about levels and patterns of genetic variation in nature. This is particularly true for the scattered surviving pockets of populations that arguably are actually wild, as opposed to being derived from feral progenitors that escaped back into natural settings during the many millennia of cultivation subsequent to domestication. Accordingly, our understanding of diversity in wild cottons has largely been restricted to resequencing data from small samples of historically collected material from germplasm banks (19). Recent efforts in collecting wild *G. hirsutum* in the Caribbean and Florida (16, 28) have revealed the genomic consequences of inbreeding and founder events in these small populations, suggesting that they likely represent peripheral, ecogeographically isolated lineages. Historical records note the presence of larger populations especially in the Yucatán peninsula, which over 30 y ago was inferred as the center of domestication based on allozyme and RFLP markers (12, 14). Following our collective recent sampling efforts, we furnish here a broader perspective on diversity and domestication in *G. hirsutum* by extending our previously established framework (19) to include multiple samples from numerous wild populations covering the northern Yucatán peninsula.

Genome resequencing of wild and feral cotton populations from throughout the Caribbean basin, including Yucatán, provides the context for evaluating the hypothesis of a Yucatán origin of the modern crop plant gene pool. Modern cultivars are closest to the northwestern Yucatán samples in multidimensional space (Fig. 2*C*), although not fully embedded within those samples (discussed below). This indication of a Yucatán origin of the modern crop gene pool is additionally supported by phylogenetic analysis and genetic distance metrics (Fig. 2*C* and *SI Appendix*, Fig. S3). Finally, the plastome data show that domesticated cottons as a group mostly are nested within the Yucatán cottons, with the exception of several accessions that contain *G. barbadense* plastomes (Fig. 3*A*), likely resulting from introgression during domestication and improvement. Collectively, the large comparative framework presented here demonstrates a single Yucatán origin of the gene pool from which the globally grown, modern annualized cotton crop plant was derived, and documents a center of wild diversity in the northwestern Yucatán.

This conclusion that northwestern Yucatán is the seat of domestication is further supported by recent chemical profiling of wild *G. hirsutum* populations (see ref. 54). Plants from 16 populations along the Yucatán northern coast were compared, showing a gradient from west-to-east for two different chemotypes. Most plants from the northeast populations (including RiCh/RiCa, sampled here) contained high concentrations of gamma-terpinene, a monoterpenoid absent from the profiles of domesticated cotton and from most plants in the northwestern Yucatán. Thus, variation in secondary chemistry provides an independent line of evidence supporting the western Yucatán as the cradle of cotton domestication.

An interpretation of a Yucatán origin leads to the prediction that genetic diversity would be highest among the wild source populations and become lower following sequential genetic bottlenecks accompanying geographic diffusion throughout the American subtropics and selection during crop improvement (reviewed in ref. 55). As shown here, nucleotide diversity is indeed highest in Yucatán (Fig. 4*A*), with more peripheral populations experiencing a loss of diversity at both the population and individual levels. Emblematic of this bottleneck, two individuals randomly sampled from the northwestern Yucatán have on average, twice the nucleotide difference than do any two modern cultivars. Also as predicted by the hypothesis of a Yucatán origin, estimates of individual heterozygosity are highest in the Yucatán (Fig. 4 *C* and *D*), and runs of homozygosity (ROH) are the lowest. These data all highlight the relatively high levels of genetic diversity that remain extant in the wild *G. hirsutum* populations of the northern Yucatán Peninsula. Finally, kmer analysis revealed 1,350 kmers present only in wild *G. hirsutum*, and 190 kmers (Set3, Fig. 3*F*) found only in cultivars and Yucatán cottons, additionally supporting the inferred single origin of the former from the latter.

The comprehensive genomic analysis of wild and cultivated cottons offers an excellent system for studying domestication and introgression, and also for understanding polyploid genome evolution shaped by isolation by geographic distance at the population level. As an example, consider transposable elements, the dominant component of most plant genomes, including cotton (20, 38). TE activity may be associated with population demographic changes (28) and TE presence or absence may be subject to selection. Here, we show that wild *G. hirsutum* populations have greater accumulation of TIPs in derived populations relative to the diversity center in the northwest Yucatán, with particularly high levels in Florida and Guadeloupe. We speculate that the reduced efficiency of purifying selection in smaller populations is linked to genetic bottlenecks, as indicated by reduced genetic diversity and accumulation of genetic load in northeastern Yucatán, Florida, and Guadeloupe (Fig. 4*A*). Indeed, the accumulation of deleterious mutations in edge populations is often attributable to their demographic history (56–58). In contrast, comparison of genetic load showed a lower burden in Landrace2 and Cultivars than in northwestern Yucatán populations, likely reflecting the purging of deleterious alleles during domestication (59).

Among the complications that have historically obscured phylogenetic understanding *G. hirsutum* domestication is the occurrence of introgression with *G. barbadense* (18, 20), which was independently domesticated in parallel in northwestern South America, but which became sympatric in many parts the Caribbean basin as both cotton species spread across the landscape in the ~5,000 y since initial domestication. Interspecific introgression among domesticated crops is increasingly recognized as a key source of adaptive variation in crop evolution (reviewed in refs. 60 and 61). Because bidirectional introgression has been documented for both species, here we characterized the phylogenomic extent of this introgression into *G. hirsutum*, showing that on average, approximately 13 to 14% of the genomes of modern cultivars were derived from the conspecific *G. barbadense* (Fig. 3*E*). This realization provides an explanation for why in multivariate depictions such as PCA (Fig. 2*C*), modern cultivars are adjacent to rather than embedded within wild cottons from the northwestern Yucatán, while simultaneously providing clues to possible targets of selection that might have favored introgressed segments.

To evaluate selection under domestication, we used three complementary approaches, the ratio of nucleotide diversity between wild and domesticated gene pools, between-group Fst estimates, and allele-frequency differentiation between wild plants and cultivars. Because several sources of false-positives are possible in these analyses, we used a conservative threshold for “possible selection,” requiring a positive signal for either two or all three of these approaches and stringent cutoffs. Although several thousand genes were included overall in putatively selected regions, no single region or gene stood out as a primary target of selection (*SI Appendix*, Table S7). These data are consistent with the conclusions of other recent reports (e.g., ref. 19). Our data, in conjunction with numerous QTL and GWAS studies that fail to reveal major domestication genes underlying fiber evolution (62–64), photoperiod control (65, 66), or other major morphological transitions accompanying domestication (47), support an interpretation that cotton domestication was a gradual process involving the long-term accumulation of mutations with relatively minor phenotypic effects. Similar polygenic selection has also been observed in other crops, such as threshability in wheat and shattering in rice (4, 67, 68), as opposed to a more punctuated process involving “major” domestication genes (e.g., tb1 in maize; 69).

## Materials and Methods

For full materials and methods, see *SI Appendix*.

We de novo assembled a reference genome for the “Yucatanense” wild cotton accession TX2094 maintained by USDA-GRIN using sequenced PacBio HiFi and Hi-C data. Transposable elements (TEs) and coding genes of TX2094 were annotated with EDTA (70) and BRAKER3 (71).

Population-level field sampling of wild *G. hirsutum* was conducted in Florida (n = 141; 14 sites) and Yucatán (n = 158; four site pairs), generating 150 bp paired-end sequencing data. Newly generated data were jointly analyzed with 1) 51 previously identified wild cotton individuals from Mound Key, Florida (n = 25) (16), Pitahaya, Puerto Rica (n = 5), and Guadeloupe (n = 21) (28); 2) 30 individuals representing three domesticated genetic pools – modern cultivars (Cultivar; n = 10) and early domesticated varieties (Landrace1 and Landrace2; LR1 and LR2; n = 10 each) (19); and 3) wild outgroups of *G. barbadense* (n = 9) and *G. mustelinum* (n = 3).

All raw reads were trimmed and mapped to the TX2094 reference genome. Genetic variant sites were identified across all samples and analyzed for genetic relationships using PCA, neighbor-joining trees, and population structure analyses. Interpopulation relationships were inferred using Treemix, Dsuite, Admixtools2, and FLARE. Plastome phylogeny was reconstructed using whole chloroplast genomes. We also calculated genetic diversity and inbreeding levels within each wild cotton population. Finally, TE content and genetic load were assessed and compared across wild populations. Domestication signal scans were performed by comparing wild cottons at the domestication center vs. modern cultivars for π, Fst, and XP-CLR. Code for all analyses are available at https://github.com/Wendellab/Wildcotton_YUCFL.

## Supplementary Material

Appendix 01 (PDF)

## Data Availability

Collection permits to J.F.W. at Florida Park Service 10312110 and Everglades National Park EVER-2022-SCI-0002. Collection permits to URJSR at Yucatán in reservation sites: SPARN/DGVS/03052/22 and out of reservation sites: SPARN/DGVS/02106/22. Import permit to J.F.W. for Yucatán samples shipped to Iowa State University 588-23-293-18606. Vouchers of Yucatán (Voucher ID: 457832 to 457865) and Florida cotton specimens (Voucher ID: 455876 to 456178) are deposited at Ada Hayden Herbarium (ISC) at ISU. All sequence data generated in this study are available from NCBI under PRJNA1231560 (Florida cotton) (72) and PRJNA1231062 (Yucatán cotton) (73). Sequence data for reference genome TX2094 are available from NCBI under JAXHOR000000000 (74). Final assembly of TX2094 deposited at NCBI Datasets under accession GCA_054990295.1 (75). Bioinformatic pipelines are available at Github: https://github.com/Wendellab/Wildcotton_YUCFL (76). All other data are included in the manuscript and/or *SI Appendix*.
