# Supplementary material for: Genomic diversity and the domestication history of cotton (Gossypium hirsutum)
Source: Proc Natl Acad Sci U S A. 2026 May 18;123(21):e2607107123. doi: 10.1073/pnas.2607107123 (PMC13213997; doi:10.1073/pnas.2607107123)
Supplement: Supplementary file 1 — Appendix 01 (PDF) [file pnas.2607107123.sapp.pdf]

PNAS

Supporting Information for
Genomic Diversity and the Domestication History of Cotton (*Gossypium hirsutum*)

Weixuan Ning, Mark A. Arick II, Joshua A. Udall, Chuan-Yu Hsu, Luis Abdala-Roberts, Uriel Solís-Rodríguez, Yeyson Briones-May, Zenaida V. Magbanua, Olga Pechanova, Carlos Bustos-Segura, Mary V. Clancy, Sandra Díaz-Cruz, Alejandra Garnica-Cabrera, Marine Mamin, John Z. Yu, Ted C. J. Turlings, Guanjing Hu, Daniel G. Peterson, Corrinne E. Grover, Jonathan F. Wendel

Corrinne E. Grover; Jonathan F. Wendel
Email: corrinne@iastate.edu; jfw@iastate.edu

This PDF file includes:

Supporting text
Figures S1 to S13
Tables S1 to S8
SI References

Supporting Information Text

Reference Genome Sequencing for TX2094

A *de novo* reference genome for “Yucatanense” cotton was constructed using an existing accession (TX2094; PI 501501) previously collected from the northwestern Yucatán Peninsula (México) and maintained by the USDA Germplasm Resources Information Network (GRIN; https://www.grin-global.org/). Plants were grown in a USDA greenhouse in College Station, TX. Fresh cotyledons from dark-grown seedlings were harvested using sterile, moist paper towels and used for high-molecular-weight genomic DNA isolation following the Qiagen MagAttract kit (Qiagen, Hilden, Germany). DNA shearing was done on a Megaruptor® 2 (Diagenode, Denville, NJ, USA) set to 20 kb and the fragment size was checked on a 5300 Fragment Analyzer (Agilent Technologies, Santa Clara, CA, USA). The genomic library was constructed following the PacBio SMRTbell Express Template Prep Kit 2.0 protocol. Library size selection was performed using a Sage Elf system, which runs a gel to separate DNA by size and then elutes sideways into 13 "bins". The top 5 bins were run on the Fragment Analyzer to select an appropriate size (usually in the range of 15-18kb).

To assist genome scaffold assembly, a Hi-C library for TX2094 was prepared using the Proximo Hi-C kit (Phase Genomics, Seattle, WA, USA). Library concentration and fragment size distribution were assessed using a Qubit Fluorometer (Thermo Fisher Scientific, Waltham, MA, USA) and an Agilent 4200 TapeStation system (Agilent Technologies, Santa Clara, CA, USA). The library was sequenced as 150 bp paired-end reads on an Illumina NovaSeq 6000 platform (Illumina, San Diego, CA, USA).

Genome Assembly for TX2094

A total of 1.6 million PacBio HiFi reads (average read length 14 kb) from five SMRT cells (PRJNA1045685; Table S1) were *de novo* assembled using Hifiasm v.0.19+ (1), generating a primary contig assembly that was subsequently curated prior to scaffolding. Hi-C libraries were aligned to the assembled contigs using BWA (2) mem (v0.7.17; parameters -5SP) with duplicate marking by SAMBLASTER (3) and filtering/sorting with SAMtools (4); contact pairs were extracted using matlock (https://github.com/phasegenomics/matlock) for downstream scaffolding. Hi-C contact links were sorted (LC_ALL=C sort -k2,2 -k6,6) and used as input to the 3D-DNA pipeline (5) to order and orient contigs into chromosome-scale scaffolds, producing AGP format and assembly structure files for 2 iterations of manual inspection and correction in Juicebox (6). Final scaffolds were exported into FASTA and AGP formats. The final assembly spans 2.43 Gb in contigs (GC content 34.82%), comprising 3,035 contigs with a contig N50 of 68.2 Mb and a maximum contig length of 221.8 Mb. Hi-C (91.6-fold coverage) scaffolding anchored and oriented 34 of these contigs into 26 chromosome-scale scaffolds (total length 2.30 Gb) with a scaffold N50 of 108.0 Mb and a maximum scaffold length of 127.8 Mb. The total length of each set of duplicated (homoeologous) pseudochromosomes was 1,435,390,769 and 853,683,938 bp for the A and D subgenomes, respectively (Fig. S2), reflecting their inherited genome size differences (7).

All TX2094 genome sequences were mapped to the diploid genome of the congener *G. longicalyx* (https://www.ncbi.nlm.nih.gov/datasets/genome/GCA_010883175.1/) (8) using minimap v2.26 (9) to orient chromosomes from both subgenomes to the same direction. A custom PERL script, along with agptools (10), oriented the sequences. Annotations were called using EDTA v2.2.2 (11) and BRAKER3 v3.0.7.6 (12) using two iterations. Initially, EDTA was used to call repeats without CDS sequences, producing a preliminary genome specific TE library. RepeatMasker v4.1.2 (13) masked the genome using this preliminary library. BRAKER3 produced a preliminary set of gene annotations using the masked genome and the Viridiplantae ODBv11 database (14). The second iteration used EDTA with the CDS sequences from the gene annotations along with the oriented genome sequences. BRAKER3 was run again using the Viridiplantae database and masked genome from EDTA.

The completeness of the assembled genome was assessed using BUSCO (Benchmarking Universal Single-Copy Orthologs) (15) with the eudicotyledons OBD12 database (16). Genome

annotation identified 85,371 predicted coding genes, with repetitive sequences accounting for 70.6% of the total genome (2.3 Gb). Among subgenomes, gene content was similar, with the D subgenome (43,651) containing a slightly higher number of predicted coding genes than the A subgenome (41,720). Repeat content was dissimilar between subgenomes, i.e., 41.1% of genome-wide repeat sequences were on the A chromosomes versus only 22.8% on D chromosomes, which reflects the known twofold difference in subgenome size (17). The final assembled genome TX2094 is deposited in NCBI (accession number JAXHOR000000000) and CottonGen. All bioinformatic scripts are deposited at: https://github.com/Wendellab/Wildcotton_YUCFL.

Population Sample Collection, DNA extraction, and Resequencing

In January 2022, JFW field-collected *G. hirsutum* samples with morphological features of wild cotton across Florida, along the southwestern coastline from just south of Tampa Bay to the southernmost tip of the Everglades. In total, 141 individuals were sampled from 14 sites (Fig. 2a). In addition, USR and YBM field-sampled 158 *G. hirsutum* exhibiting wild morphological features at four pairs of sites, as part of an extensive population system along the north coastline of the Yucatán peninsula. Each pair of sites is separated by 2 to 6 km, while the four sites are separated by 40 to 258 km (Fig. 2b). Within Yucatán, populations are found scattered throughout the coastline, but with a more continuous distribution and especially large numbers (from several dozen up to several hundred plants depending on the site) on the west side between the coastal towns of Celestún and Sisal. Populations are also substantial moving eastward along the coastline from the ports of Sisal to Telchac, after which the species' distribution becomes discontinuous approaching the port of Dzilam de Bravo (extensive flooding areas with mangrove vegetation reaching coastline), several dozen kilometers after which populations can be again found after the coastal towns of Río Lagartos and towards El Cuyo on the far east side of the state's coastline. Specifically, three of the sites are towards the northwestern end of the peninsula (i.e., CeDr/CeCo, SiPr/SiPa, CeDo/CePr; full description of the location code see Table S2); these three sites are collectively distant (~170 km) to the more northeastern RiCa/RiCh site (Fig. 2b). Sampling distribution maps (Fig 2a, 2b) were generated using the R package ggmap (18), with map data © 2026 Google.

Leaves from individual plants were separately collected and stored in small bags of silica gel prior to DNA extraction. The genomic DNA isolation and Illumina DNA-Seq library preparation were conducted by the procedure previously published by Ning et al. (19). Briefly, approximately 30 to 50 mg of silica-dried cotton leaf tissues from each sample was used for genomic DNA isolation using Qiagen Plant DNeasy Mini kit (Qiagen, Germantown, MD). After QC assessment with spectrophotometer and agarose gel electrophoresis, total of 300 ng to 500 ng of genomic DNA from each sample was subjected to the Illumina DNA-Seq library preparation using NEBNext Ultra II FS DNA Library Prep Kit (New England Biolabs, Ipswich, MA) with 3-cycles PCR enrichment and barcoding using NEBNext Multiplex Dual Index Primers Set (New England Biolabs, Ipswich, MA). The quality of each barcoded library was validated by the Agilent Bioanalyzer 2100 system (Agilent Technologies, Santa Clara, CA) and the Qubit fluorometer (Life Technologies, Grand Island, NY). We pooled 10 to 11 barcoded libraries equimolarly and sequenced (paired-ended 150 bp) on one lane (raw data output of 800 GB) of NovaSeq S4 system (Illumina, San Diego, CA) by Novogene Corporation Inc. (https://www.novogene.com/us-en/; Sacramento, CA).

Genetic Variant Calling

We first confirmed that the newly collected 299 samples represent wild populations, as opposed to escaped cultivated cotton, by comparing the newly generated data with existing *G. hirsutum* sequencing data and selected outgroup taxa. Resequencing data from three previously characterized wild *G. hirsutum* populations were downloaded from NCBI (PRJNA1086501, PRJNA603025, and PRJNA1226603), including samples from Mound Key, Florida (n = 25) (19), Pitahaya, Puerto Rico (n = 5), and Guadeloupe (n = 21) (20). An additional 10 representative

individuals from each of the three *G. hirsutum* domesticated pools were also downloaded (PRJNA414461; Table S3), representing modern cultivars (Cultivar) and both clusters of accessions broadly representing perennial and early domesticates as well as feral cottons (loosely termed Landrace1 and Landrace2; LR1 and LR2) (21). Two outgroup species were also selected, including nine wild *G. barbadense* (20, 21) and three *G. mustelinum* (PRJNA414461), the latter species phylogenetically sister to *G. barbadense* and *G. hirsutum*.

Each new and existing sample was genotyped using the Sentieon v202503.01 pipeline (22). In brief, raw reads from all 392 samples were individually trimmed via Trimmomatic v3.1.4 (23) to remove adapter sequences and low-quality bases were then mapped to the *de novo* assembled reference genome TX2094 via BWA v0.7.17 (24) in Sentieon. Samples were filtered for optical duplicates using ("*Dedup*") in Sentieon and indels were realigned ("*Realigner*") prior to Base Quality Score Recalibration ("*QualCal*") and gVCF generation.

Joint-genotyping of gVCFs was conducted by species or geographic region (as an initial proxy for population), dividing the 392 individual samples into seven groups to generate final VCFs: (1) all domesticated cotton; wild cotton from (2) Florida (including Mound Key), (3) Puerto Rico, (4) Guadeloupe, and (5) Yucatán for *G. hirsutum*; (6) *G. barbadense;* and (7) *G. mustelinum*. Each population was joint-genotyped individually and both variant and invariant sites were emitted in Sentieon ("*--emit_mode all*"). We used VCFtools v0.1.16 (25) to remove indels ("*--remove-indels*") and filter sites whose sequence depth was <10 or >100 ("*--min-meanDP 10 --max-meanDP 100*"), implying low coverage or repetitive sequences, respectively. For variant sites, we additionally required no missing data and retained only biallelic sites ("*--max-missing-count 0 --max-alleles 2*") where each biallelic SNP occurred at least twice among samples ("*--mac 2*") to remove potential errors. Eventually, all seven VCFs with variant and invariant sites were merged together using bcftools v1.19 (26) and only biallelic across all 392 individuals with no-missing data ("*-m2 -M2 'F_MISSING=0'*") were refiltered for population structure analysis.

Determining Genetic Structure and Relationships among Wild Cotton Populations

From an initial set of 36,984,819 genome-wide biallelic SNPs across 392 samples (including outgroups; hereafter Set1_n392), 21,613,172 variants passed the minor allele frequency filter of 1% ("*-q 0.01:minor*") in bcftools. Further thinning of sites in high linkage disequilibrium (LD; "--indep-pairwise 50 10 0.1") was conducted using PLINK v1.9 (27), yielding a final dataset of 2,369,270 SNPs.

To determine the major genetic groups among all samples, principal component analysis (PCA) was performed in PLINK. The first 20 principal components ("*--pca 20*") were retained, and the first two PCs were plotted using the R (28) package ggplot2 (29). In addition, a genetic distance matrix for all 392 samples ("*--distance square 1-ibs*") was tabulated using PLINK on the same SNP set; this distance matrix was subsequently used as input to draw a neighbor-joining tree via the R package ape (29), which was visualized using ggtree (30). Finally, LEA (31) was used to infer genetic structure from the same 2.4 million (M) SNPs across 392 samples. The number of ancestral populations (K) considered ranged from 1 to 30, testing 10 replicates each. The best K was selected based on mean cross-entropy values.

We then focused on relationships within *G. hirsutum* by removing outgroup samples ("*-S*") using bcftools and rerunning the above analyses. Specifically, we refiltered for biallelic SNPs with no missing data and a minor allele frequency above 1% using the original 37.0 M SNPs. These filters retained 15.2 M SNPs, which were thinned to 1.9 M SNPs based on LD pruning in PLINK. *Gossypium hirsutum*-focused PCA and neighbor-joining tree were each created using the same methods described above. Similarly, two sets of VCFs containing LD-pruned biallelic SNPs with minor allele frequency above 1% were filtered for wild cotton samples from Yucatán (1.2 M) and Florida (1.1 M), respectively, to analyze the genetic relationships from each peninsula separately using PCA.

Estimating Gene Flow among Groups

Treemix (32) and Dsuite (33) were used with the original 2.4 M filtered biallelic SNPs from 392 samples (including outgroups) to estimate gene flow between the wild and domesticated cotton gene pools, as well as possible introgression from wild *G. barbadense* into domesticated *G. hirsutum* cottons. Briefly, allele frequencies at each SNP site were estimated using PLINK for the 10 defined groups identified via PCA and LEA (see Results), i.e., five wild cotton populations, three domesticated genetic groups from the germplasm bank, and the two outgroups, *G. barbadense* and *G. mustelinum*. TreeMix was used to infer population relationships and historical gene flow by testing migration edges (m) ranging from 0 to 8, with five replicate runs for each value of m. Each run implemented block-wise bootstrapping with a block size of 1000 SNPs (-k 1000) and standard-error estimation (-se). The best model (i.e., optimal number of migrations) was selected using the R package OptM (34). Dsuite then used the same 2.4 M SNPs and the maximum likelihood, no migration (m=0) topology from Treemix to estimate genomic introgression among the 10 defined groups using ABBA-BABA test statistics. *f*3- and *f*4-statistics were calculated using the same set of 2.4 million SNPs, based on allele frequency of each group, using PLINK and AdmixTools2 (34). For *f*3-statistics, three domesticated *G. hirsutum* groups were treated as targets, with five wild *G. hirsutum* populations and *G. barbadense* as sources; in the form (Gh_domesticated; Gh_wild, Gb). *f*4-statistics were computed in the form (Gh_domesticated, Gh_wild; Gb, Gm), using *G. mustelinum* (Gm) as the outgroup.

Because both natural and human-mediated introgression between *G. hirsutum* and its congener *G. barbadense* have been reported (35–37), we performed local ancestral inference via FLARE (38). We first excluded the three representatives of *G. mustelinum* from the original 37.0 M SNP (Set1_n392) and filtered to retain only biallelic sites (as above). Any site with fixed heterozygosity (i.e., all samples were heterozygous) were considered putative paralogous mapping sites and were subsequently removed, resulting in a new set of 36,948,960 SNPs across 389 samples. Genotypes for these samples were phased via Beagle v5.5 (39). Using these phased 36.9 M SNPs and a physical genetic linkage map of 2,030 filtered markers (40), local ancestry inference was computed in FLARE. The SNPs from the Yucatán cottons and *G. barbadense* representatives were first used as references to examine introgression in the other three wild cotton populations (Puerto Rico, Guadeloupe, and Florida). In addition, the three domesticated gene pools (LR1, LR2, and Cultivar) were compared to the SNPs of all wild cotton populations and *G. barbadense* to estimate the proportions of different ancestral contributions that comprise each group.

Kmer-based Genetic Relationship Inference between Major Genetic Groups

In addition to the SNP-based methods, we assessed genetic relationships among the 10 genetic groups identified in the analyses described above using KmerCity (41) to characterize kmer polymorphisms within the repetitive genomic regions. In brief, two to five representative individuals were randomly selected from each sample site of wild cotton (total 158 samples; Table S4): Florida (66), Yucatán (40), Puerto Rico (5), and Guadeloupe (5); in addition, 30 samples were included from the domesticated cotton gene pools, as well as nine *G. barbadense* and three *G. mustelinum*. Trimmed reads from each selected sample were filtered against a contaminant database ("Standard-8") via Kraken2 (42), and all organelle sequencing reads were subsequently removed using mirabait (https://github.com/DrMicrobit/mira) in conjunction with the newly generated cpDNA reference sequence from TX2094 and the published mtDNA of *G. hirsutum* (NC_027406.1). From the final filtered datasets, 6M forward and 6M reverse reads were subsampled per sample to quantify 50bp kmer diversity and frequency using KmerCity. For downstream comparisons, the resulting kmer counts were summarized in R by calculating the average occurrence of each kmer category (i.e., different kmer sequences) across all individuals within each genetic group. Because we wanted to focus on those kmer patterns that vary within *G. hirsutum*, particularly when some variation was shared with *G. barbadense* but not *G. mustelinum* (indicating possible introgression from *G. barbadense*), we filtered the output table to remove kmers (1) present in all groups; (2) absent (i.e., count <1) in each group; (3) present exclusively in *G. barbadense* and *G. mustelinum*; or (4) present in only one of the *G. hirsutum* populations and in *G. mustelinum*.

Plastome Variation
Whole chloroplast DNA (plastome) of the newly sequenced 299 Florida and Yucatán cottons were *de novo* assembled from the trimmed sequencing data via Getorganelle v1.7.7.0 (43). In addition, plastome sequences were retrieved from three previously identified wild cotton populations (n = 51), three groups of domesticated cottons (n = 30; Table S3), wild *G. barbadense* (n = 9), and the outgroup *G. mustelinum* (n = 3) (19). We also included three plastome sequences from the model maternal diploid ancestor (*G. herbaceum*) as a more distant outgroup for polyploid cottons (44). All plastomes (total n = 395) were aligned using MAFFT v7.505 (45) and any sites with gaps were trimmed using trimAl v1.5.rev0 (46). A maximum likelihood tree was reconstructed from the trimmed alignment using IQTEE2 v2.2.2.7 (47) with best model selection via ModelFinder. After confirming the root position of the outgroup in the plastome phylogeny, we collapsed the nodes where bootstrapping values were below 80 using Newick Utilities ("nw_ed") (48) and visualized the final result in ggtree in R.

Genomic Diversity Comparison between Wild Cottons
To quantify nucleotide diversity (π) in wild cotton (total n = 350) and to compare these estimates with those of the domesticated gene pool (n = 30), we removed fixed heterozygous sites from the combined VCFs in each major group via bcftools, and rejoined all seven VCFs from different groups into one using bcftools. Using Pixy v2.2 (49), global (i.e., all wild cotton populations grouped as one) and regional (i.e., individual wild cotton populations) genetic diversity π was quantified in 10 kbp sliding windows. In addition, nuclear sequence divergence (d_{XY}) was also calculated in Pixy for each pairwise comparison using the same 10 kbp windows. Moreover, biallelic SNPs without missing data were refiltered for the 380 samples representing wild and domesticated *G. hirsutum* using the above rejoined VCF (hereafter Set2_n380). This filtered VCF containing 28.6 M SNPs was used to calculate the proportion of observed heterozygous sites and inbreeding coefficient (F_{IS}) in each sample via VCFtools. Inbreeding was further surveyed by estimating the proportion of each genome exhibiting long runs of homozygosity (ROH) above 0.25 Mbp using the R package detectRUNS v.0.9.6 (50).

Genetic Structure within Yucatán and Florida Cottons
To provide additional resolution of genetic structure within wild cotton populations from Yucatán and Florida, and to assess their relationships with the domesticated cottons, we also analyzed the samples collected from each peninsula separately. Specifically, two sets of VCFs containing biallelic SNPs were refiltered from the above (Set2_n380) to include (1) only wild cotton samples from Yucatán or Florida, or (2) wild cotton samples from Yucatán or Florida plus representatives of the three domesticated gene pools (Table S3). The first set of VCFs, containing a single set of samples, was used to estimate the genetic relatedness ('--genome') among samples in PLINK. The second set of VCFs, containing wild cottons from Yucatán or Florida combined with 30 germplasm representatives, was used to construct neighbor-joining trees and to infer genetic structure with LEA, following the methods described above. In addition, we evaluated the effect of distance on interpopulation divergence by tabulating the d_{XY} calculated by Pixy (above) for each site in Yucatán and Florida (Fig. 2a, 2b) and correlating it with their geographic distance using Pearson correlation in R package Smplot (51).

The northeastern Yucatán population had higher genetic relatedness (PI_HAT) between samples (average PI_HAT = 0.58 vs 0.17 in all northwestern populations; Fig. S9), suggesting that the northeastern (YUC-E) RiCh/RiCa sites represent a smaller, more isolated and localized population relative to the main populations from the northwestern part of the peninsula (Fig. S11a, S11e). Congruent with the foregoing, d_{XY} between each Yucatán site also was positively correlated with geographical distance ($p < 0.001$; Fig. S11c). In contrast, the average genetic relatedness between all Florida cottons was about 0.50 (Fig. S10), with the middle- to upper-north populations (MK/NP/TC) showing weaker signals of admixture from multiple ancestral sources compared to the southern tip populations (e.g., RBD/RNRB/CPT) (Fig. S11b, S11f). Florida

cottons exhibited no clear correlation with geography, an observation reiterated in the low correlation between pairwise d_{XY} and geographical distance (not significant, $p = 0.394$; Fig. S11d).

TE Insertion Polymorphisms in Wild Cotton

Transposable elements (TE) are a major component of plant genomes, and their insertion polymorphisms (TIPs) can reveal differences in the evolutionary (genomic) histories of various populations (52, 53). Using KmerCity pruned organelle-free reads, we identified and compared TIP variation across 116 wild *G. hirsutum* samples, while excluding domesticated cotton and outgroup accessions (Table S4), and subsetting an equal amount (131M paired-end reads, reflecting the sample with the lowest sequencing depth) for each sample using seqkit v 2.9.0 (54). Then SWIF-TE (55) was applied to identify putative TE insertion positions by using BWA to map the short read data from each sample to both the TE library and the TX2094 reference genome ("soft-clip threshold 40"), and locate within read-break points consistent with a TIP in that genomic region. All outputs were cleaned with "clean_SWIFTE.sh", using a minimum read count support (for each TIP) of 3. The final results were tabulated in R to keep a minimum TIP occurrence of 3 (out of 116 individuals).

Genetic Burden Comparison between Wild Cottons

Genetic load, which refers to the accumulation of deleterious mutations in a population, can provide insight into past demographic changes or human-mediated selection (56, 57). Genetic load is typically estimated by comparing the predicted functional consequences of SNPs against known reference databases, using either DNA sequence or translated amino acid sequences. To investigate differences in genetic load among wild *G. hirsutum* populations, we employed two complementary approaches: GERP++ (genomic evolutionary rate profiling) (58) and SIFT4G (59). To ensure accurate estimates of genetic load, we restricted our analysis to sites conserved between TX2094 and the outgroup (*G. mustelinum*), allowing us to infer ancestral alleles and avoid bias from deleterious variants present in the reference genome. Specifically, we filtered the original VCF (Set1_n392) to contain only biallelic SNPs from all wild *G. hirsutum* (n = 380) and one representative of *G. mustelinum* (hereafter Set3_n381), and then subsequently retained only those positions where the outgroup (*G. mustelinum*) was the same as reference (i.e., "0/0").The final VCF (containing 18.9 M sites) was then annotated with each of the two approaches to characterize putative deleterious alleles/sites in each sample.

SIFT4G predicts whether nonsynonymous mutations may be deleterious or tolerated based on the degree of conservation across homologous proteins. Here, the SIFT4G database ("SIFT4G_Create_Genomic_DB") was constructed using the protein predictions from TX2094 against the SWISS-PROT database (60). Only VCF sites annotated as deleterious (excluding the low confidence sites) were retained; weighted SIFT scores (SIFT score < 0.04; range 0–1) were calculated using the equation 1 - (SIFT_SCORE) / 0.04) (61). The genetic load was tabulated for each sample using (1) an additive model (i.e., the number of derived allele * 0.5 * weighted SIFT score), and (2) a recessive model (genotype 1/1 = 1* weighted SIFT score), as previously described (62). Genetic load differences among groups were compared using ANOVA and TukeyHSD tests in R.

GERP++ compares the observed substitution rate at any given site to the expected neutral evolutionary rate, calculated from a phylogeny across distantly related taxa, to identify mutations at highly conserved sites, which may indicate potential deleterious effects. These effects are expressed as the rejected substitution (RS) score, which are calculated on a site-by-site basis (for details, see https://github.com/Wendellab/Wildcotton_YUCFL/blob/main/05_WildCottonTEandGeneticload/02_GERP_Pipeline.md). Here, we downloaded 14 phylogenetically distant reference genomes with repetitive regions masked from NCBI, including 11 dicots and 3 monocots (Table S5), and aligned these to the TX2094 reference genome with Progressive Cactus v.3.0 (63). A neutral phylogenetic tree for all 15 species was constructed in IQTREE2 ("-m GTR"), using four-fold

degenerate neutral sites, as annotated by Degenotate (64). The RS score at each site was calculated using “gerpcol” in GERP++, and only RS score > 4 were considered as highly deleterious (57). Finally, the genetic load for each sample was analyzed using the two models, i.e., additive and recessive, as noted above.

Signatures of Selection Under Domestication

Genomic selection scans can provide insight into genome evolution following domestication. Selection signals were evaluated by genome-wide comparisons between 109 accessions (20) representing the global diversity in modern cultivars (Table S7) and 118 (northwestern) Yucatán cottons, the latter of which represent the center of domestication (see Results). Three independent approaches were used to identify putative regions of selection: (1) π (nucleotide sequence diversity), (2) Fst (population differentiation fixation index), and (3) XP-CLR (cross-population composite likelihood ratio; 65). Specifically, raw resequencing reads for 109 cultivar accessions were retrieved from NCBI (20), and were joint-genotyped with all western Yucatán populations following the aforementioned pipeline and retaining only variant sites using Sentieon (‘emit_mode variant’). The final VCF contained 23.5 M biallelic variants (hereafter Set4_n227), which were present in 227 samples. This filtered VCF was used (1) to infer population genetic relationships via neighbor-joining tree and PCA after LD pruning; (2) to calculate π and Fst by VCFtools, and (3) estimate selective sweep likelihood values using XP-CLR. For all analyses, we applied a 10 kb window size with a 2 kb step size, and genomic regions falling within the top 5% of values in all three methods were identified as putative regions of domestication. Protein sequences for the coding genes contained within these regions were extracted using R package rtracklayer (66), and then blasted against the Swiss-UniProt *G. hirsutum* database to identify ortholog gene IDs (67). The final gene list was analyzed for gene ontology (GO) enrichment for Biological Processes using PANTHER (68).

Supplemental Figures

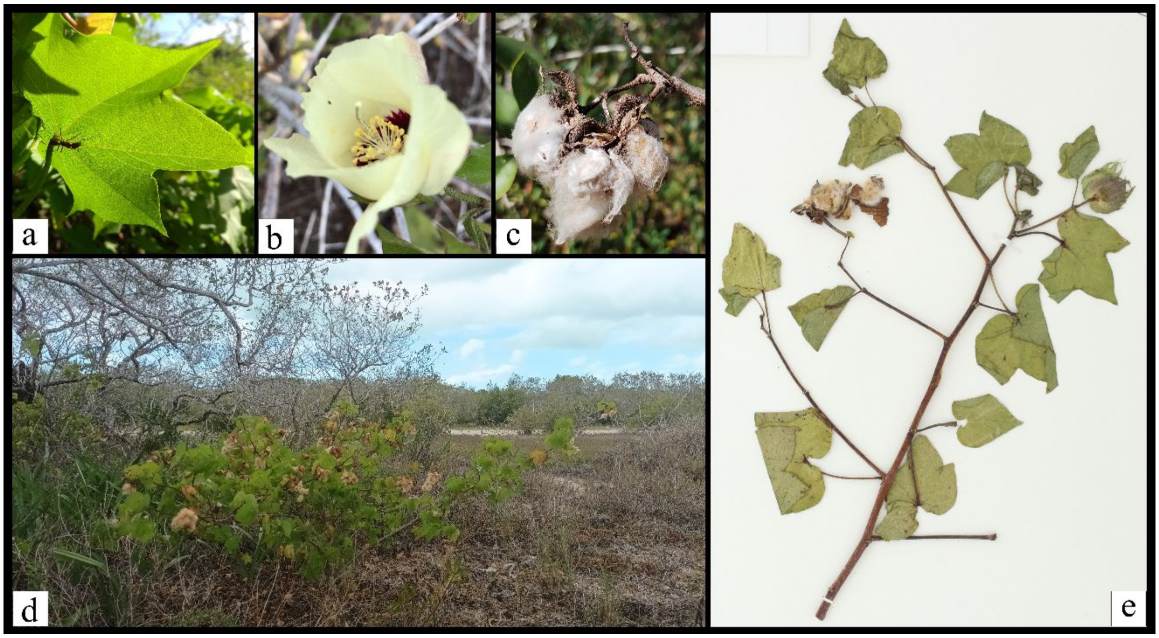

Fig. S1. Morphology and habitat of wild *G. hirsutum* from the Yucatán Peninsula (México). (a) Back of leaf showing gossypol-containing lysigenous cavities (punctae), which are used as direct defense against herbivores. An ant is tending an extrafloral nectary, which are used to recruit predators of insect herbivores (69). Photo credit to T. Quijano-Medina. (b) Flower morphology, showing the exerted style, monadelphus stamens, creamy-white petals, and dark petal spots. (c) Wild cotton capsule (colloquially "boll") in a natural setting (d). (f) Herbarium sheet of wild-collected *G. hirsutum* (ISC accession 457835).

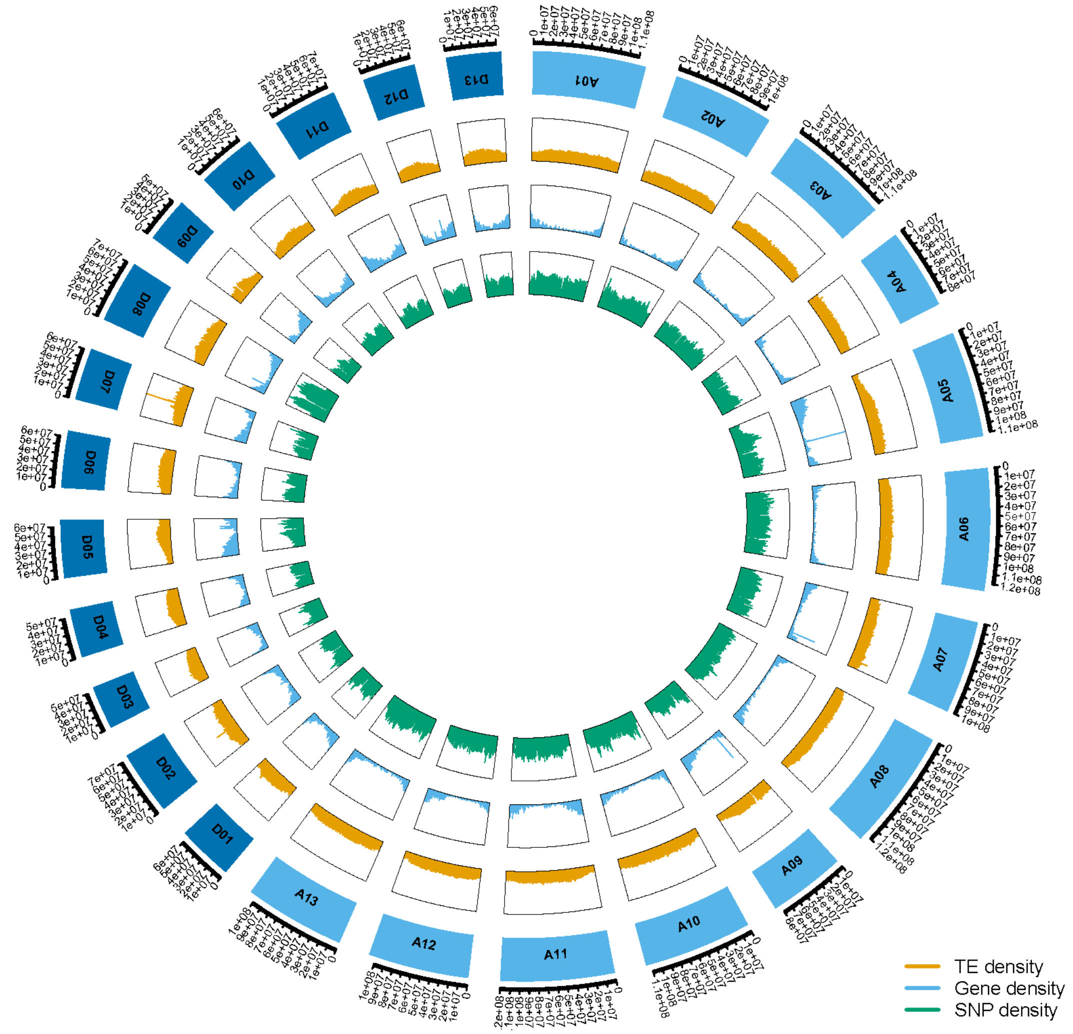

Fig. S2. Assembly of reference genome TX2094. The outermost circle shows chromosome positions of the two subgenomes, A and D. The second to fourth circles (from outside in) display 1 Mbp density plots for TEs, genes, and SNPs (using *G. hirsutum* only VCF Set2_n380), respectively.

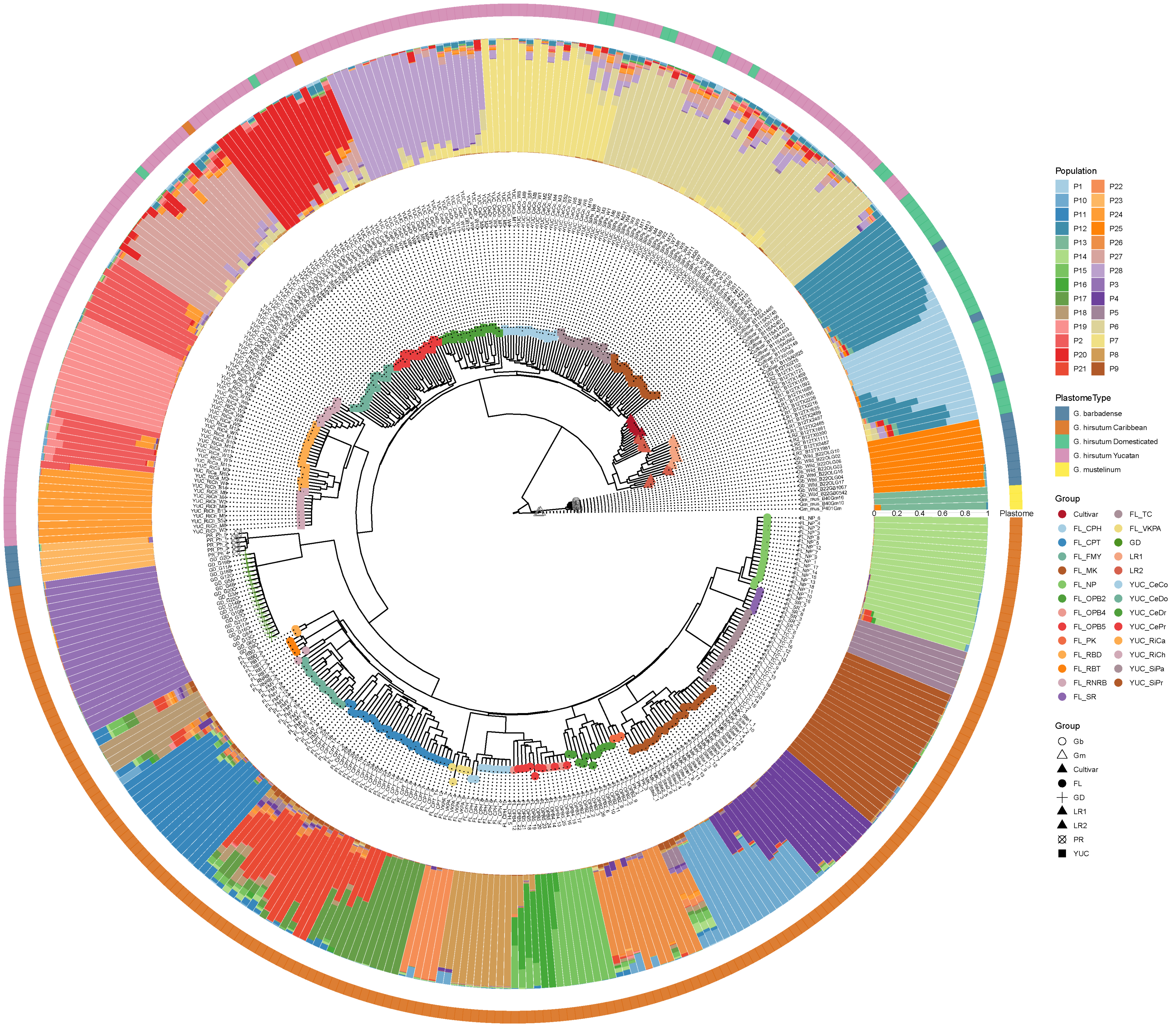

Fig. S3. Genetic relationships among 380 *G. hirsutum* individuals, including nine *G. barbadense* and three *G. mustelinum* outgroups. The inner circle depicts a neighbor-joining tree rooted on *G. mustelinum* where each terminal tip is labelled with an individual ID and a symbol. Color-filled symbols represent different geographic sites or groups. The middle circle consists of barplots showing the genetic structure of 392 individuals, where each bar is filled with colors proportional to the inferred ancestor populations (28 total predicted ancestral populations; see Fig. S4). The outer circle depicts the inferred plastome groups using reconstructed phylogeny of whole plastomes (see Fig. S4).

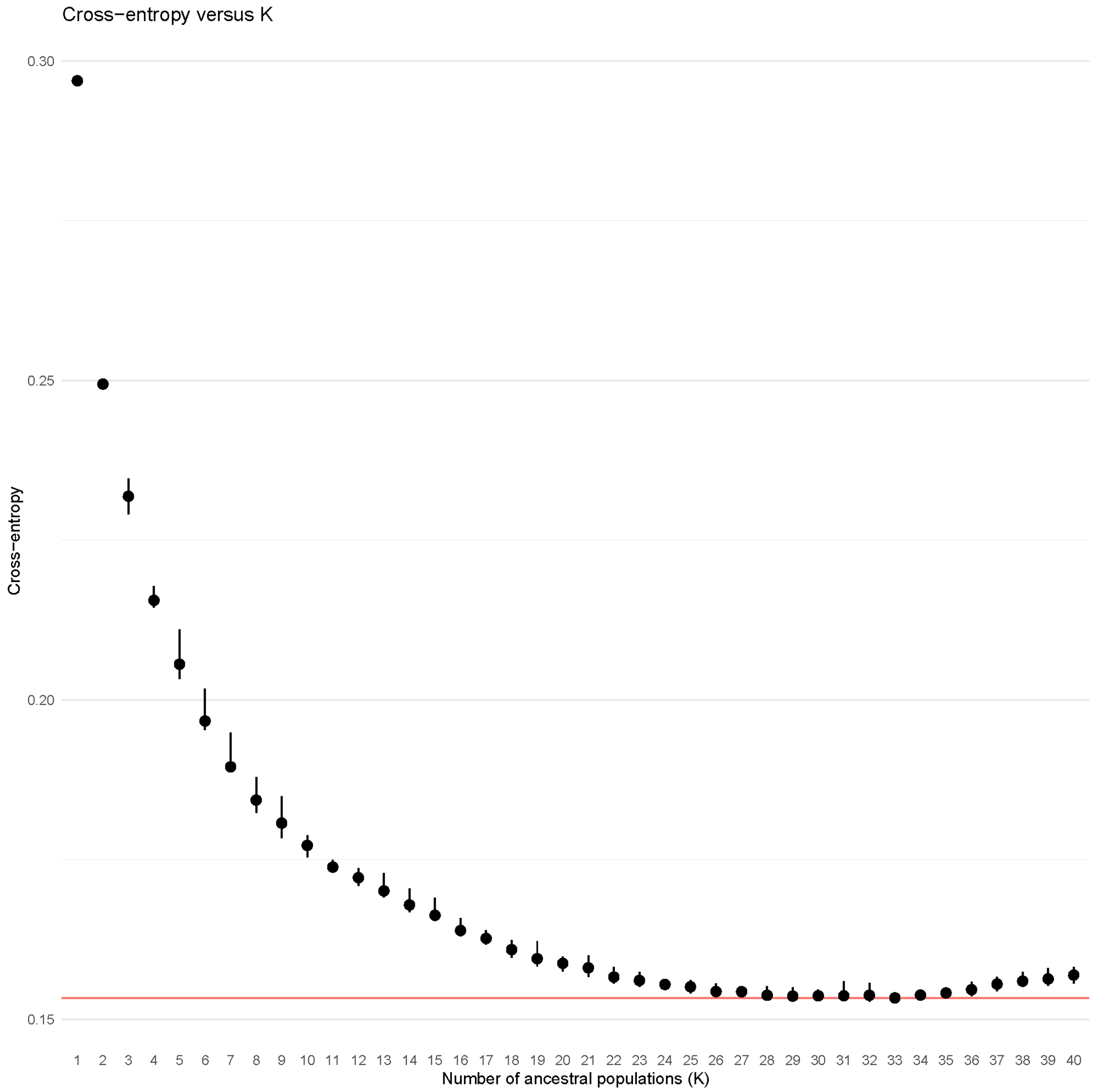

Fig. S4. Selection of the best number of ancestral populations (K = 28; x-axis) based on the LEA cross-entropy values (y-axis). The red horizontal line crossing the black dots represents the standard deviation across 10 replicates.

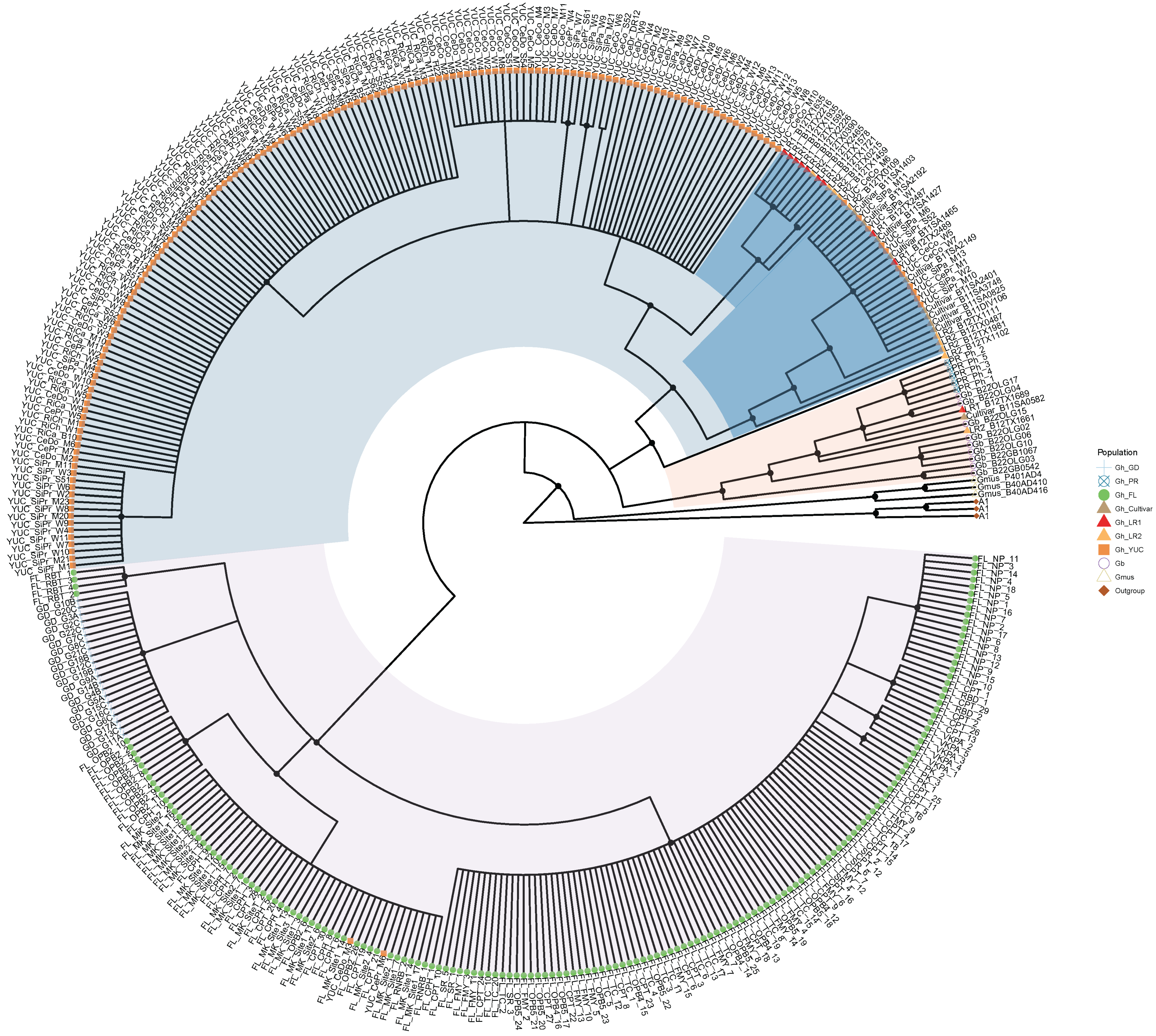

Fig. S5. Plastome phylogeny shown without branch lengths. Nodes with bootstrap support below 80 are collapsed, and above 90 are highlighted with black dots.

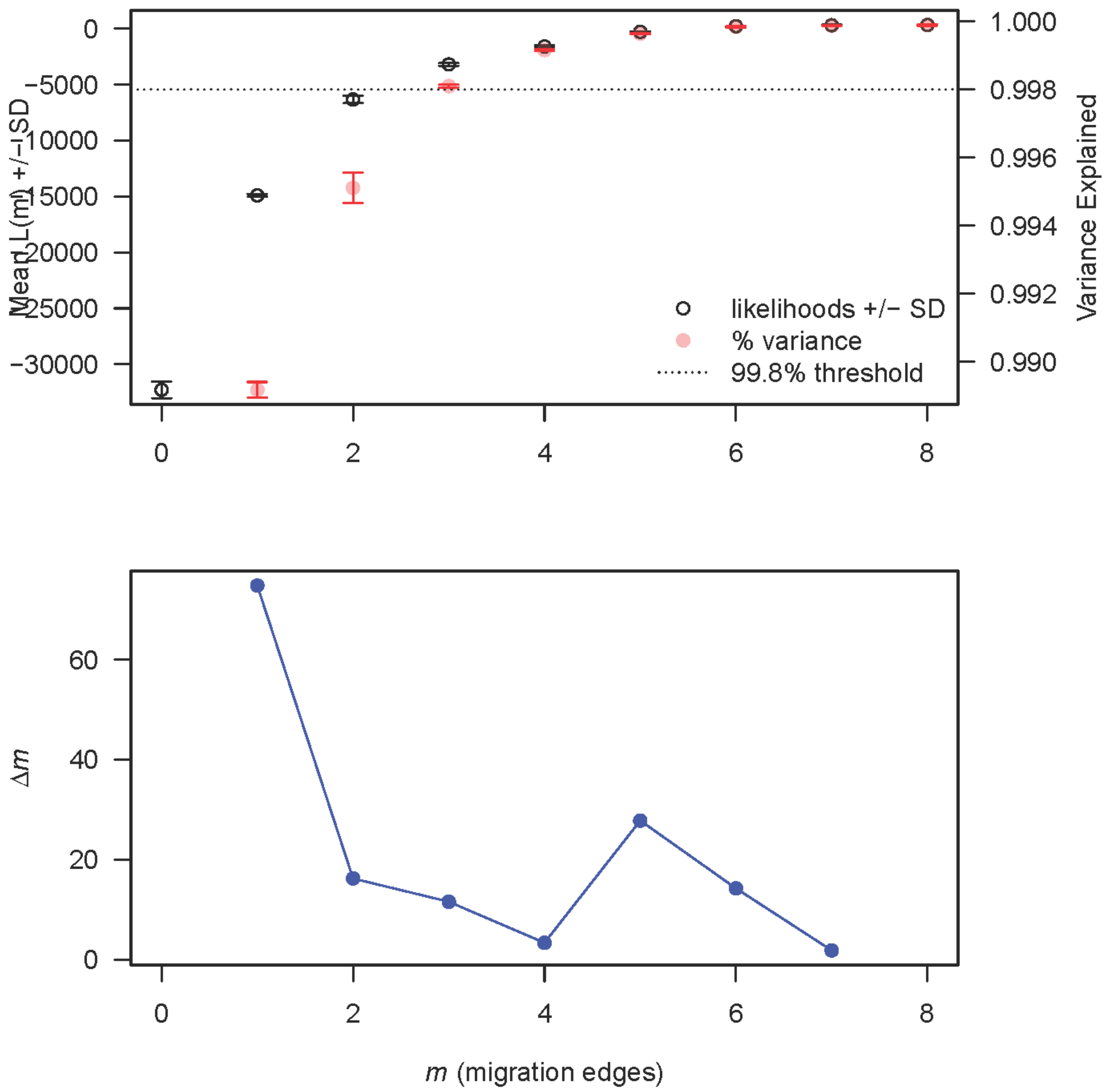

Fig. S6. Model selection for TreeMix results from optM showing the change in model fit (delta m) across increasing numbers of migration edges (m), and the elbow point indicates the optimal number of migration edges (m=5).

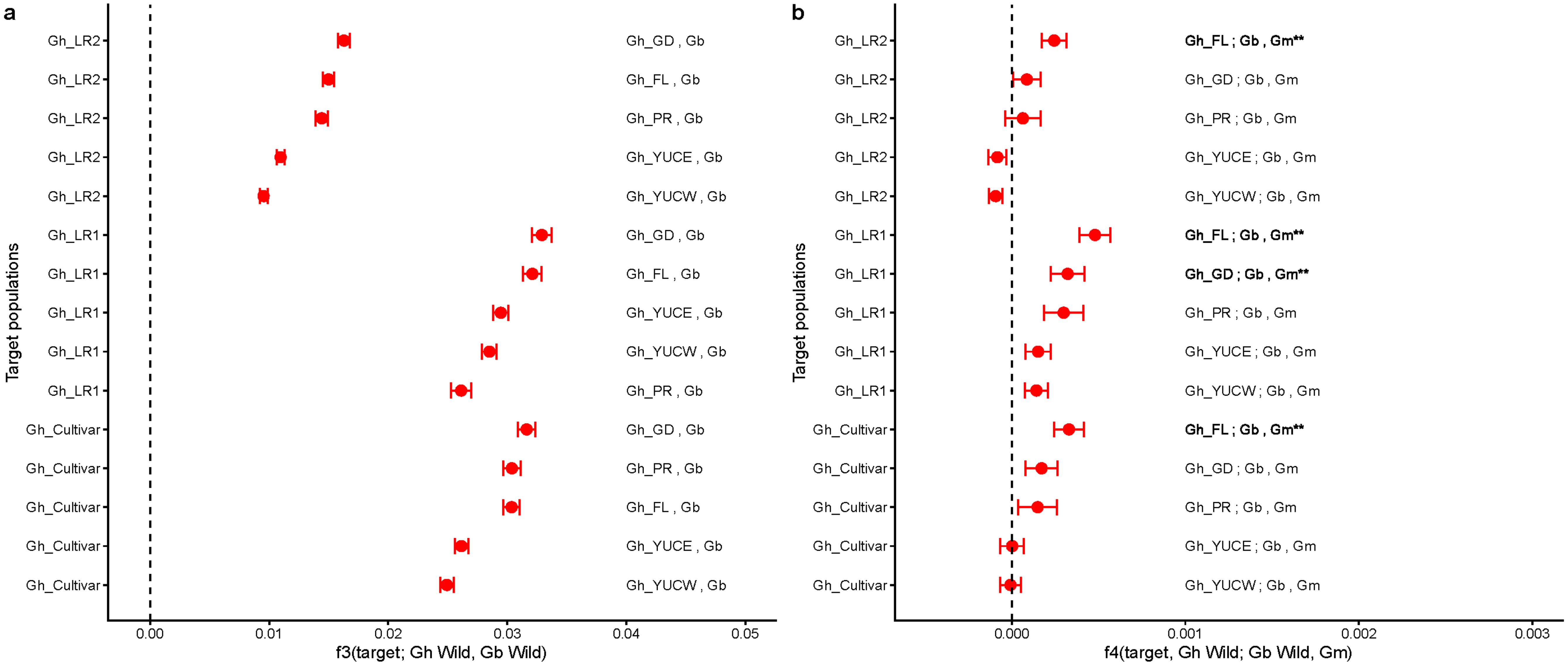

Fig. S7. Admixture tests using *f*3- and *f*4-statistics with points show *f*3 (a) and *f*4 (b) estimates values ± standard error; stars (*) indicate significant pairs based on Z-scores ($|Z| \geq 3$). (a) *f*3-statistic was calculated in the form f3(target; Gh wild, Gb wild). The target populations are shown on the y-axis, and the x-axis shows *f*3 values. The corresponding source population pairs for each test are labeled on the right side of the plot. (b) *f*4-statistic was calculated in the form *f*4(target, Gh Wild; Gb, Gm), where Gm was used as the outgroup.

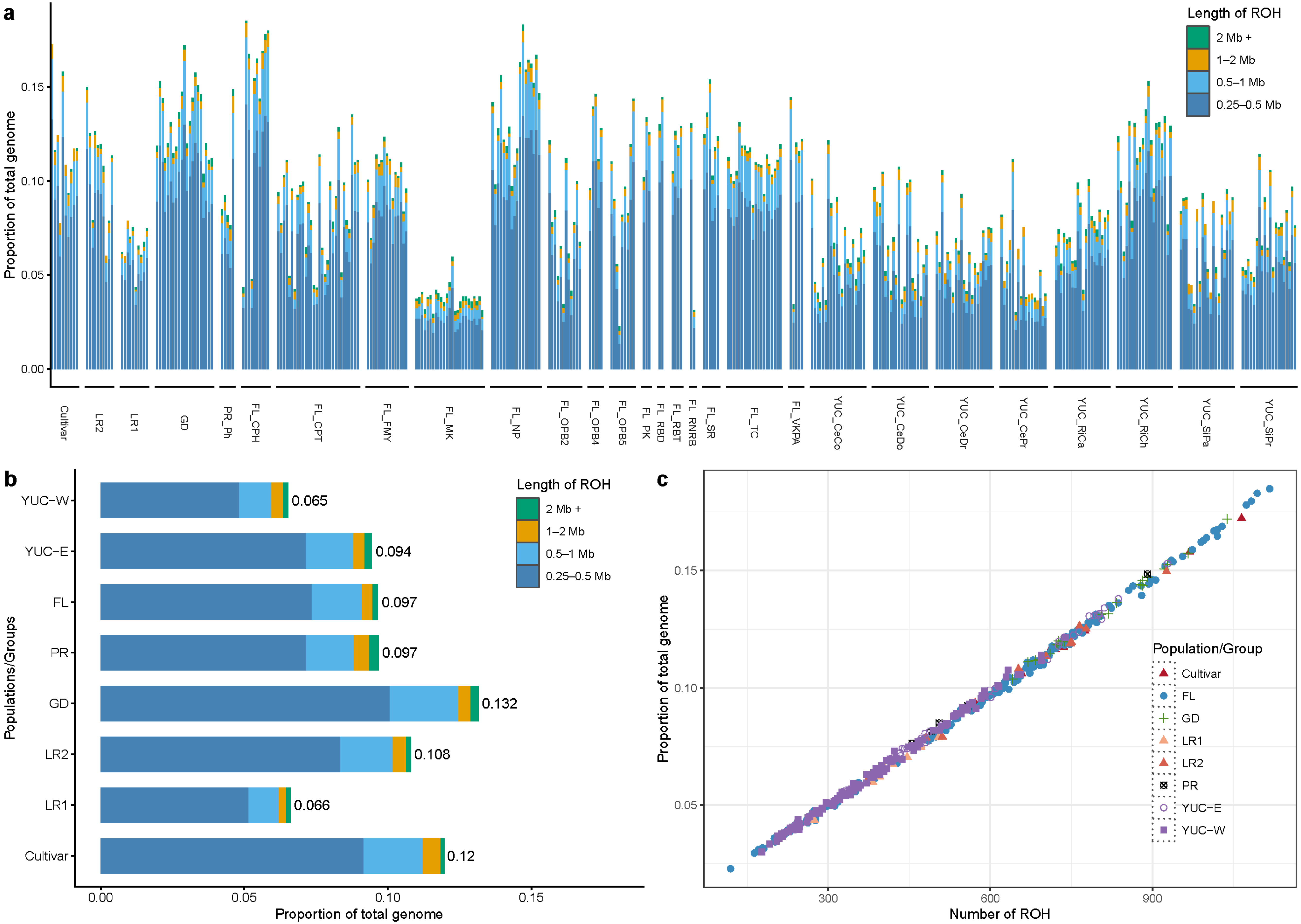

Fig. S8. ROH (Runs of Homozygosity) analysis for each sample. (a) Individual genomes showing proportions of ROH > 0.25 Mbp categorized into three size classes. (b) Average ROH proportions per wild cotton population. (c) Correlation between number of ROH fragments and total genome proportion in ROH.

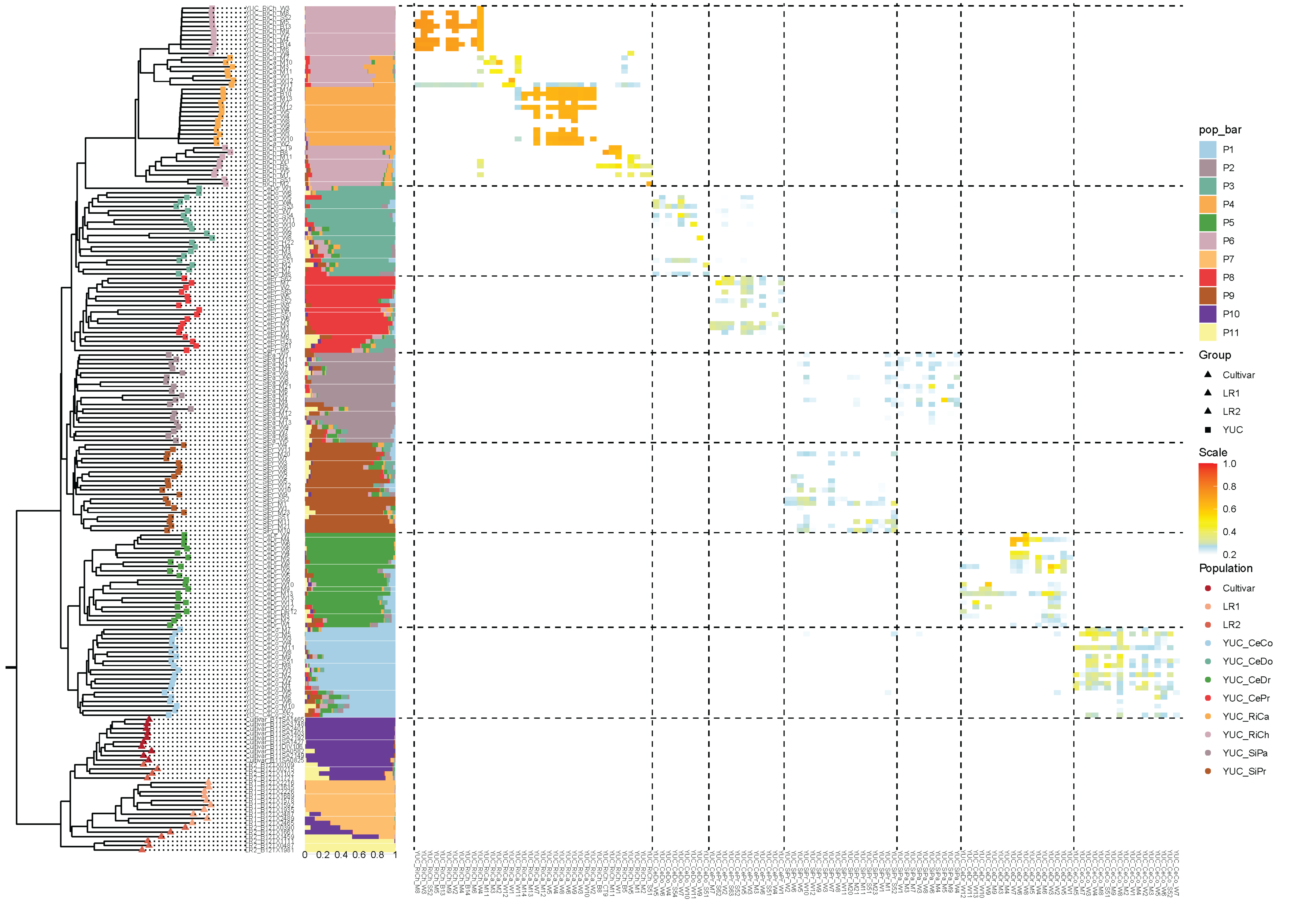

Fig. S9. Genetic structure within Yucatán cottons. The neighbor-joining tree and LEA genetic structure results on the left show the genetic relationships between Yucatán and domesticated cottons. The heatmap on the right shows the genetic relatedness (PI_HAT values) among all Yucatán samples.

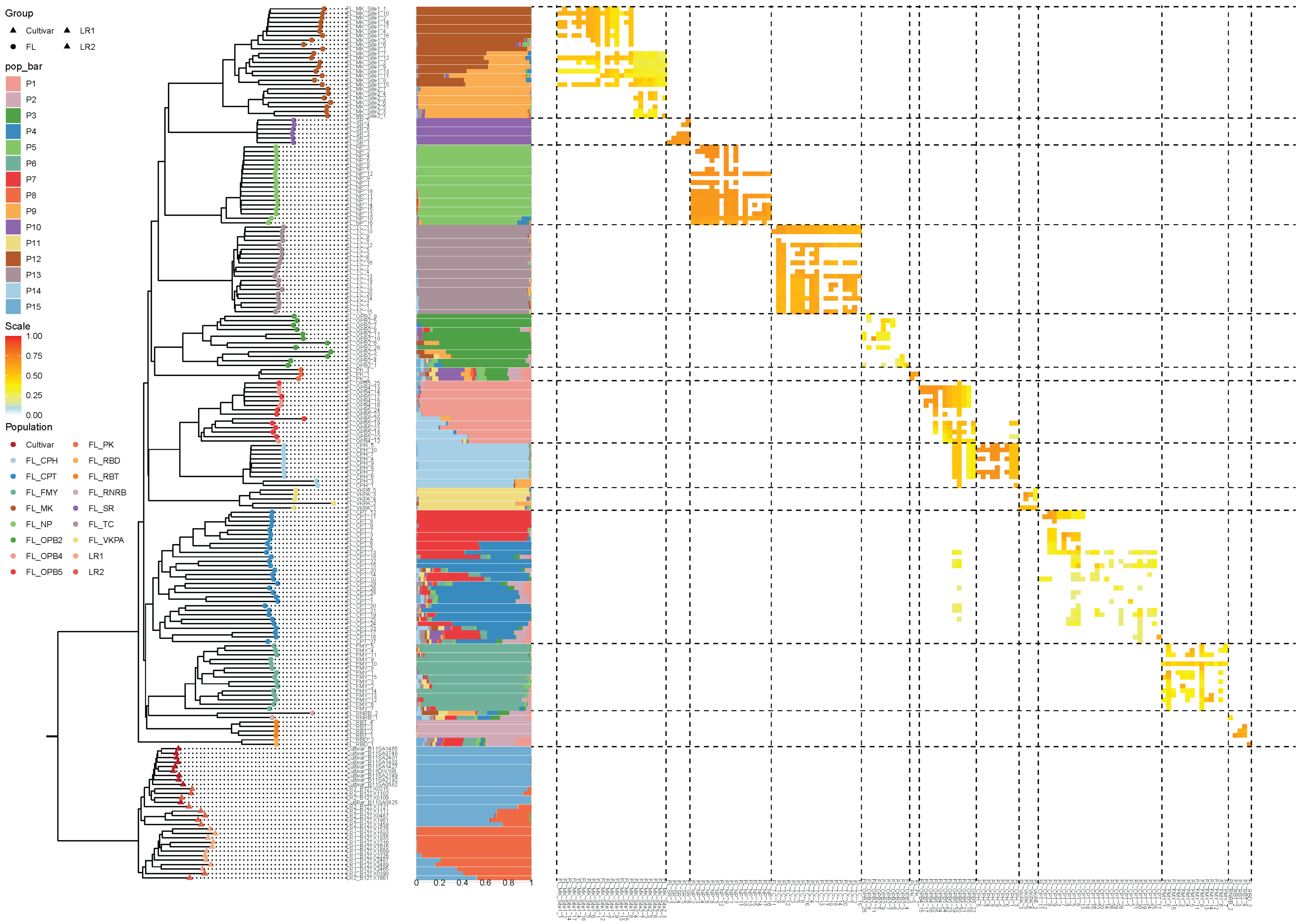

Fig. S10. Genetic structure within Florida cottons. The neighbor-joining tree and LEA genetic structure results on the left show the genetic relationships between Florida and domesticated cottons. The heatmap on the right shows the genetic relatedness (PI_HAT values) among all Florida samples.

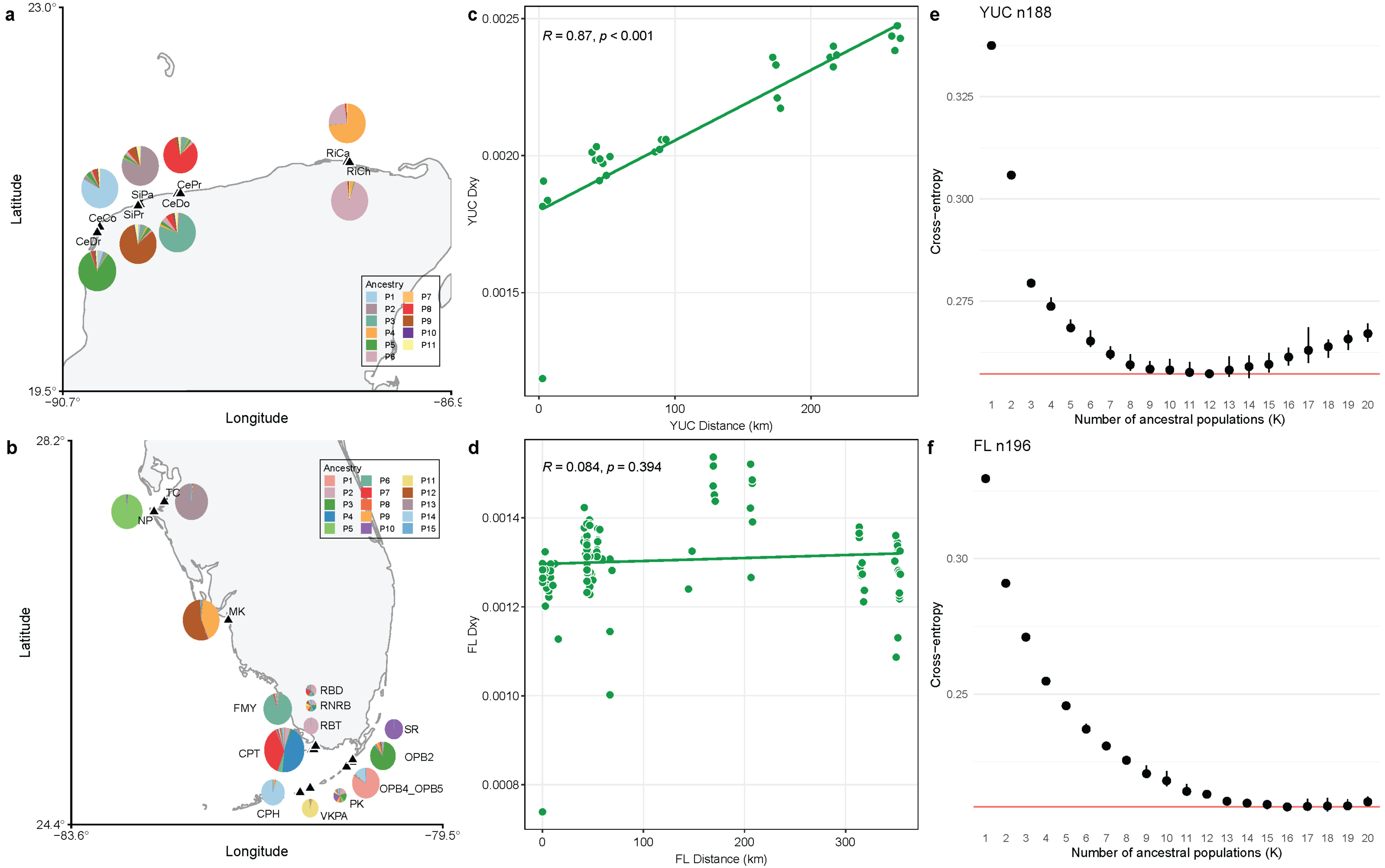

Fig. S11. Genetic variation within Yucatán and Florida populations. (a) and (b) LEA genetic structure analysis, with pie charts (size was scaled by number of individuals) representing the average proportion of ancestry of samples from each site. Expanded figures are shown in Fig. S9 and Fig. S10. (c) and (d) Correlation analysis of pairwise genetic sequence divergence (d_{XY}) and physical distance between sites. (e) and (f) LEA model selection for Yucatán and Florida genetic structure analysis.

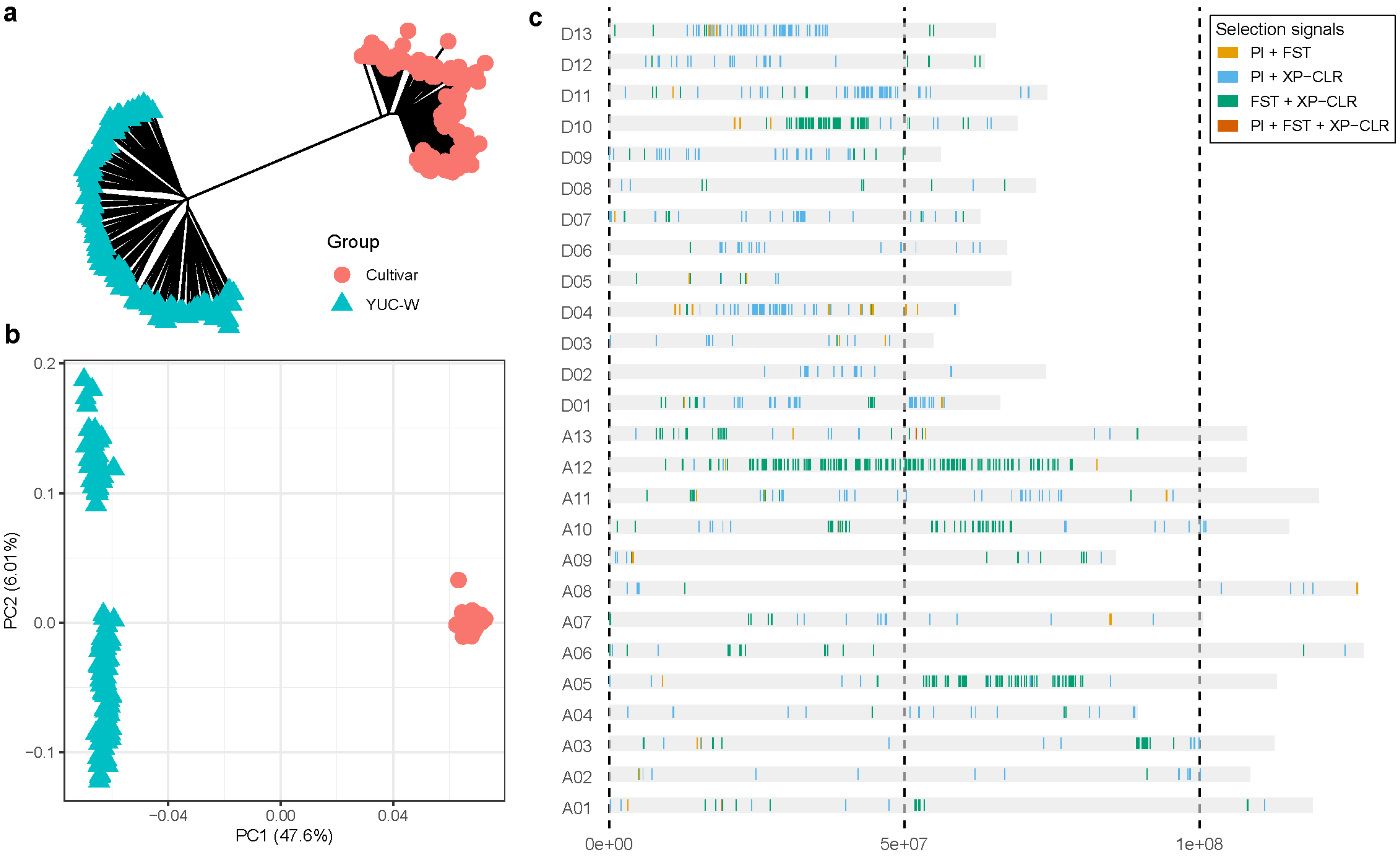

Fig. S12. Scans for signals of selection under domestication in cotton. (a) Neighbor-joining tree and (b) PCA for 109 cultivar accessions and western Yucatán population. (c) Genomic regions under domestication, identified as the top 5% of values in at least two of the three methods: π ratio ($\pi_{YUC\text{-}W}/\pi_{Cultivar}$), Fst, and XP-CLR, for each chromosome. Chromosomes are individually listed, with coordinates along the x-axis, and colors denote the combination of methods supporting selection in those regions.

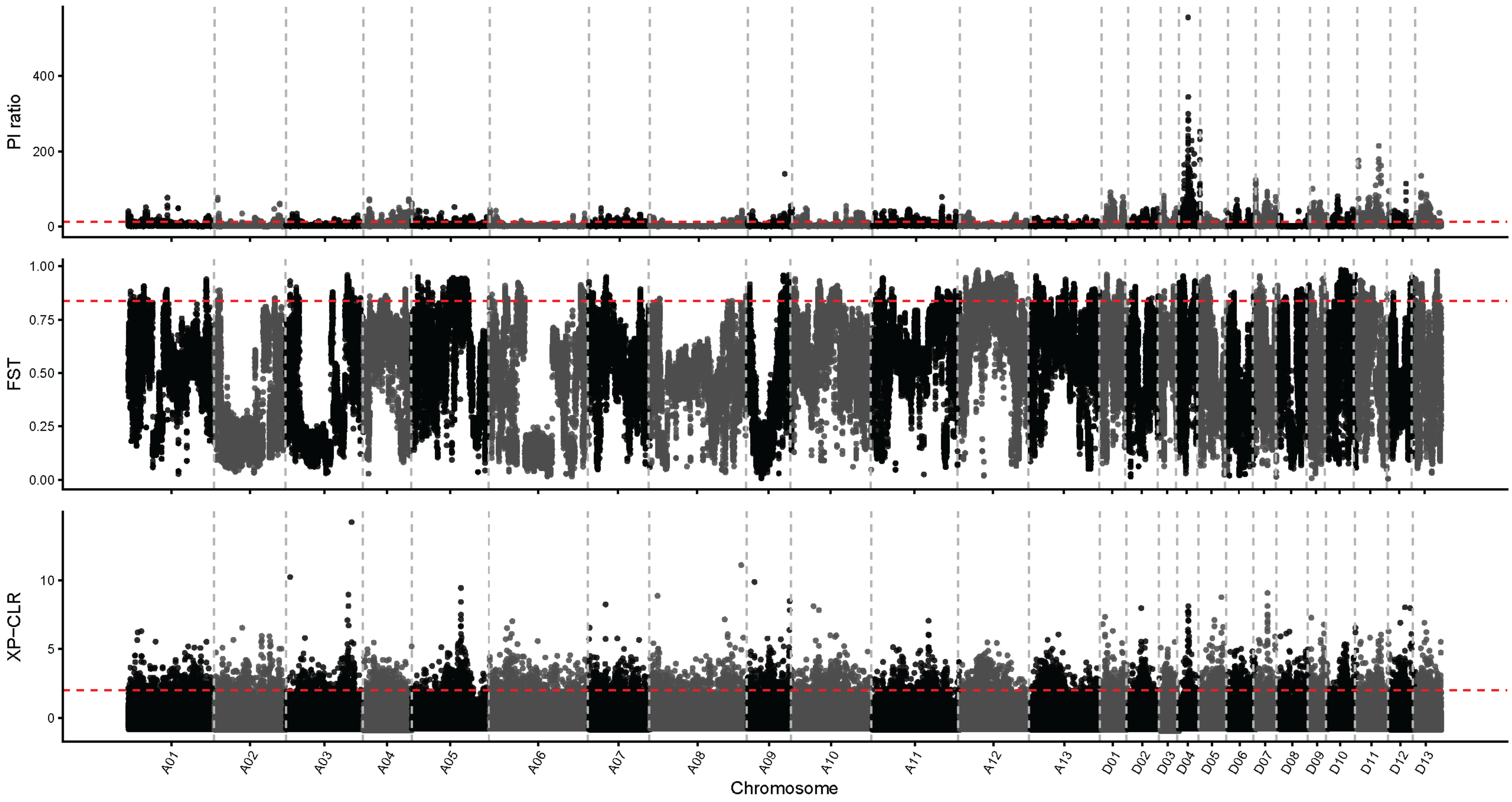

Fig. S13. Manhattan plots for (a) π ratio ($\pi_{YUC-W}/\pi_{Cultivar}$), (b) Fst, and (c) XP-CLR, with the top 5% values indicated by a horizontal red dashed line

Supplemental Tables

Table S1. The assembled genome of TX2094.

Descriptor	TX2094
NCBI reads	PRJNA1045685
# PacBio Hifi Cells	5
Contigs*	3,035
Max Contig (MB)	221,849,541
Mean Contig (MB)	800,422.18
Contig N50 (MB)	68,155,215
Contig N90 (MB)	68,155,215
Total Contig Length	2,429,281,302
Assembly GC %	34.82
Scaffolds†	26
Max Scaffold (MB)	127,787,289
Mean Scaffold (MB)	88,339,354.80
Scaffold N50	107,960,188
Scaffold N90	62,911,544
Number scaffolded contigs	34
Total Scaffold Length (MB)	2,296,823,225
Number of genes	85,371
Repeat sequences (%)	70.6

[†]Scaffold metrics reported are after manual scaffolding

Table S2. Newly collected samples include 141 Florida cotton accessions (excluding Mound Key cotton) and 158 Yucatán cotton accessions. SiteName refers to the actual collection location, while SiteCode provides an abbreviation used to represent each site. Individuals collected from the same site are distinguished by a numeric suffix appended to the site name. Average sequencing depth was calculated based on reads mapped to the reference genome TEX2094. The corresponding NCBI accession numbers indicate where the sequencing data have been deposited. For conservation purposes, the precise GPS coordinates of collection sites are not included.

Species	Region	SiteName	SiteCode	Individual	Sequence depth	NCBI accession
G. hirsutum	Florida	Crane Point Hammock, Marathon Key (“C.P.H”)	FL_CPH	AD1_FL_CPH_1	19.86	SRR32570658
G. hirsutum	Florida	Crane Point Hammock, Marathon Key (“C.P.H”)	FL_CPH	AD1_FL_CPH_10	12.27	SRR32570657
G. hirsutum	Florida	Crane Point Hammock, Marathon Key (“C.P.H”)	FL_CPH	AD1_FL_CPH_2	14.9	SRR32570605
G. hirsutum	Florida	Crane Point Hammock, Marathon Key (“C.P.H”)	FL_CPH	AD1_FL_CPH_3	17.32	SRR32570594
G. hirsutum	Florida	Crane Point Hammock, Marathon Key (“C.P.H”)	FL_CPH	AD1_FL_CPH_4	16.95	SRR32570583
G. hirsutum	Florida	Crane Point Hammock, Marathon Key (“C.P.H”)	FL_CPH	AD1_FL_CPH_5	13.78	SRR32570572
G. hirsutum	Florida	Crane Point Hammock, Marathon Key (“C.P.H”)	FL_CPH	AD1_FL_CPH_6	15.96	SRR32570561
G. hirsutum	Florida	Crane Point Hammock, Marathon Key (“C.P.H”)	FL_CPH	AD1_FL_CPH_7	12.58	SRR32570550
G. hirsutum	Florida	Crane Point Hammock, Marathon Key (“C.P.H”)	FL_CPH	AD1_FL_CPH_8	12.66	SRR32570539
G. hirsutum	Florida	Crane Point Hammock, Marathon Key (“C.P.H”)	FL_CPH	AD1_FL_CPH_9	12.24	SRR32570528
G. hirsutum	Florida	Coastal Prairie Trail, Everglades (“C.P.T.”)	FL_CPT	AD1_FL_CPT_1	22.74	SRR32570656
G. hirsutum	Florida	Coastal Prairie Trail, Everglades (“C.P.T.”)	FL_CPT	AD1_FL_CPT_10	23.26	SRR32570645
G. hirsutum	Florida	Coastal Prairie Trail, Everglades (“C.P.T.”)	FL_CPT	AD1_FL_CPT_11	26.2	SRR32570634
G. hirsutum	Florida	Coastal Prairie Trail, Everglades (“C.P.T.”)	FL_CPT	AD1_FL_CPT_12	20.7	SRR32570623
G. hirsutum	Florida	Coastal Prairie Trail, Everglades (“C.P.T.”)	FL_CPT	AD1_FL_CPT_13	21.27	SRR32570612
G. hirsutum	Florida	Coastal Prairie Trail, Everglades (“C.P.T.”)	FL_CPT	AD1_FL_CPT_14	21.01	SRR32570610
G. hirsutum	Florida	Coastal Prairie Trail, Everglades (“C.P.T.”)	FL_CPT	AD1_FL_CPT_15	24.48	SRR32570609
G. hirsutum	Florida	Coastal Prairie Trail, Everglades (“C.P.T.”)	FL_CPT	AD1_FL_CPT_16	21.93	SRR32570608
G. hirsutum	Florida	Coastal Prairie Trail, Everglades (“C.P.T.”)	FL_CPT	AD1_FL_CPT_17	37.25	SRR32570607
G. hirsutum	Florida	Coastal Prairie Trail, Everglades (“C.P.T.”)	FL_CPT	AD1_FL_CPT_18	22.25	SRR32570606
G. hirsutum	Florida	Coastal Prairie Trail, Everglades (“C.P.T.”)	FL_CPT	AD1_FL_CPT_19	23.36	SRR32570604
G. hirsutum	Florida	Coastal Prairie Trail, Everglades (“C.P.T.”)	FL_CPT	AD1_FL_CPT_2	21.72	SRR32570603
G. hirsutum	Florida	Coastal Prairie Trail, Everglades (“C.P.T.”)	FL_CPT	AD1_FL_CPT_20	30.19	SRR32570602
G. hirsutum	Florida	Coastal Prairie Trail, Everglades (“C.P.T.”)	FL_CPT	AD1_FL_CPT_21	27.07	SRR32570601
G. hirsutum	Florida	Coastal Prairie Trail, Everglades (“C.P.T.”)	FL_CPT	AD1_FL_CPT_22	30.06	SRR32570600
G. hirsutum	Florida	Coastal Prairie Trail, Everglades (“C.P.T.”)	FL_CPT	AD1_FL_CPT_23	17.43	SRR32570599
G. hirsutum	Florida	Coastal Prairie Trail, Everglades (“C.P.T.”)	FL_CPT	AD1_FL_CPT_24	23.74	SRR32570598
G. hirsutum	Florida	Coastal Prairie Trail, Everglades (“C.P.T.”)	FL_CPT	AD1_FL_CPT_25	25.49	SRR32570597
G. hirsutum	Florida	Coastal Prairie Trail, Everglades (“C.P.T.”)	FL_CPT	AD1_FL_CPT_26	26.76	SRR32570596
G. hirsutum	Florida	Coastal Prairie Trail, Everglades (“C.P.T.”)	FL_CPT	AD1_FL_CPT_27	18.27	SRR32570595
G. hirsutum	Florida	Coastal Prairie Trail, Everglades (“C.P.T.”)	FL_CPT	AD1_FL_CPT_28	28.24	SRR32570593
G. hirsutum	Florida	Coastal Prairie Trail, Everglades (“C.P.T.”)	FL_CPT	AD1_FL_CPT_29	23.96	SRR32570592
G. hirsutum	Florida	Coastal Prairie Trail, Everglades (“C.P.T.”)	FL_CPT	AD1_FL_CPT_3	19.97	SRR32570591
G. hirsutum	Florida	Coastal Prairie Trail, Everglades (“C.P.T.”)	FL_CPT	AD1_FL_CPT_30	20.84	SRR32570590
G. hirsutum	Florida	Coastal Prairie Trail, Everglades (“C.P.T.”)	FL_CPT	AD1_FL_CPT_4	23.57	SRR32570589
G. hirsutum	Florida	Coastal Prairie Trail, Everglades (“C.P.T.”)	FL_CPT	AD1_FL_CPT_5	21.05	SRR32570588
G. hirsutum	Florida	Coastal Prairie Trail, Everglades (“C.P.T.”)	FL_CPT	AD1_FL_CPT_6	22.76	SRR32570587
G. hirsutum	Florida	Coastal Prairie Trail, Everglades (“C.P.T.”)	FL_CPT	AD1_FL_CPT_7	19.09	SRR32570586
G. hirsutum	Florida	Coastal Prairie Trail, Everglades (“C.P.T.”)	FL_CPT	AD1_FL_CPT_8	22.1	SRR32570585
G. hirsutum	Florida	Coastal Prairie Trail, Everglades (“C.P.T.”)	FL_CPT	AD1_FL_CPT_9	21.2	SRR32570584
G. hirsutum	Florida	Flamingo Maintenance Yard (“FMY”)	FL_FMY	AD1_FL_FMY_1	25.65	SRR32570582
G. hirsutum	Florida	Flamingo Maintenance Yard (“FMY”)	FL_FMY	AD1_FL_FMY_10	21.63	SRR32570581
G. hirsutum	Florida	Flamingo Maintenance Yard (“FMY”)	FL_FMY	AD1_FL_FMY_11	30.48	SRR32570580
G. hirsutum	Florida	Flamingo Maintenance Yard (“FMY”)	FL_FMY	AD1_FL_FMY_12	20.95	SRR32570579
G. hirsutum	Florida	Flamingo Maintenance Yard (“FMY”)	FL_FMY	AD1_FL_FMY_13	28.95	SRR32570578
G. hirsutum	Florida	Flamingo Maintenance Yard (“FMY”)	FL_FMY	AD1_FL_FMY_14	21.82	SRR32570577
G. hirsutum	Florida	Flamingo Maintenance Yard (“FMY”)	FL_FMY	AD1_FL_FMY_15	22.58	SRR32570576
G. hirsutum	Florida	Flamingo Maintenance Yard (“FMY”)	FL_FMY	AD1_FL_FMY_2	23.21	SRR32570575
G. hirsutum	Florida	Flamingo Maintenance Yard (“FMY”)	FL_FMY	AD1_FL_FMY_3	21.24	SRR32570574

G. hirsutum	Florida	Flamingo Maintenance Yard (“FMY”)	FL_FMY	AD1_FL_FMY_4	22.61	SRR32570573
G. hirsutum	Florida	Flamingo Maintenance Yard (“FMY”)	FL_FMY	AD1_FL_FMY_5	20.66	SRR32570571
G. hirsutum	Florida	Flamingo Maintenance Yard (“FMY”)	FL_FMY	AD1_FL_FMY_6	20.57	SRR32570570
G. hirsutum	Florida	Flamingo Maintenance Yard (“FMY”)	FL_FMY	AD1_FL_FMY_7	23.24	SRR32570569
G. hirsutum	Florida	Flamingo Maintenance Yard (“FMY”)	FL_FMY	AD1_FL_FMY_8	24.06	SRR32570568
G. hirsutum	Florida	Flamingo Maintenance Yard (“FMY”)	FL_FMY	AD1_FL_FMY_9	14.99	SRR32570567
G. hirsutum	Florida	Neal Preserve (“N.P.”)	FL_NP	AD1_FL_NP_1	21.54	SRR32570566
G. hirsutum	Florida	Neal Preserve (“N.P.”)	FL_NP	AD1_FL_NP_10	22.27	SRR32570565
G. hirsutum	Florida	Neal Preserve (“N.P.”)	FL_NP	AD1_FL_NP_11	21.28	SRR32570564
G. hirsutum	Florida	Neal Preserve (“N.P.”)	FL_NP	AD1_FL_NP_12	23.11	SRR32570563
G. hirsutum	Florida	Neal Preserve (“N.P.”)	FL_NP	AD1_FL_NP_13	22.31	SRR32570562
G. hirsutum	Florida	Neal Preserve (“N.P.”)	FL_NP	AD1_FL_NP_14	22.29	SRR32570560
G. hirsutum	Florida	Neal Preserve (“N.P.”)	FL_NP	AD1_FL_NP_15	20.23	SRR32570559
G. hirsutum	Florida	Neal Preserve (“N.P.”)	FL_NP	AD1_FL_NP_16	22.01	SRR32570558
G. hirsutum	Florida	Neal Preserve (“N.P.”)	FL_NP	AD1_FL_NP_17	24.77	SRR32570557
G. hirsutum	Florida	Neal Preserve (“N.P.”)	FL_NP	AD1_FL_NP_18	23.74	SRR32570556
G. hirsutum	Florida	Neal Preserve (“N.P.”)	FL_NP	AD1_FL_NP_2	22.42	SRR32570555
G. hirsutum	Florida	Neal Preserve (“N.P.”)	FL_NP	AD1_FL_NP_3	20.68	SRR32570554
G. hirsutum	Florida	Neal Preserve (“N.P.”)	FL_NP	AD1_FL_NP_4	22.66	SRR32570553
G. hirsutum	Florida	Neal Preserve (“N.P.”)	FL_NP	AD1_FL_NP_5	19.44	SRR32570552
G. hirsutum	Florida	Neal Preserve (“N.P.”)	FL_NP	AD1_FL_NP_6	22.49	SRR32570551
G. hirsutum	Florida	Neal Preserve (“N.P.”)	FL_NP	AD1_FL_NP_7	19.99	SRR32570549
G. hirsutum	Florida	Neal Preserve (“N.P.”)	FL_NP	AD1_FL_NP_8	21.04	SRR32570548
G. hirsutum	Florida	Neal Preserve (“N.P.”)	FL_NP	AD1_FL_NP_9	22.39	SRR32570547
G. hirsutum	Florida	Ocean Point Building 2, Tavernier Key	FL_OPB2	AD1_FL_OPB2_1	14.53	SRR32570546
G. hirsutum	Florida	Ocean Point Building 2, Tavernier Key	FL_OPB2	AD1_FL_OPB2_10	23.41	SRR32570545
G. hirsutum	Florida	Ocean Point Building 2, Tavernier Key	FL_OPB2	AD1_FL_OPB2_11	23.28	SRR32570544
G. hirsutum	Florida	Ocean Point Building 2, Tavernier Key	FL_OPB2	AD1_FL_OPB2_2	18.36	SRR32570543
G. hirsutum	Florida	Ocean Point Building 2, Tavernier Key	FL_OPB2	AD1_FL_OPB2_26	23.08	SRR32570542
G. hirsutum	Florida	Ocean Point Building 2, Tavernier Key	FL_OPB2	AD1_FL_OPB2_3	21.52	SRR32570541
G. hirsutum	Florida	Ocean Point Building 2, Tavernier Key	FL_OPB2	AD1_FL_OPB2_4	14.8	SRR32570540
G. hirsutum	Florida	Ocean Point Building 2, Tavernier Key	FL_OPB2	AD1_FL_OPB2_5	21.24	SRR32570538
G. hirsutum	Florida	Ocean Point Building 2, Tavernier Key	FL_OPB2	AD1_FL_OPB2_6	22.19	SRR32570537
G. hirsutum	Florida	Ocean Point Building 2, Tavernier Key	FL_OPB2	AD1_FL_OPB2_7	21.1	SRR32570536
G. hirsutum	Florida	Ocean Point Building 2, Tavernier Key	FL_OPB2	AD1_FL_OPB2_8	22.25	SRR32570535
G. hirsutum	Florida	Ocean Point Building 2, Tavernier Key	FL_OPB2	AD1_FL_OPB2_9	19.26	SRR32570534
G. hirsutum	Florida	Ocean Point Building 4, Tavernier Key	FL_OPB4	AD1_FL_OPB4_12	23.12	SRR32570533
G. hirsutum	Florida	Ocean Point Building 4, Tavernier Key	FL_OPB4	AD1_FL_OPB4_13	17.9	SRR32570532
G. hirsutum	Florida	Ocean Point Building 4, Tavernier Key	FL_OPB4	AD1_FL_OPB4_14	14.81	SRR32570531
G. hirsutum	Florida	Ocean Point Building 4, Tavernier Key	FL_OPB4	AD1_FL_OPB4_15	22.12	SRR32570530
G. hirsutum	Florida	Ocean Point Building 4, Tavernier Key	FL_OPB4	AD1_FL_OPB4_16	17.51	SRR32570529
G. hirsutum	Florida	Ocean Point Building 5, Tavernier Key	FL_OPB5	AD1_FL_OPB5_17	21.56	SRR32570527
G. hirsutum	Florida	Ocean Point Building 5, Tavernier Key	FL_OPB5	AD1_FL_OPB5_18	20.55	SRR32570526
G. hirsutum	Florida	Ocean Point Building 5, Tavernier Key	FL_OPB5	AD1_FL_OPB5_19	21.27	SRR32570525
G. hirsutum	Florida	Ocean Point Building 5, Tavernier Key	FL_OPB5	AD1_FL_OPB5_20	23.42	SRR32570524
G. hirsutum	Florida	Ocean Point Building 5, Tavernier Key	FL_OPB5	AD1_FL_OPB5_21	21.77	SRR32570523
G. hirsutum	Florida	Ocean Point Building 5, Tavernier Key	FL_OPB5	AD1_FL_OPB5_22	22.25	SRR32570522
G. hirsutum	Florida	Ocean Point Building 5, Tavernier Key	FL_OPB5	AD1_FL_OPB5_23	22.89	SRR32570521
G. hirsutum	Florida	Ocean Point Building 5, Tavernier Key	FL_OPB5	AD1_FL_OPB5_24	15.7	SRR32570520
G. hirsutum	Florida	Ocean Point Building 5, Tavernier Key	FL_OPB5	AD1_FL_OPB5_25	18.54	SRR32570519
G. hirsutum	Florida	Plantation Key	FL_PK	AD1_FL_PK_1	23.88	SRR32570518
G. hirsutum	Florida	Plantation Key	FL_PK	AD1_FL_PK_2	17.01	SRR32570655
G. hirsutum	Florida	Plantation Key	FL_PK	AD1_FL_PK_3	18.94	SRR32570654
G. hirsutum	Florida	Rowdy Bend Dumpster, Everglades	FL_RBD	AD1_FL_RBD_1	22.29	SRR32570653
G. hirsutum	Florida	Rowdy Bend Dumpster, Everglades	FL_RBD	AD1_FL_RBD_2	19.07	SRR32570652
G. hirsutum	Florida	Rowdy Bend Trail, Everglades	FL_RBT	AD1_FL_RBT_1	33.79	SRR32570651
G. hirsutum	Florida	Rowdy Bend Trail, Everglades	FL_RBT	AD1_FL_RBT_2	22.5	SRR32570650

G. hirsutum	Florida	Rowdy Bend Trail, Everglades	FL_RBT	AD1_FL_RBT_3	26.52	SRR32570649
G. hirsutum	Florida	Rowdy Bend Trail, Everglades	FL_RBT	AD1_FL_RBT_4	26.03	SRR32570648
G. hirsutum	Florida	Roadside North of Rowdy Bend turnoff	FL_RNRB	AD1_FL_RNRB_1	16.87	SRR32570647
G. hirsutum	Florida	Roadside North of Rowdy Bend turnoff	FL_RNRB	AD1_FL_RNRB_2	25.35	SRR32570646
G. hirsutum	Florida	Snappers Restaurant, Key Largo	FL_SR	AD1_FL_SR_1	17.44	SRR32570644
G. hirsutum	Florida	Snappers Restaurant, Key Largo	FL_SR	AD1_FL_SR_2	18.28	SRR32570643
G. hirsutum	Florida	Snappers Restaurant, Key Largo	FL_SR	AD1_FL_SR_3	15.37	SRR32570642
G. hirsutum	Florida	Snappers Restaurant, Key Largo	FL_SR	AD1_FL_SR_4	34.91	SRR32570641
G. hirsutum	Florida	Snappers Restaurant, Key Largo	FL_SR	AD1_FL_SR_5	22.06	SRR32570640
G. hirsutum	Florida	Snappers Restaurant, Key Largo	FL_SR	AD1_FL_SR_6	20.25	SRR32570639
G. hirsutum	Florida	Terra Ciea ("T.C.")	FL_TC	AD1_FL_TC_1	22.63	SRR32570638
G. hirsutum	Florida	Terra Ciea ("T.C.")	FL_TC	AD1_FL_TC_10	21.43	SRR32570637
G. hirsutum	Florida	Terra Ciea ("T.C.")	FL_TC	AD1_FL_TC_11	19.78	SRR32570636
G. hirsutum	Florida	Terra Ciea ("T.C.")	FL_TC	AD1_FL_TC_12	23.03	SRR32570635
G. hirsutum	Florida	Terra Ciea ("T.C.")	FL_TC	AD1_FL_TC_13	17.68	SRR32570633
G. hirsutum	Florida	Terra Ciea ("T.C.")	FL_TC	AD1_FL_TC_14	22.02	SRR32570632
G. hirsutum	Florida	Terra Ciea ("T.C.")	FL_TC	AD1_FL_TC_15	22.62	SRR32570631
G. hirsutum	Florida	Terra Ciea ("T.C.")	FL_TC	AD1_FL_TC_16	22.73	SRR32570630
G. hirsutum	Florida	Terra Ciea ("T.C.")	FL_TC	AD1_FL_TC_17	22.31	SRR32570629
G. hirsutum	Florida	Terra Ciea ("T.C.")	FL_TC	AD1_FL_TC_18	22.76	SRR32570628
G. hirsutum	Florida	Terra Ciea ("T.C.")	FL_TC	AD1_FL_TC_19	19.24	SRR32570627
G. hirsutum	Florida	Terra Ciea ("T.C.")	FL_TC	AD1_FL_TC_2	20.98	SRR32570626
G. hirsutum	Florida	Terra Ciea ("T.C.")	FL_TC	AD1_FL_TC_20	21.1	SRR32570625
G. hirsutum	Florida	Terra Ciea ("T.C.")	FL_TC	AD1_FL_TC_3	22.64	SRR32570624
G. hirsutum	Florida	Terra Ciea ("T.C.")	FL_TC	AD1_FL_TC_4	18.02	SRR32570622
G. hirsutum	Florida	Terra Ciea ("T.C.")	FL_TC	AD1_FL_TC_5	21.12	SRR32570621
G. hirsutum	Florida	Terra Ciea ("T.C.")	FL_TC	AD1_FL_TC_6	23.15	SRR32570620
G. hirsutum	Florida	Terra Ciea ("T.C.")	FL_TC	AD1_FL_TC_7	18.82	SRR32570619
G. hirsutum	Florida	Terra Ciea ("T.C.")	FL_TC	AD1_FL_TC_8	21.3	SRR32570618
G. hirsutum	Florida	Terra Ciea ("T.C.")	FL_TC	AD1_FL_TC_9	21.47	SRR32570617
G. hirsutum	Florida	Vaca Key Peachtree Avenue	FL_VKPA	AD1_FL_VKPA_1	16.49	SRR32570616
G. hirsutum	Florida	Vaca Key Peachtree Avenue	FL_VKPA	AD1_FL_VKPA_2	25.67	SRR32570615
G. hirsutum	Florida	Vaca Key Peachtree Avenue	FL_VKPA	AD1_FL_VKPA_3	18.61	SRR32570614
G. hirsutum	Florida	Vaca Key Peachtree Avenue	FL_VKPA	AD1_FL_VKPA_4	21.39	SRR32570613
G. hirsutum	Florida	Vaca Key Peachtree Avenue	FL_VKPA	AD1_FL_VKPA_5	25.33	SRR32570611
G. hirsutum	Yucatan	Celestún Site: Consorcio	YUC_CeCo	AD1_YUC_CeCo_M10	26.71	SRR35548024
G. hirsutum	Yucatan	Celestún Site: Consorcio	YUC_CeCo	AD1_YUC_CeCo_M11	33.02	SRR35548023
G. hirsutum	Yucatan	Celestún Site: Consorcio	YUC_CeCo	AD1_YUC_CeCo_M2	30.14	SRR35547954
G. hirsutum	Yucatan	Celestún Site: Consorcio	YUC_CeCo	AD1_YUC_CeCo_M3	31.58	SRR35547943
G. hirsutum	Yucatan	Celestún Site: Consorcio	YUC_CeCo	AD1_YUC_CeCo_M4	26.54	SRR35547932
G. hirsutum	Yucatan	Celestún Site: Consorcio	YUC_CeCo	AD1_YUC_CeCo_M5	25.76	SRR35547921
G. hirsutum	Yucatan	Celestún Site: Consorcio	YUC_CeCo	AD1_YUC_CeCo_M6	25.82	SRR35548018
G. hirsutum	Yucatan	Celestún Site: Consorcio	YUC_CeCo	AD1_YUC_CeCo_M7	28.96	SRR35548007
G. hirsutum	Yucatan	Celestún Site: Consorcio	YUC_CeCo	AD1_YUC_CeCo_M8	27.74	SRR35547996
G. hirsutum	Yucatan	Celestún Site: Consorcio	YUC_CeCo	AD1_YUC_CeCo_M9	26.69	SRR35547985
G. hirsutum	Yucatan	Celestún Site: Consorcio	YUC_CeCo	AD1_YUC_CeCo_S51	24.69	SRR35548022
G. hirsutum	Yucatan	Celestún Site: Consorcio	YUC_CeCo	AD1_YUC_CeCo_S52	27.1	SRR35548051
G. hirsutum	Yucatan	Celestún Site: Consorcio	YUC_CeCo	AD1_YUC_CeCo_W1	25.68	SRR35548040
G. hirsutum	Yucatan	Celestún Site: Consorcio	YUC_CeCo	AD1_YUC_CeCo_W2	24.98	SRR35548029
G. hirsutum	Yucatan	Celestún Site: Consorcio	YUC_CeCo	AD1_YUC_CeCo_W3	31.28	SRR35547978
G. hirsutum	Yucatan	Celestún Site: Consorcio	YUC_CeCo	AD1_YUC_CeCo_W4	25.98	SRR35547967
G. hirsutum	Yucatan	Celestún Site: Consorcio	YUC_CeCo	AD1_YUC_CeCo_W5	27.25	SRR35547958
G. hirsutum	Yucatan	Celestún Site: Consorcio	YUC_CeCo	AD1_YUC_CeCo_W6	28.71	SRR35547957
G. hirsutum	Yucatan	Celestún Site: Consorcio	YUC_CeCo	AD1_YUC_CeCo_W7	28.89	SRR35547956
G. hirsutum	Yucatan	Celestún Site: Consorcio	YUC_CeCo	AD1_YUC_CeCo_W8	30.54	SRR35547955
G. hirsutum	Yucatan	Centro Site: Dos torres	YUC_CeDo	AD1_YUC_CeDo_H22	26.51	SRR35547953
G. hirsutum	Yucatan	Centro Site: Dos torres	YUC_CeDo	AD1_YUC_CeDo_M2	30.32	SRR35547952

G. hirsutum	Yucatan	Centro Site: Dos torres	YUC_CeDo	AD1_YUC_CeDo_M3	25.75	SRR35547951
G. hirsutum	Yucatan	Centro Site: Dos torres	YUC_CeDo	AD1_YUC_CeDo_M4	25.94	SRR35547950
G. hirsutum	Yucatan	Centro Site: Dos torres	YUC_CeDo	AD1_YUC_CeDo_M6	27.26	SRR35547949
G. hirsutum	Yucatan	Centro Site: Dos torres	YUC_CeDo	AD1_YUC_CeDo_M7	28.19	SRR35547948
G. hirsutum	Yucatan	Centro Site: Dos torres	YUC_CeDo	AD1_YUC_CeDo_S51	27.26	SRR35547947
G. hirsutum	Yucatan	Centro Site: Dos torres	YUC_CeDo	AD1_YUC_CeDo_S53	22.1	SRR35547946
G. hirsutum	Yucatan	Centro Site: Dos torres	YUC_CeDo	AD1_YUC_CeDo_S54	24.34	SRR35547945
G. hirsutum	Yucatan	Centro Site: Dos torres	YUC_CeDo	AD1_YUC_CeDo_W1	26.79	SRR35547941
G. hirsutum	Yucatan	Centro Site: Dos torres	YUC_CeDo	AD1_YUC_CeDo_W10	26.82	SRR35547944
G. hirsutum	Yucatan	Centro Site: Dos torres	YUC_CeDo	AD1_YUC_CeDo_W11	24.76	SRR35547942
G. hirsutum	Yucatan	Centro Site: Dos torres	YUC_CeDo	AD1_YUC_CeDo_W2	30.34	SRR35547940
G. hirsutum	Yucatan	Centro Site: Dos torres	YUC_CeDo	AD1_YUC_CeDo_W3	31.4	SRR35547939
G. hirsutum	Yucatan	Centro Site: Dos torres	YUC_CeDo	AD1_YUC_CeDo_W4	27.94	SRR35547938
G. hirsutum	Yucatan	Centro Site: Dos torres	YUC_CeDo	AD1_YUC_CeDo_W5	21.97	SRR35547937
G. hirsutum	Yucatan	Centro Site: Dos torres	YUC_CeDo	AD1_YUC_CeDo_W6	21.43	SRR35547936
G. hirsutum	Yucatan	Centro Site: Dos torres	YUC_CeDo	AD1_YUC_CeDo_W7	23.63	SRR35547935
G. hirsutum	Yucatan	Centro Site: Dos torres	YUC_CeDo	AD1_YUC_CeDo_W8	22.76	SRR35547934
G. hirsutum	Yucatan	Centro Site: Dos torres	YUC_CeDo	AD1_YUC_CeDo_W9	22.81	SRR35547933
G. hirsutum	Yucatan	Celestún Site: Drilos	YUC_CeDr	AD1_YUC_CeDr_DR12	26.36	SRR35547931
G. hirsutum	Yucatan	Celestún Site: Drilos	YUC_CeDr	AD1_YUC_CeDr_DR13	24.13	SRR35547930
G. hirsutum	Yucatan	Celestún Site: Drilos	YUC_CeDr	AD1_YUC_CeDr_M2	23.83	SRR35547929
G. hirsutum	Yucatan	Celestún Site: Drilos	YUC_CeDr	AD1_YUC_CeDr_M3	27.86	SRR35547928
G. hirsutum	Yucatan	Celestún Site: Drilos	YUC_CeDr	AD1_YUC_CeDr_M4	21.55	SRR35547927
G. hirsutum	Yucatan	Celestún Site: Drilos	YUC_CeDr	AD1_YUC_CeDr_M5	25.78	SRR35547926
G. hirsutum	Yucatan	Celestún Site: Drilos	YUC_CeDr	AD1_YUC_CeDr_M6	25.46	SRR35547925
G. hirsutum	Yucatan	Celestún Site: Drilos	YUC_CeDr	AD1_YUC_CeDr_M9	26.7	SRR35547924
G. hirsutum	Yucatan	Celestún Site: Drilos	YUC_CeDr	AD1_YUC_CeDr_W1	23.53	SRR35547920
G. hirsutum	Yucatan	Celestún Site: Drilos	YUC_CeDr	AD1_YUC_CeDr_W10	22.84	SRR35547923
G. hirsutum	Yucatan	Celestún Site: Drilos	YUC_CeDr	AD1_YUC_CeDr_W11	29.74	SRR35547922
G. hirsutum	Yucatan	Celestún Site: Drilos	YUC_CeDr	AD1_YUC_CeDr_W12	22.72	SRR35547919
G. hirsutum	Yucatan	Celestún Site: Drilos	YUC_CeDr	AD1_YUC_CeDr_W13	25.22	SRR35547918
G. hirsutum	Yucatan	Celestún Site: Drilos	YUC_CeDr	AD1_YUC_CeDr_W2	26.76	SRR35547917
G. hirsutum	Yucatan	Celestún Site: Drilos	YUC_CeDr	AD1_YUC_CeDr_W3	22.71	SRR35547916
G. hirsutum	Yucatan	Celestún Site: Drilos	YUC_CeDr	AD1_YUC_CeDr_W4	26.2	SRR35547915
G. hirsutum	Yucatan	Celestún Site: Drilos	YUC_CeDr	AD1_YUC_CeDr_W5	27.59	SRR35547914
G. hirsutum	Yucatan	Celestún Site: Drilos	YUC_CeDr	AD1_YUC_CeDr_W6	32.99	SRR35547913
G. hirsutum	Yucatan	Celestún Site: Drilos	YUC_CeDr	AD1_YUC_CeDr_W7	30.07	SRR35548020
G. hirsutum	Yucatan	Celestún Site: Drilos	YUC_CeDr	AD1_YUC_CeDr_W8	25.72	SRR35548019
G. hirsutum	Yucatan	Celestún Site: Drilos	YUC_CeDr	AD1_YUC_CeDr_W9	23.53	SRR35548017
G. hirsutum	Yucatan	Centro Site: Pretropical	YUC_CePr	AD1_YUC_CePr_H23	28.14	SRR35548016
G. hirsutum	Yucatan	Centro Site: Pretropical	YUC_CePr	AD1_YUC_CePr_M1	27.33	SRR35548015
G. hirsutum	Yucatan	Centro Site: Pretropical	YUC_CePr	AD1_YUC_CePr_M3	25.93	SRR35548014
G. hirsutum	Yucatan	Centro Site: Pretropical	YUC_CePr	AD1_YUC_CePr_M5	30.57	SRR35548013
G. hirsutum	Yucatan	Centro Site: Pretropical	YUC_CePr	AD1_YUC_CePr_M6	26	SRR35548012
G. hirsutum	Yucatan	Centro Site: Pretropical	YUC_CePr	AD1_YUC_CePr_M7	25.84	SRR35548011
G. hirsutum	Yucatan	Centro Site: Pretropical	YUC_CePr	AD1_YUC_CePr_S51	24.1	SRR35548010
G. hirsutum	Yucatan	Centro Site: Pretropical	YUC_CePr	AD1_YUC_CePr_S52	25.87	SRR35548009
G. hirsutum	Yucatan	Centro Site: Pretropical	YUC_CePr	AD1_YUC_CePr_S61	21.81	SRR35548008
G. hirsutum	Yucatan	Centro Site: Pretropical	YUC_CePr	AD1_YUC_CePr_S62	24.13	SRR35548006
G. hirsutum	Yucatan	Centro Site: Pretropical	YUC_CePr	AD1_YUC_CePr_S63	24.64	SRR35548005
G. hirsutum	Yucatan	Centro Site: Pretropical	YUC_CePr	AD1_YUC_CePr_W1	25.63	SRR35548004
G. hirsutum	Yucatan	Centro Site: Pretropical	YUC_CePr	AD1_YUC_CePr_W2	26.09	SRR35548003
G. hirsutum	Yucatan	Centro Site: Pretropical	YUC_CePr	AD1_YUC_CePr_W3	28.33	SRR35548002
G. hirsutum	Yucatan	Centro Site: Pretropical	YUC_CePr	AD1_YUC_CePr_W4	20.73	SRR35548001
G. hirsutum	Yucatan	Centro Site: Pretropical	YUC_CePr	AD1_YUC_CePr_W5	25.4	SRR35548000
G. hirsutum	Yucatan	Centro Site: Pretropical	YUC_CePr	AD1_YUC_CePr_W6	24.33	SRR35547999
G. hirsutum	Yucatan	Río lagartos Site: Cangrejos	YUC_RiCa	AD1_YUC_RiCa_B10	29.77	SRR35547998

G. hirsutum	Yucatan	Río lagartos Site: Cangrejos	YUC_RiCa	AD1_YUC_RiCa_M10	24.45	SRR35547997
G. hirsutum	Yucatan	Río lagartos Site: Cangrejos	YUC_RiCa	AD1_YUC_RiCa_M11	24.36	SRR35547995
G. hirsutum	Yucatan	Río lagartos Site: Cangrejos	YUC_RiCa	AD1_YUC_RiCa_M12	23.46	SRR35547994
G. hirsutum	Yucatan	Río lagartos Site: Cangrejos	YUC_RiCa	AD1_YUC_RiCa_M13	25.62	SRR35547993
G. hirsutum	Yucatan	Río lagartos Site: Cangrejos	YUC_RiCa	AD1_YUC_RiCa_M14	25.38	SRR35547992
G. hirsutum	Yucatan	Río lagartos Site: Cangrejos	YUC_RiCa	AD1_YUC_RiCa_M2	26.14	SRR35547991
G. hirsutum	Yucatan	Río lagartos Site: Cangrejos	YUC_RiCa	AD1_YUC_RiCa_M3	26.59	SRR35547990
G. hirsutum	Yucatan	Río lagartos Site: Cangrejos	YUC_RiCa	AD1_YUC_RiCa_W1	25.6	SRR35547987
G. hirsutum	Yucatan	Río lagartos Site: Cangrejos	YUC_RiCa	AD1_YUC_RiCa_W10	25.13	SRR35547989
G. hirsutum	Yucatan	Río lagartos Site: Cangrejos	YUC_RiCa	AD1_YUC_RiCa_W11	31.66	SRR35547988
G. hirsutum	Yucatan	Río lagartos Site: Cangrejos	YUC_RiCa	AD1_YUC_RiCa_W12	27.7	SRR35547986
G. hirsutum	Yucatan	Río lagartos Site: Cangrejos	YUC_RiCa	AD1_YUC_RiCa_W2	13.33	SRR35547912
G. hirsutum	Yucatan	Río lagartos Site: Cangrejos	YUC_RiCa	AD1_YUC_RiCa_W3	19.04	SRR35547911
G. hirsutum	Yucatan	Río lagartos Site: Cangrejos	YUC_RiCa	AD1_YUC_RiCa_W4	21.03	SRR35547910
G. hirsutum	Yucatan	Río lagartos Site: Cangrejos	YUC_RiCa	AD1_YUC_RiCa_W5	22.32	SRR35547909
G. hirsutum	Yucatan	Río lagartos Site: Cangrejos	YUC_RiCa	AD1_YUC_RiCa_W6	25.33	SRR35547908
G. hirsutum	Yucatan	Río lagartos Site: Cangrejos	YUC_RiCa	AD1_YUC_RiCa_W7	26.81	SRR35547907
G. hirsutum	Yucatan	Río lagartos Site: Cangrejos	YUC_RiCa	AD1_YUC_RiCa_W8	28.52	SRR35547906
G. hirsutum	Yucatan	Río lagartos Site: Cangrejos	YUC_RiCa	AD1_YUC_RiCa_W9	26.35	SRR35547905
G. hirsutum	Yucatan	Río lagartos Site: Chorlitos	YUC_RiCh	AD1_YUC_RiCh_B13	30.64	SRR35547904
G. hirsutum	Yucatan	Río lagartos Site: Chorlitos	YUC_RiCh	AD1_YUC_RiCh_B14	30.03	SRR35547902
G. hirsutum	Yucatan	Río lagartos Site: Chorlitos	YUC_RiCh	AD1_YUC_RiCh_B5	28.36	SRR35548021
G. hirsutum	Yucatan	Río lagartos Site: Chorlitos	YUC_RiCh	AD1_YUC_RiCh_B8	35.26	SRR35547903
G. hirsutum	Yucatan	Río lagartos Site: Chorlitos	YUC_RiCh	AD1_YUC_RiCh_CT9	32.23	SRR35548059
G. hirsutum	Yucatan	Río lagartos Site: Chorlitos	YUC_RiCh	AD1_YUC_RiCh_M1	30.36	SRR35548057
G. hirsutum	Yucatan	Río lagartos Site: Chorlitos	YUC_RiCh	AD1_YUC_RiCh_M11	30.82	SRR35548058
G. hirsutum	Yucatan	Río lagartos Site: Chorlitos	YUC_RiCh	AD1_YUC_RiCh_M2	29.53	SRR35548056
G. hirsutum	Yucatan	Río lagartos Site: Chorlitos	YUC_RiCh	AD1_YUC_RiCh_M4	28.23	SRR35548055
G. hirsutum	Yucatan	Río lagartos Site: Chorlitos	YUC_RiCh	AD1_YUC_RiCh_M5	30.13	SRR35548054
G. hirsutum	Yucatan	Río lagartos Site: Chorlitos	YUC_RiCh	AD1_YUC_RiCh_M6	24.36	SRR35548053
G. hirsutum	Yucatan	Río lagartos Site: Chorlitos	YUC_RiCh	AD1_YUC_RiCh_M8	31.42	SRR35548052
G. hirsutum	Yucatan	Río lagartos Site: Chorlitos	YUC_RiCh	AD1_YUC_RiCh_M9	30.03	SRR35548050
G. hirsutum	Yucatan	Río lagartos Site: Chorlitos	YUC_RiCh	AD1_YUC_RiCh_S51	32.36	SRR35548049
G. hirsutum	Yucatan	Río lagartos Site: Chorlitos	YUC_RiCh	AD1_YUC_RiCh_S52	30.19	SRR35548048
G. hirsutum	Yucatan	Río lagartos Site: Chorlitos	YUC_RiCh	AD1_YUC_RiCh_W1	28.33	SRR35548047
G. hirsutum	Yucatan	Río lagartos Site: Chorlitos	YUC_RiCh	AD1_YUC_RiCh_W2	28.59	SRR35548046
G. hirsutum	Yucatan	Río lagartos Site: Chorlitos	YUC_RiCh	AD1_YUC_RiCh_W3	30.92	SRR35548045
G. hirsutum	Yucatan	Río lagartos Site: Chorlitos	YUC_RiCh	AD1_YUC_RiCh_W4	29.73	SRR35548044
G. hirsutum	Yucatan	Río lagartos Site: Chorlitos	YUC_RiCh	AD1_YUC_RiCh_W5	25.82	SRR35548043
G. hirsutum	Yucatan	Sisal Site: Papatzul	YUC_SiPa	AD1_YUC_SiPa_M11	26.06	SRR35548042
G. hirsutum	Yucatan	Sisal Site: Papatzul	YUC_SiPa	AD1_YUC_SiPa_M12	23.75	SRR35548041
G. hirsutum	Yucatan	Sisal Site: Papatzul	YUC_SiPa	AD1_YUC_SiPa_M13	25.26	SRR35548039
G. hirsutum	Yucatan	Sisal Site: Papatzul	YUC_SiPa	AD1_YUC_SiPa_M21	29.84	SRR35548038
G. hirsutum	Yucatan	Sisal Site: Papatzul	YUC_SiPa	AD1_YUC_SiPa_M3	32.98	SRR35548037
G. hirsutum	Yucatan	Sisal Site: Papatzul	YUC_SiPa	AD1_YUC_SiPa_M4	32.38	SRR35548036
G. hirsutum	Yucatan	Sisal Site: Papatzul	YUC_SiPa	AD1_YUC_SiPa_M5	20.93	SRR35548035
G. hirsutum	Yucatan	Sisal Site: Papatzul	YUC_SiPa	AD1_YUC_SiPa_M6	31.73	SRR35548034
G. hirsutum	Yucatan	Sisal Site: Papatzul	YUC_SiPa	AD1_YUC_SiPa_M7	24.4	SRR35548033
G. hirsutum	Yucatan	Sisal Site: Papatzul	YUC_SiPa	AD1_YUC_SiPa_M8	28.24	SRR35548032
G. hirsutum	Yucatan	Sisal Site: Papatzul	YUC_SiPa	AD1_YUC_SiPa_M9	24.82	SRR35548031
G. hirsutum	Yucatan	Sisal Site: Papatzul	YUC_SiPa	AD1_YUC_SiPa_W1	35.27	SRR35548030
G. hirsutum	Yucatan	Sisal Site: Papatzul	YUC_SiPa	AD1_YUC_SiPa_W2	22.73	SRR35548028
G. hirsutum	Yucatan	Sisal Site: Papatzul	YUC_SiPa	AD1_YUC_SiPa_W3	34.67	SRR35548027
G. hirsutum	Yucatan	Sisal Site: Papatzul	YUC_SiPa	AD1_YUC_SiPa_W4	25.83	SRR35548026
G. hirsutum	Yucatan	Sisal Site: Papatzul	YUC_SiPa	AD1_YUC_SiPa_W5	26.05	SRR35548025
G. hirsutum	Yucatan	Sisal Site: Papatzul	YUC_SiPa	AD1_YUC_SiPa_W6	34.82	SRR35547984
G. hirsutum	Yucatan	Sisal Site: Papatzul	YUC_SiPa	AD1_YUC_SiPa_W7	31.81	SRR35547983

G. hirsutum	Yucatan	Sisal Site: Papatzul	YUC_SiPa	AD1_YUC_SiPa_W8	30.64	SRR35547982
G. hirsutum	Yucatan	Sisal Site: Papatzul	YUC_SiPa	AD1_YUC_SiPa_W9	26.73	SRR35547981
G. hirsutum	Yucatan	Sisal Site: Preseco	YUC_SiPr	AD1_YUC_SiPr_M1	31.95	SRR35547977
G. hirsutum	Yucatan	Sisal Site: Preseco	YUC_SiPr	AD1_YUC_SiPr_M10	32.47	SRR35547980
G. hirsutum	Yucatan	Sisal Site: Preseco	YUC_SiPr	AD1_YUC_SiPr_M11	32.42	SRR35547979
G. hirsutum	Yucatan	Sisal Site: Preseco	YUC_SiPr	AD1_YUC_SiPr_M20	29.55	SRR35547976
G. hirsutum	Yucatan	Sisal Site: Preseco	YUC_SiPr	AD1_YUC_SiPr_M21	24.59	SRR35547975
G. hirsutum	Yucatan	Sisal Site: Preseco	YUC_SiPr	AD1_YUC_SiPr_M23	29.17	SRR35547974
G. hirsutum	Yucatan	Sisal Site: Preseco	YUC_SiPr	AD1_YUC_SiPr_S51	30.74	SRR35547973
G. hirsutum	Yucatan	Sisal Site: Preseco	YUC_SiPr	AD1_YUC_SiPr_S52	30.54	SRR35547972
G. hirsutum	Yucatan	Sisal Site: Preseco	YUC_SiPr	AD1_YUC_SiPr_W1	33.57	SRR35547969
G. hirsutum	Yucatan	Sisal Site: Preseco	YUC_SiPr	AD1_YUC_SiPr_W10	29.81	SRR35547971
G. hirsutum	Yucatan	Sisal Site: Preseco	YUC_SiPr	AD1_YUC_SiPr_W11	30.32	SRR35547970
G. hirsutum	Yucatan	Sisal Site: Preseco	YUC_SiPr	AD1_YUC_SiPr_W12	24.08	SRR35547968
G. hirsutum	Yucatan	Sisal Site: Preseco	YUC_SiPr	AD1_YUC_SiPr_W2	33.65	SRR35547966
G. hirsutum	Yucatan	Sisal Site: Preseco	YUC_SiPr	AD1_YUC_SiPr_W3	31.15	SRR35547965
G. hirsutum	Yucatan	Sisal Site: Preseco	YUC_SiPr	AD1_YUC_SiPr_W4	28.6	SRR35547964
G. hirsutum	Yucatan	Sisal Site: Preseco	YUC_SiPr	AD1_YUC_SiPr_W5	31.94	SRR35547963
G. hirsutum	Yucatan	Sisal Site: Preseco	YUC_SiPr	AD1_YUC_SiPr_W6	30.09	SRR35547962
G. hirsutum	Yucatan	Sisal Site: Preseco	YUC_SiPr	AD1_YUC_SiPr_W7	29.18	SRR35547961
G. hirsutum	Yucatan	Sisal Site: Preseco	YUC_SiPr	AD1_YUC_SiPr_W8	27.93	SRR35547960
G. hirsutum	Yucatan	Sisal Site: Preseco	YUC_SiPr	AD1_YUC_SiPr_W9	23.61	SRR35547959

Table S3. *Gossypium hirsutum* samples included in this study and their average sequencing depth. LR1 and LR2 correspond to Landrace1 and Landrace2, respectively, as previously designated by Yuan et al. (2021) (20).

Species	Group	Population	Sample ID	Sequencing depth	NCBI accession	Source
G. hirsutum	Germplasm	Cultivar	AD1_Cultivar_B11SA2401	19.52	SRR6311735	Yuan et al
G. hirsutum	Germplasm	Cultivar	AD1_Cultivar_B11SA2192	19.81	SRR6311605	Yuan et al
G. hirsutum	Germplasm	Cultivar	AD1_Cultivar_B11SA1403	20	SRR6311680	Yuan et al
G. hirsutum	Germplasm	Cultivar	AD1_Cultivar_B11SA0825	20.72	SRR6311822	Yuan et al
G. hirsutum	Germplasm	Cultivar	AD1_Cultivar_B11SA2149	21.22	SRR6311606	Yuan et al
G. hirsutum	Germplasm	Cultivar	AD1_Cultivar_B11DIV106	21.29	SRR6311690	Yuan et al
G. hirsutum	Germplasm	Cultivar	AD1_Cultivar_B11SA0582	21.34	SRR6311710	Yuan et al
G. hirsutum	Germplasm	Cultivar	AD1_Cultivar_B11SA1427	21.59	SRR6311783	Yuan et al
G. hirsutum	Germplasm	Cultivar	AD1_Cultivar_B11SA1465	22.98	SRR6311779	Yuan et al
G. hirsutum	Germplasm	Cultivar	AD1_Cultivar_B11SA3748	23.68	SRR6311741	Yuan et al
G. hirsutum	Germplasm	LR1	AD1_LR1_B12TX2226	16.26	SRR6311807	Yuan et al
G. hirsutum	Germplasm	LR1	AD1_LR1_B12TX1578	19.16	SRR6311813	Yuan et al
G. hirsutum	Germplasm	LR1	AD1_LR1_B12TX2489	19.65	SRR6311749	Yuan et al
G. hirsutum	Germplasm	LR1	AD1_LR1_B12TX2487	20.58	SRR6311752	Yuan et al
G. hirsutum	Germplasm	LR1	AD1_LR1_B12TX1689	20.74	SRR6311762	Yuan et al
G. hirsutum	Germplasm	LR1	AD1_LR1_B12TX1635	21.03	SRR6311803	Yuan et al
G. hirsutum	Germplasm	LR1	AD1_LR1_B12TX2216	21.05	SRR6311806	Yuan et al
G. hirsutum	Germplasm	LR1	AD1_LR1_B12TX1935	22.07	SRR6311799	Yuan et al
G. hirsutum	Germplasm	LR1	AD1_LR1_B12TX2465	24.57	SRR6311600	Yuan et al
G. hirsutum	Germplasm	LR1	AD1_LR1_B12TX1592	24.87	SRR6311758	Yuan et al
G. hirsutum	Germplasm	LR2	AD1_LR2_B12TX0109	16.1	SRR6311544	Yuan et al
G. hirsutum	Germplasm	LR2	AD1_LR2_B12TX1102	18.49	SRR6311801	Yuan et al
G. hirsutum	Germplasm	LR2	AD1_LR2_B12TX1459	18.93	SRR6311768	Yuan et al
G. hirsutum	Germplasm	LR2	AD1_LR2_B12TX1721	19.6	SRR6311751	Yuan et al
G. hirsutum	Germplasm	LR2	AD1_LR2_B12TX0215	21.22	SRR6311873	Yuan et al
G. hirsutum	Germplasm	LR2	AD1_LR2_B12TX1981	22.14	SRR6311802	Yuan et al
G. hirsutum	Germplasm	LR2	AD1_LR2_B12TX1661	22.38	SRR6311747	Yuan et al
G. hirsutum	Germplasm	LR2	AD1_LR2_B12TX0487	22.55	SRR6311550	Yuan et al
G. hirsutum	Germplasm	LR2	AD1_LR2_B12TX0390	23.43	SRR6311805	Yuan et al
G. hirsutum	Germplasm	LR2	AD1_LR2_B12TX1111	24.43	SRR6311759	Yuan et al

Table S5. Selection the reference genome using site PubPlant (https://www.plabipd.de/pubplant_main.html), based on phylogeny (https://www.plabipd.de/pubplant_cladogram1.html) and published date.

No.	Family	Species	Accession	NCBI Accession Number
1	Malvaceae	*Gossypium hisutum*	TEX2094 v.2 ISU	This study
2	Fabaceae	*Medicago truncatula*	MtrunA17r5.0-ANR	NCBI GCF_003473485.1
3	Brassicaceae	*Arabidopsis thaliana*	TAIR10.1	NCBI GCF_000001735.4
4	Asteraceae	*Helianthus annuus*	HanXRQr2.0-SUNRISE	NCBI GCF_002127325.2
5	Poaceae	*Oryza sativa Japonica Group*	AGIS1.0	NCBI GCA_034140825.1
6	Bromeliaceae	*Ananas comosus*	ASM154086v1	NCBI GCF_001540865.1
7	Apiaceae	*Daucus carota*	DH1 v3.0	NCBI GCF_001625215.2
8	Poaceae	*Zea mays*	Zm-B73-REFERENCE-NAM-5.0	NCBI GCA_902167145.1
9	Solanaceae	*Solanum lycopersicum*	SLM_r2.1	NCBI GCF_036512215.1
10	Theaceae	*Camellia pitardii*	ASM5162307v1	NCBI GCA_051623075.1
11	Vitaceae	*Vitis davidii*	V112.hap2_v1.0	NCBI GCA_044588485.1
12	Salicaceae	*Salix dunnii*	FNU-M-1-Hap-a	NCBI GCA_040801865.1
13	Rutaceae	*Citrus sinensis*	DVS_A1.0	NCBI GCA_022201045.1
14	Malvaceae	*Hibiscus yunnanensis*	ASM4900470v1	NCBI GCA_049004705.1
15	Malvaceae	*Gossypium raimondii*	ASM2569854v1	NCBI GCA_025698545.1

Table S6. Results of ANOVA and Tukey HSD tests for statistically significant pairwise differences in genetic load compared with YUC-W.

Methods	diff	lwr	upr	p adj	model	comparison	significant
GERP++	-9172.208636	-10132.88296	-8211.534308	0	Additive	Cultivar vs YUC-W	**
GERP++	11968.68886	11008.01454	12929.36319	0	Additive	LR1 vs YUC-W	**
GERP++	-4846.848136	-5807.522463	-3886.173808	0	Additive	LR2 vs YUC-W	**
GERP++	13493.22496	12802.39852	14184.0514	0	Additive	GD vs YUC-W	**
GERP++	13312.34186	11980.54265	14644.14108	0	Additive	PR vs YUC-W	**
GERP++	11629.15045	11277.9328	11980.36809	0	Additive	FL vs YUC-W	**
GERP++	2092.204114	1558.537852	2625.870377	0	Additive	YUC-E vs YUC-W	**
GERP++	-2936.328102	-5133.317305	-739.3388988	0.001447485675	Recessive	Cultivar vs YUC-W	**
GERP++	17327.9599	15130.9707	19524.9491	0	Recessive	LR1 vs YUC-W	**
GERP++	1246.644898	-950.3443046	3443.634101	0.6677996355	Recessive	LR2 vs YUC-W	
GERP++	19455.34652	17875.47893	21035.21411	0	Recessive	GD vs YUC-W	**
GERP++	19230.4049	16184.68128	22276.12852	0	Recessive	PR vs YUC-W	**
GERP++	17155.81299	16352.60492	17959.02107	0	Recessive	FL vs YUC-W	**
GERP++	7969.331398	6748.8772	9189.785596	0	Recessive	YUC-E vs YUC-W	**
SIFT4G	-193.8887712	-239.892304	-147.8852383	0	Additive	Cultivar vs YUC-W	**
SIFT4G	623.2862288	577.282696	669.2897617	0	Additive	LR1 vs YUC-W	**
SIFT4G	-28.48877119	-74.49230402	17.51476165	0.560251174	Additive	LR2 vs YUC-W	
SIFT4G	623.5844431	590.5030376	656.6658486	0	Additive	GD vs YUC-W	**
SIFT4G	540.4237288	476.6482463	604.1992114	0	Additive	PR vs YUC-W	**
SIFT4G	525.7317107	508.9130533	542.5503682	0	Additive	FL vs YUC-W	**
SIFT4G	134.8049788	109.2494573	160.3605003	0	Additive	YUC-E vs YUC-W	**
SIFT4G	249.7614407	59.70818854	439.8146928	0.00189567735	Recessive	Cultivar vs YUC-W	**
SIFT4G	1020.686441	830.6331885	1210.739693	0	Recessive	LR1 vs YUC-W	**
SIFT4G	408.8364407	218.7831885	598.8896928	5.12E-09	Recessive	LR2 vs YUC-W	**
SIFT4G	1106.763822	970.0954332	1243.43221	0	Recessive	GD vs YUC-W	**
SIFT4G	970.5114407	707.0373743	1233.985507	0	Recessive	PR vs YUC-W	**
SIFT4G	900.5237901	831.0412884	970.0062918	0	Recessive	FL vs YUC-W	**
SIFT4G	571.5989407	466.0220498	677.1758316	0	Recessive	YUC-E vs YUC-W	**

** *P*-adjust < 0.01

Table S7. List of 109 cultivar accessions used for the domestication scan.

Species	Individual	Sequence depth	NCBI accession
G. hirsutum	AD1_Cultivar_B11SA2910	13.22	SRR6311857
G. hirsutum	AD1_Cultivar_B11SA1044	14.64	SRR6311537
G. hirsutum	AD1_Cultivar_B11SA2580	14.89	SRR6311737
G. hirsutum	AD1_Cultivar_B11SA3251	14.98	SRR6311854
G. hirsutum	AD1_Cultivar_B11SA0982	15.33	SRR6311536
G. hirsutum	AD1_Cultivar_B11DIV063	15.38	SRR6311591
G. hirsutum	AD1_Cultivar_B11SA1205	15.4	SRR6311571
G. hirsutum	AD1_Cultivar_B11SA1330	15.49	SRR6311675
G. hirsutum	AD1_Cultivar_B11DIV136	15.51	SRR6311642
G. hirsutum	AD1_Cultivar_B11DIV033	15.54	SRR6311582
G. hirsutum	AD1_Cultivar_B11SA3348	15.54	SRR6311868
G. hirsutum	AD1_Cultivar_B11SA1139	15.55	SRR6311541
G. hirsutum	AD1_Cultivar_B11SA2375	15.79	SRR6311734
G. hirsutum	AD1_Cultivar_B11SA0940	15.9	SRR6311819
G. hirsutum	AD1_Cultivar_B11SA3332	16.08	SRR6311860
G. hirsutum	AD1_Cultivar_B11DIV298	16.17	SRR6311848
G. hirsutum	AD1_Cultivar_B11DIV232	16.26	SRR6311561
G. hirsutum	AD1_Cultivar_B11SA0159	16.56	SRR6311842
G. hirsutum	AD1_Cultivar_B11SA1766	16.6	SRR6311496
G. hirsutum	AD1_Cultivar_B11SA1019	16.62	SRR6311538
G. hirsutum	AD1_Cultivar_B11SA1913	16.72	SRR6311498
G. hirsutum	AD1_Cultivar_P111DP5690	16.77	SRR1975548
G. hirsutum	AD1_Cultivar_B11SA2356	16.8	SRR6311732
G. hirsutum	AD1_Cultivar_B11SA1181	16.83	SRR6311569
G. hirsutum	AD1_Cultivar_B11SA2057	16.9	SRR6311497
G. hirsutum	AD1_Cultivar_B11SA2269	16.97	SRR6311507
G. hirsutum	AD1_Cultivar_B11DIV245	16.99	SRR6311681
G. hirsutum	AD1_Cultivar_B11DIV322	17.1	SRR6311840
G. hirsutum	AD1_Cultivar_B11SA0881	17.17	SRR6311828
G. hirsutum	AD1_Cultivar_B11SA0718	17.69	SRR6311824
G. hirsutum	AD1_Cultivar_B11DIV306	17.85	SRR6311847
G. hirsutum	AD1_Cultivar_B11DIV272	18.09	SRR6311845
G. hirsutum	AD1_Cultivar_B11DIV230	18.11	SRR6311556
G. hirsutum	AD1_Cultivar_B11SA0892	18.4	SRR6311826
G. hirsutum	AD1_Cultivar_B11DIV206	18.61	SRR6311557
G. hirsutum	AD1_Cultivar_B11SA1138	18.63	SRR6311542
G. hirsutum	AD1_Cultivar_B11DIV184	18.69	SRR6311650
G. hirsutum	AD1_Cultivar_B11DIV141	18.73	SRR6311641
G. hirsutum	AD1_Cultivar_B11DIV363	18.76	SRR6311686
G. hirsutum	AD1_Cultivar_B11SA1759	19.08	SRR6311501
G. hirsutum	AD1_Cultivar_B11SA3493	19.08	SRR6311740
G. hirsutum	AD1_Cultivar_B11DIV310	19.15	SRR6311519
G. hirsutum	AD1_Cultivar_B11SA3403	19.17	SRR6311865
G. hirsutum	AD1_Cultivar_B11SA1156	19.29	SRR6311534
G. hirsutum	AD1_Cultivar_B11SA2401	19.52	SRR6311735
G. hirsutum	AD1_Cultivar_B11DIV211	19.53	SRR6311555
G. hirsutum	AD1_Cultivar_B11SA0300	19.57	SRR6311714
G. hirsutum	AD1_Cultivar_B11DIV074	19.61	SRR6311576
G. hirsutum	AD1_Cultivar_B11SA3444	19.65	SRR6311863
G. hirsutum	AD1_Cultivar_B11DIV119	19.66	SRR6311575
G. hirsutum	AD1_Cultivar_B11SA1263	19.72	SRR6311563
G. hirsutum	AD1_Cultivar_B11SA3284	19.79	SRR6311852
G. hirsutum	AD1_Cultivar_B11SA2192	19.81	SRR6311605
G. hirsutum	AD1_Cultivar_B11DIV096	19.9	SRR6311644
G. hirsutum	AD1_Cultivar_B11SA1403	20	SRR6311680
G. hirsutum	AD1_Cultivar_B11SA3413	20.01	SRR6311864
G. hirsutum	AD1_Cultivar_B11DIV366	20.05	SRR6311687
G. hirsutum	AD1_Cultivar_B11SA0186	20.35	SRR6311717
G. hirsutum	AD1_Cultivar_B11SA1349	20.45	SRR6311674

G. hirsutum	AD1_Cultivar_B11SA3781	20.49	SRR6311745
G. hirsutum	AD1_Cultivar_B11SA3452	20.5	SRR6311862
G. hirsutum	AD1_Cultivar_B11DIV134	20.62	SRR6311643
G. hirsutum	AD1_Cultivar_B11SA0298	20.62	SRR6311715
G. hirsutum	AD1_Cultivar_B11DIV308	20.71	SRR6311850
G. hirsutum	AD1_Cultivar_B11SA0825	20.72	SRR6311822
G. hirsutum	AD1_Cultivar_B11DIV012	20.76	SRR6311585
G. hirsutum	AD1_Cultivar_B11DIV260	20.77	SRR6311513
G. hirsutum	AD1_Cultivar_B11SA3749	20.81	SRR6311742
G. hirsutum	AD1_Cultivar_B11DIV042	20.88	SRR6311590
G. hirsutum	AD1_Cultivar_B11SA2149	21.22	SRR6311606
G. hirsutum	AD1_Cultivar_B11DIV106	21.29	SRR6311690
G. hirsutum	AD1_Cultivar_B11SA0582	21.34	SRR6311710
G. hirsutum	AD1_Cultivar_B11SA1427	21.59	SRR6311783
G. hirsutum	AD1_Cultivar_B11SA1392	21.79	SRR6311673
G. hirsutum	AD1_Cultivar_B11DIV007	21.8	SRR6311682
G. hirsutum	AD1_Cultivar_B11DIV373	22	SRR6311689
G. hirsutum	AD1_Cultivar_B11SA0311	22.13	SRR6311713
G. hirsutum	AD1_Cultivar_B11SA0857	22.15	SRR6311823
G. hirsutum	AD1_Cultivar_B11DIV316	22.29	SRR6311849
G. hirsutum	AD1_Cultivar_B11SA0815	22.39	SRR6311825
G. hirsutum	AD1_Cultivar_B11DIV226	22.85	SRR6311518
G. hirsutum	AD1_Cultivar_B11SA0165	22.96	SRR6311718
G. hirsutum	AD1_Cultivar_B11SA1465	22.98	SRR6311779
G. hirsutum	AD1_Cultivar_B11SA1184	23.05	SRR6311570
G. hirsutum	AD1_Cultivar_B11DIV055	23.52	SRR6311578
G. hirsutum	AD1_Cultivar_B11SA3748	23.68	SRR6311741
G. hirsutum	AD1_Cultivar_B11DIV199	23.82	SRR6311573
G. hirsutum	AD1_Cultivar_B11SA3338	24.31	SRR6311870
G. hirsutum	AD1_Cultivar_B11DIV176	24.56	SRR6311646
G. hirsutum	AD1_Cultivar_B11DIV256	24.76	SRR6311554
G. hirsutum	AD1_Cultivar_B11SA0238	25.1	SRR6311716
G. hirsutum	AD1_Cultivar_B11SA0084	26.27	SRR6311821
G. hirsutum	AD1_Cultivar_B11DIV138	26.42	SRR6311574
G. hirsutum	AD1_Cultivar_B11DIV273	26.44	SRR6311514
G. hirsutum	AD1_Cultivar_B11DIV037	27.2	SRR6311580
G. hirsutum	AD1_Cultivar_B11DIV380	27.37	SRR6311688
G. hirsutum	AD1_Cultivar_P111PD1	31.08	SRR1534918
G. hirsutum	AD1_Cultivar_B11DIV243	31.34	SRR6311516
G. hirsutum	AD1_Cultivar_P111TamSph	31.84	SRR1536368
G. hirsutum	AD1_Cultivar_P111TM1	32.35	SRR1534688
G. hirsutum	AD1_Cultivar_P111FM832	33.1	SRR1536364
G. hirsutum	AD1_Cultivar_P111SL542	33.19	SRR1534862
G. hirsutum	AD1_Cultivar_P111ST474	35.07	SRR1536367
G. hirsutum	AD1_Cultivar_P111SG747	36.98	SRR1536366
G. hirsutum	AD1_Cultivar_P111240RNR	37.05	SRR1536363
G. hirsutum	AD1_Cultivar_B11DIV178	43.42	SRR6311645
G. hirsutum	AD1_Cultivar_B11DIV048	47.09	SRR6311579
G. hirsutum	AD1_Cultivar_B11DIV066	47.96	SRR6311584
G. hirsutum	AD1_Cultivar_B11DIV144	48.24	SRR6311648

***Table S4.** Raw KmerCity output for selected individuals, with their genetic group indicated in the first column. The second column lists each individual, the top row lists the input FASTA files, and the second row shows the total number of kmers in each FASTA file. Remaining cells indicate the frequency of each kmer from the corresponding FASTA in each sample.

***Table S8.** List of genes with hits in the Swiss-Prot database. Some UniProt IDs are duplicated due to the presence of isoforms or paralogous genes.

* Tables S4 and S8 have been deposited in a public GitHub repository, due to their large size: https://github.com/Wendellab/Wildcotton_YUCFL/blob/main/99_supplemental/Suppl_TableS1toS7_FLYUC_wildcotton_revised.xlsx

SI References

1. H. Cheng, G. T. Concepcion, X. Feng, H. Zhang, H. Li, Haplotype-resolved de novo assembly using phased assembly graphs with hifiasm. *Nat. Methods* **18**, 170–175 (2021).
2. H. Li, Aligning sequence reads, clone sequences and assembly contigs with BWA-MEM. *arXiv [q-bio.GN]* (2013).
3. G. G. Faust, I. M. Hall, SAMBLASTER: fast duplicate marking and structural variant read extraction. *Bioinformatics* **30**, 2503–2505 (2014).
4. H. Li, *et al.*, The Sequence Alignment/Map format and SAMtools. *Bioinformatics* **25**, 2078–2079 (2009).
5. O. Dudchenko, *et al.*, De novo assembly of the Aedes aegypti genome using Hi-C yields chromosome-length scaffolds. *Science* **356**, 92–95 (2017).
6. N. C. Durand, *et al.*, Juicebox provides a visualization system for Hi-C contact maps with unlimited zoom. *Cell Syst.* **3**, 99–101 (2016).
7. B. Hendrix, J. M. Stewart, Estimation of the nuclear DNA content of *Gossypium* species. *Ann. Bot.* **95**, 789–797 (2005).
8. C. E. Grover, *et al.*, The *Gossypium longicalyx* genome as a resource for cotton breeding and evolution. *G3 (Bethesda)* **10**, 1457–1467 (2020).
9. H. Li, Minimap2: pairwise alignment for nucleotide sequences. *Bioinformatics* **34**, 3094–3100 (2018).
10. E. S. Ricemeyer, R. A. Carroll, W. C. Warren, Agptools: a utility suite for editing genome assemblies. *Bioinformatics* **41** (2025).
11. S. Ou, *et al.*, Benchmarking transposable element annotation methods for creation of a streamlined, comprehensive pipeline. *Genome Biol.* **20**, 275 (2019).
12. L. Gabriel, *et al.*, BRAKER3: Fully automated genome annotation using RNA-seq and protein evidence with GeneMark-ETP, AUGUSTUS, and TSEBRA. *Genome Res.* **34**, 769–777 (2024).
13. D. Nishimura, RepeatMasker. *Biotech Softw. Internet Rep.* **1**, 36–39 (2000).
14. D. Kuznetsov, *et al.*, OrthoDB v11: annotation of orthologs in the widest sampling of organismal diversity. *Nucleic Acids Res.* **51**, D445–D451 (2023).
15. M. Seppey, M. Manni, E. M. Zdobnov, BUSCO: Assessing genome assembly and annotation completeness. *Methods Mol. Biol.* **1962**, 227–245 (2019).
16. F. Tegenfeldt, *et al.*, OrthoDB and BUSCO update: annotation of orthologs with wider sampling of genomes. *Nucleic Acids Res.* **53**, D516–D522 (2025).
17. C. E. Grover, Y. Yu, R. A. Wing, A. H. Paterson, J. F. Wendel, A phylogenetic analysis of indel dynamics in the cotton genus. *Mol. Biol. Evol.* **25**, 1415–1428 (2008).
18. D. Kahle, H. Wickham, ggmap: Spatial visualization with ggplot2. (2013).
19. W. Ning, *et al.*, Origin and diversity of the wild cottons (*Gossypium hirsutum*) of Mound Key, Florida. *Sci. Rep.* **14**, 1–12 (2024).
20. W. Ning, *et al.*, Comparative population genomics of relictual Caribbean Island *Gossypium hirsutum*. *Mol. Ecol.* **35**, e70239 (2026).
21. D. Yuan, *et al.*, Parallel and intertwining threads of domestication in allopolyploid cotton. *Adv. Sci.* (2021). https://doi.org/10.1002/advs.202003634.
22. K. I. Kendig, *et al.*, Sentieon DNAseq variant calling workflow demonstrates strong computational performance and accuracy. *Front. Genet.* **10**, 736 (2019).
23. A. M. Bolger, M. Lohse, B. Usadel, Trimmomatic: a flexible trimmer for Illumina sequence data. *Bioinformatics* **30**, 2114–2120 (2014).
24. H. Li, A statistical framework for SNP calling, mutation discovery, association mapping and population genetical parameter estimation from sequencing data. *Bioinformatics* **27**, 2987–2993 (2011).
25. P. Danecek, *et al.*, The variant call format and VCFtools. *Bioinformatics* **27**, 2156–2158

(2011).
26. P. Danecek, *et al.*, Twelve years of SAMtools and BCFtools. *Gigascience* **10** (2021).
27. S. Purcell, *et al.*, PLINK: a tool set for whole-genome association and population-based linkage analyses. *Am. J. Hum. Genet.* **81**, 559–575 (2007).
28. R. Team, R: A language and environment for statistical computing. *MSOR connections* **1** (2014).
29. H. Wickham, *Ggplot2: Elegant graphics for data analysis*, 2nd Ed. (Springer International Publishing, 2016).
30. G. Yu, D. K. Smith, H. Zhu, Y. Guan, T. T.-Y. Lam, ggtree: An R package for visualization and annotation of phylogenetic trees with their covariates and other associated data. *Methods Ecol. Evol.* **8**, 28–36 (2017).
31. E. Frichot, O. François, LEA: An R package for landscape and ecological association studies. *Methods Ecol. Evol.* (2015). https://doi.org/10.1111/2041-210X.12382.
32. J. K. Pickrell, J. K. Pritchard, Inference of population splits and mixtures from genome-wide allele frequency data. *PLoS Genet.* **8**, e1002967 (2012).
33. M. Malinsky, M. Matschiner, H. Svardal, Dsuite - Fast D-statistics and related admixture evidence from VCF files. *Mol. Ecol. Resour.* **21**, 584–595 (2021).
34. R. Maier, *et al.*, On the limits of fitting complex models of population history to *f*-statistics. *Elife* **12**, e85492 (2023).
35. C. L. Brubaker, J. A. Koontz, J. F. Wendel, Bidirectional cytoplasmic and nuclear introgression in the New World cottons *Gossypium barbadense* and *G. hirsutum* (Malvaceae). *Am. J. Bot.* **80**, 1203–1208 (1993).
36. J. F. Wendel, C. L. Brubaker, A. E. Percival, Genetic diversity in *Gossypium hirsutum* and the origin of upland cotton. *Am. J. Bot.* **79**, 1291–1310 (1992).
37. Z. Yang, *et al.*, Graph pan-genome illuminates evolutionary trajectories and agronomic trait architecture in allotetraploid cotton. *Nat. Genet.* (2026). https://doi.org/10.1038/s41588-025-02462-1.
38. S. R. Browning, R. K. Waples, B. L. Browning, Fast, accurate local ancestry inference with FLARE. *Am. J. Hum. Genet.* **110**, 326–335 (2023).
39. B. L. Browning, X. Tian, Y. Zhou, S. R. Browning, Fast two-stage phasing of large-scale sequence data. *Am. J. Hum. Genet.* **108**, 1880–1890 (2021).
40. K. Zhang, *et al.*, High-density linkage map construction and QTL analyses for fiber quality, yield and morphological traits using CottonSNP63K array in upland cotton (*Gossypium hirsutum* L.). *BMC Genomics* **20**, 889 (2019).
41. O. Garsmeur, *et al.*, The genomic footprints of wild *Saccharum* species trace domestication, diversification, and modern breeding of sugarcane. *Cell* **188**, 7252–7266.e15 (2025).
42. D. E. Wood, J. Lu, B. Langmead, Improved metagenomic analysis with Kraken 2. *Genome Biol.* **20**, 257 (2019).
43. J.-J. Jin, *et al.*, GetOrganelle: A fast and versatile toolkit for accurate de novo assembly of organelle genomes. *Genome Biol.* **21**, 241 (2020).
44. X.-L. Yan, *et al.*, Genetic diversity and evolution of the plastome in allotetraploid cotton (*Gossypium* spp.). *J. Syst. Evol.* **62**, 1118–1136 (2024).
45. K. Katoh, D. M. Standley, MAFFT multiple sequence alignment software version 7: improvements in performance and usability. *Mol. Biol. Evol.* **30**, 772–780 (2013).
46. S. Capella-Gutiérrez, J. M. Silla-Martínez, T. Gabaldón, trimAl: a tool for automated alignment trimming in large-scale phylogenetic analyses. *Bioinformatics* **25**, 1972–1973 (2009).
47. B. Q. Minh, J. Trifinopoulos, D. Schrempf, H. A. Schmidt, R. Lanfear, IQ-TREE version 2.0: tutorials and manual phylogenomic software by maximum likelihood. *URL http://www. iqtree. org* (2019).

48. T. Junier, E. M. Zdobnov, The Newick utilities: high-throughput phylogenetic tree processing in the UNIX shell. *Bioinformatics* **26**, 1669–1670 (2010).
49. N. Bailey, L. Stevison, K. Samuk, Correcting for bias in estimates of θw and Tajima's D from missing data in next-generation sequencing. *Mol. Ecol. Resour.* **25**, e14104 (2025).
50. F. Biscarini, P. Cozzi, G. Gaspa, G. Marras, detectRUNS: Detect runs of homozygosity and runs of heterozygosity in diploid genomes. (2018).
51. S. H. Min, J. Zhou, Smplot: An R package for easy and elegant data visualization. *Front. Genet.* **12**, 802894 (2021).
52. A. C. Roulin, Blinded by the lights? Re-examining the adaptive role of transposable elements in plants with population genomics. *Curr. Opin. Plant Biol.* **89**, 102846 (2025).
53. L. Campos-Dominguez, R. Castanera, C. E. Grover, J. F. Wendel, J. M. Casacuberta, Differential LTR-retrotransposon dynamics across polyploidization, speciation, domestication, and improvement of cotton (*Gossypium*). *Genome Biol.* **26**, 369 (2025).
54. W. Shen, S. Le, Y. Li, F. Hu, SeqKit: A cross-platform and ultrafast toolkit for FASTA/Q file manipulation. *PLoS One* **11**, e0163962 (2016).
55. C. C. Menard, *et al.*, SWIF-TE: identifying novel transposable element insertions from short read data. *bioRxiv* (2025).
56. J. Gonçalves-Dias, A. Singh, C. Graf, M. G. Stetter, Genetic incompatibilities and evolutionary rescue by wild relatives shaped grain amaranth domestication. *Mol. Biol. Evol.* **40**, msad177 (2023).
57. J. L. Conover, J. F. Wendel, Deleterious mutations accumulate faster in allopolyploid than diploid cotton (*Gossypium*) and unequally between subgenomes. *Mol. Biol. Evol.* **39**, msac024 (2022).
58. E. V. Davydov, *et al.*, Identifying a high fraction of the human genome to be under selective constraint using GERP++. *PLoS Comput. Biol.* **6**, e1001025 (2010).
59. R. Vaser, S. Adusumalli, S. N. Leng, M. Sikic, P. C. Ng, SIFT missense predictions for genomes. *Nat. Protoc.* **11**, 1–9 (2016).
60. A. Bairoch, R. Apweiler, The SWISS-PROT protein sequence database and its supplement TrEMBL in 2000. *Nucleic Acids Res.* **28**, 45–48 (2000).
61. J. Jiang, *et al.*, Incorporating genetic load contributes to predicting *Arabidopsis thaliana*'s response to climate change. *Nat. Commun.* **16**, 2752 (2025).
62. L. Wang, *et al.*, The interplay of demography and selection during maize domestication and expansion. *Genome Biol.* **18**, 215 (2017).
63. J. Armstrong, *et al.*, Progressive Cactus is a multiple-genome aligner for the thousand-genome era. *Nature* **587**, 246–251 (2020).
64. C. D. Mirchandani, *et al.*, A fast, reproducible, high-throughput variant calling workflow for population genomics. *Mol. Biol. Evol.* **41**, msad270 (2024).
65. H. Chen, N. Patterson, D. Reich, Population differentiation as a test for selective sweeps. *Genome Res.* **20**, 393–402 (2010).
66. M. Lawrence, R. Gentleman, V. Carey, rtracklayer: an R package for interfacing with genome browsers. *Bioinformatics* **25**, 1841–1842 (2009).
67. S. Ahmad, *et al.*, The UniProt website API: facilitating programmatic access to protein knowledge. *Nucleic Acids Res.* **53**, W547–W553 (2025).
68. P. D. Thomas, *et al.*, PANTHER: Making genome-scale phylogenetics accessible to all. *Protein Sci.* **31**, 8–22 (2022).
69. S. Díaz-Cruz, *et al.*, Tri-trophic consequences of plant-to-plant volatile signalling and its contingency on plant relatedness in wild cotton. *Ann. Bot.* (2025). https://doi.org/10.1093/aob/mcaf315.
